# Integrated metabolomic and transcriptomic analysis of the anthocyanin and proanthocyanidin regulatory networks in red walnut natural hybrid progeny leaves

**DOI:** 10.7717/peerj.14262

**Published:** 2022-10-20

**Authors:** Lei Wang, Lin Li, Wei Zhao, Lu Fan, Haijun Meng, Ganggang Zhang, Wenjiang Wu, Jiangli Shi, Guoliang Wu

**Affiliations:** Henan Agricultural University, Zhengzhou, China

**Keywords:** Transcriptome, Metabolome, Anthocyanin, Proanthocyanidin, Red walnut

## Abstract

**Background:**

Walnuts are among the most important dry fruit crops worldwide, typically exhibiting green leaves and yellow–brown or gray–yellow seed coats. A specific walnut accession with red leaves and seed coats, ‘RW-1’, was selected for study because of its high anthocyanin and proanthocyanidin (PA) contents. Anthocyanins and PAs are important secondary metabolites and play key roles in plant responses to biotic and abiotic stresses. However, few studies have focused on the molecular mechanism of anthocyanin biosynthesis in walnuts.

**Methods:**

In this study, we determined the anthocyanin and PA components and their contents in different color leaves of ‘RW-1’ natural hybrid progenies at various developmental stages. Integrated transcriptome and metabolome analyses were used to identify the differentially expressed genes (DEGs) and differentially accumulated metabolites (DAMs). We also performed conjoint analyses on DEGs and DAMs to ascertain the degree pathways, and explore the regulation of anthocyanin and PA biosynthesis.

**Results:**

The results of widely targeted metabolome profiling and anthocyanin detection revealed 395 substances, including four PAs and 26 anthocyanins, in red (SR) and green leaves (SG) of ‘RW-1’ natural hybrid progenies. From the research, the contents of all anthocyanin components in SR were higher than that in SG. Among them, the contents of delphinidin 3-O-galactoside, cyanidin 3-O-galactoside, delphinidin 3-O-arabinoside and cyanidin 3-O-glucoside were significantly higher than others, and they were considered as the main types of anthocyanins. However, nine anthocyanins were detected only in SR. For PAs, the content of procyanidin C1 was higher in SR compared with SG, while procyanidin B1 and procyanidin B3 were higher in SR-1 and SR-3 but downregulated in SR-2 compared with the controls. Furthermore, transcriptome analysis revealed that the expressions of structural genes (*C4H*, *F3H*, *F3′5′H*, *UFGT*,* LAR* and* ANR*), three *MYBs* predicted as the activators of anthocyanin and PA biosynthesis, two *MYBs* predicted as the repressors of anthocyanin biosynthesis, and five *WD40s* in the anthocyanin and PA biosynthetic pathways were significantly higher in the SR walnuts. Gene-metabolite correlation analyses revealed a core set of 31 genes that were strongly correlated with four anthocyanins and one PA metabolites. The alteration of gene coding sequence altered the binding or regulation of regulatory factors to structural genes in different color leaves, resulting in the effective increase of anthocyanins and PAs accumulation in red walnut.

**Conclusions:**

This study provides valuable information on anthocyanin and PA metabolites and candidate genes for anthocyanin and PA biosynthesis, yielding new insights into anthocyanin and PA biosynthesis in walnuts.

## Introduction

Anthocyanins and proanthocyanidins (PAs) are important secondary metabolites. To date, more than 635 kinds of anthocyanins and 680 kinds of PAs have been identified (Cho et al., 2016). Anthocyanins are among the most important color-presenting materials in flavonoids, mainly found in the leaves, flowers, and fruits of higher plants, and are formed by the combination of anthocyanidins and glycosyls (Honda & Moriya, 2018). Cyanidin (Cy), delphinidin (Dp), pelargonidin (Pg), peonidin (Pn), petunidin (Pt), and malvidin (Mv) are six common anthocyanidins, and common glycosyls include glucose, galactose, and sucrose, among others (Sasaki et al., 2014). Various types and amounts of glycosyls bound to separate positions of anthocyanidin resulted in a significant increase in anthocyanin compounds (Shi & Xie, 2014). PAs are oligomers or polymers polymerized by flavane-3-alcohol subunits, and their degree of polymerization may vary according to plant species (Marles, Ray & Gruber, 2003). The structural changes of PAs depend on the properties of the flavan-3-alcohol initiator and extension unit, the position and stereo configuration of the link with the lower unit, the degree of polymerization, and whether there is modification. These factors increase the structural diversity of PAs (Dixon, Xie & Sharma, 2004). Anthocyanins and PAs act to protect plants from ultraviolet ray damage, help plants resist low temperatures (Li et al., 2020a; Li et al., 2020b; Li et al., 2020c; Jun et al., 2018), and contribute to colors (Saigo et al., 2020).

The anthocyanin and PA biosynthesis pathways have been well characterized in some plants (Katsumoto et al., 2007). Generally, phenylalanine metabolism is regarded as the initial step in the biosynthesis of these two substances. This step involves a series of enzymatic reactions, including reactions of phenylalanine ammonia lyase (PAL), cinnamate 4-hydroxylase (C4H), chalcone synthetase (CHS), chalcone isoenzyme (CHI), flavanone 3-hydroxylase (F3H), flavonoid 3′,5′-hydroxylase (F3′5′H), and dihydroflavonol 4-reductase (DFR). Leucoanthocyanidins and anthocyanidins are two important branch points between the anthocyanin and PA biosynthesis pathways. Anthocyanin is biosynthesized by leucoanthocyanidin dioxygenase (LDOX)/anthocyanidin synthetase (ANS) and UDP glucose:flavonoid-3-O-glucosyltransferase (UFGT) (Caputi et al., 2012; Yonekura-Sakakibara et al., 2012), while PAs are biosynthesized by leucoanthocyanidin reductase (LAR) and anthocyanidin reductase (ANR) (Zhang et al., 2020a; Zhang et al., 2020b).

The structural genes involved in anthocyanin and PA biosynthesis are regulated by multiple transcription factors (TFs), among which MYB TFs have been widely studied. The grape R2R3-MYB TFs VvMYBA1, VvMYBA6 and VvMYBA7 regulate anthocyanin biosynthesis by activating *UFGT* and *3AT* expressions (Matus et al., 2017). Overexpression of *MdMYB3* increases anthocyanin accumulation in tobacco by activating the expressions of *NtCHI, NtCHS, NtANS, NtUFGT, NtAn2,* and *NtCOMT* (Vimolmangkang et al., 2013). Overexpression of *GhMYB1a* in gerbera and tobacco (*Nicotiana tabacum*) decreased anthocyanin accumulation and increased flavonol accumulation by upregulating the structural genes involved in flavonol biosynthesis (Zhong et al., 2020). For PA biosynthesis, AtTT2, a positive R2R3 MYB regulator in *Arabidopsis*, showed the same expression pattern as *AtANR* and influenced the PAs accumulation in seed coats (Nesi et al., 2001). Moreover, there have been a large number of reports on *AtTT2* homologous genes in other species, including *VvMybPA1* and *VvMybPA2* in *Vitis vinifera* (Bogs et al., 2007; Terrier et al., 2009), *FtMYB1* and *FtMYB2* in *Fagopyrum tataricum* (Bai et al., 2014), *MtMYB14* in *Medicago truncatula* (Liu, Jun & Dixon, 2014), and *GhMYB10* and *GhMYB36* in *Gossypium hirsutum* (Lu, Roldan & Dixon, 2017).

MYB regulates anthocyanin and PA biosynthesis either alone or by forming the MBW complex (MYB-bHLH-WD40) (Hichri et al., 2011; Springob et al., 2003). For example, AcMYB123 and AcbHLH42 in *Actinidia chinensis* cv. Hongyang promote anthocyanin accumulation by activating the promoters of *AcANS* and *Ac3FGT1* (Wang et al., 2019). *PyMYB10* and *PyMYB114* co-transformed with *PybHLH3* induced the promoter activity of *PyDFR*, *PyANS*, *PyUFGT*, *PyGST* and *PyABC* transporters (Li et al., 2020a; Li et al., 2020b; Li et al., 2020c). Composed of AtMYB123 (TT2), AtbHLH42 (TT8), and AtTTG1 (WD40-protein) in *Arabidopsis*, the complex was reported to activate the expression of *DFR*, *LDOX*, and *ANR*, which led to the accumulation of PAs in the seed coat (Baudry et al., 2004). FaMYB9/11, FabHLH3, and FaTTG1 in strawberry (Schaart et al., 2013) and DkMYB2/4, DkMYC1, and DkWDR1 in persimmon (del Mar Naval et al., 2016) are transcriptional activation complexes of PA biosynthesis pathways, which indicates that MBW complexes are well conserved in plants (Xu, Dubos & Lepiniec, 2015). Therefore, this study aimed to elucidate the regulatory mechanism, including the main structural genes and TFs involved, of anthocyanins and PAs accumulation in red and green walnuts.

Walnut (*Juglans regia* L.) is an ancient fruit tree belonging to the Juglandaceae family. Walnuts rank among the four most consumed nuts worldwide, and walnut trees are ecologically important. The walnut industry is a core component of industrial poverty alleviation in many areas and plays an important role in reducing poverty and increasing farmers’ income. The phenotypic traits of different walnut varieties are similar, with green leaves and yellow–brown or gray–yellow seed coats. Recently, ‘RW-1’, a germplasm resource of Chinese wild red walnut, was found by our research group to have red leaves, pericarp, seed coats and xylem owing to its high anthocyanin content (Li et al., 2018). In our previous studies, we found that the growth of red walnut was lower than that of ordinary walnut because of weak photosynthesis (Yang et al., 2020b). The enrichment of anthocyanins in red walnut not only improves fruit quality but also weakens the growth potential of trees and reduces the need for pruning, which is beneficial in the development of dwarf and densely planted fruit trees. Although several genes, such as *bHLHs* and *CHSs*, have been found to be involved in anthocyanin biosynthesis (Zhao et al., 2021a; Zhao et al., 2021b), the molecular mechanism of anthocyanin and PA biosynthesis has not yet been clearly elucidated in red walnut. Seeds of red walnut give rise to natural hybrid progeny with character separation: red leaves (SR) and green leaves (SG).

In recent years, combined analysis of the transcriptome and metabolome has been widely used to shed light on anthocyanin and PA biosynthesis and accumulation in plants (Zhang et al., 2020a; Zhang et al., 2020b; Shi et al., 2020; Solhaug et al., 2019; Yang et al., 2020a; Yang et al., 2020b; Zhang et al., 2020a; Zhang et al., 2020b; Zhuang et al., 2019; Li et al., 2020a; Li et al., 2020b; Li et al., 2020c). In the current study, the regulatory networks of anthocyanin and PA biosynthesis in walnut were constructed using two RW-1 natural hybrid accessions with red and green leaves. Comparative analysis of metabolomic and transcriptomic data was performed to elucidate the pathways of anthocyanin and PA metabolism and to identify differentially expressed genes (DEGs) associated with walnut anthocyanin and PA biosynthesis.

## Materials & Methods

### Plant materials and growth conditions

**Figure 1:**
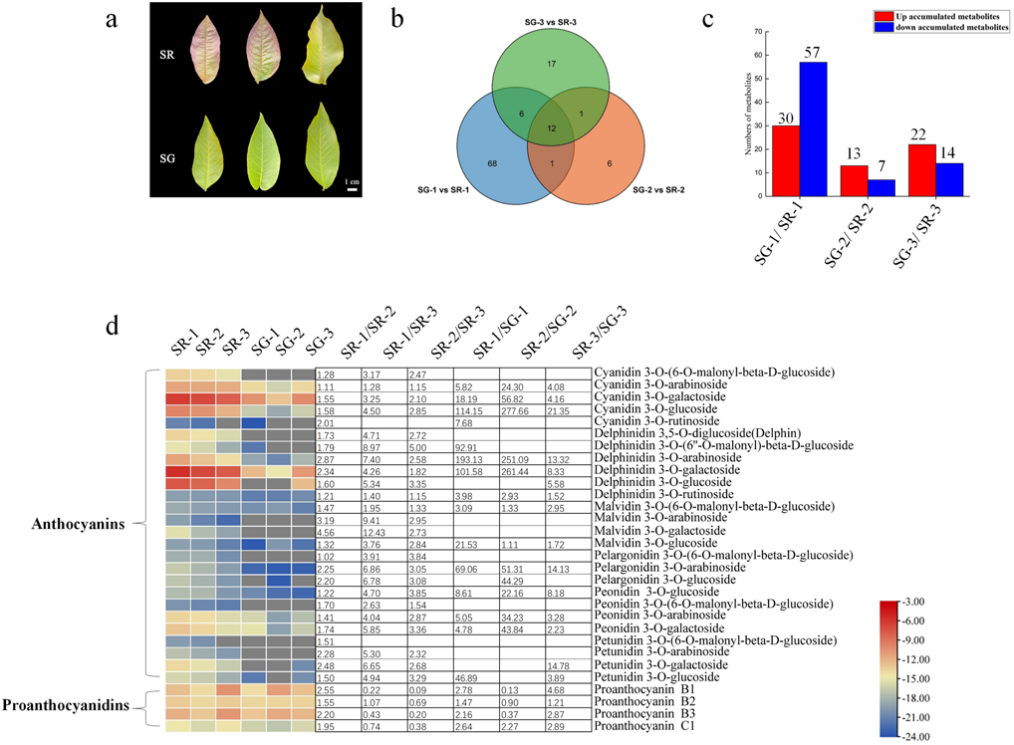
Metabolite profiles between SG and SR. (A) Morphological observation of leaves of different colors in natural hybrid progenies of red walnut. (B) Venn diagram depicting the shared and specific metabolites of the three compared groups of leaf samples. (C) Number of differentially accumulated metabolites (DAMs) in the SG-1 *vs.* SR-1, SG-2 *vs.* SR-2 and SG-3 *vs.* SR-3 comparisons. (D) Components and contents of anthocyanins and proanthocyanidins in the leaves of SG and SR. Numbers refer to fold changes in anthocyanin contents. The criteria for significant differences in metabolite levels between the two samples were a variable importance in projection (VIP) value ≥ 1 and an absolute Log2FC (fold change) ≥ 1. The scale bars show the log2-transformed metabolite contents.

Chinese wild red walnut (*J. regia* L. accession RW-1, germplasm resource number: JUREG4108210002) was introduced from Taihang Mountain, China (Fig. S1). To maintain a relatively consistent genetic background, natural hybrid plants with different color phenotypes (SG for natural hybrid plants with green leaves and SR for natural hybrid plants with red leaves) were grown at the Fruit Tree Experimental Station of Horticulture College, Henan Agricultural University, Zhengzhou, Henan, China. Sampling permission was obtained from the public land management agency of Henan Agricultural University. According to their color changes, SR walnut leaves were collected and sampled at the full red period (new shoot growth stage (SG-1 and SR-1)), red–green period (fruit swelling stage (SG-2 and SR-2)), and full green period (early period of fruit ripening (SG-3 and SR-3)). Leaves of SG walnuts from the same periods of development were collected as the control (Zhao et al., 2021b; Fig. 1A). All samples were immediately frozen in liquid nitrogen and stored at −80 °C until RNA and metabolite extraction.

### Extraction and quantification of metabolites

Widely targeted metabolome profiling and anthocyanin detection of the samples were performed at Wuhan MetWare Biotechnology Co., Ltd. (Wuhan, China), as previously described (Zhang et al., 2020a; Zhang et al., 2020b). In brief, 100 mg of leaves was crushed into powder and extracted with 0.6 mL of 70% aqueous methanol overnight at 4 °C (Viant et al., 2017). Following centrifugation at 10,000 g for 10 min, the supernatant was filtered through a microporous membrane (0.22 µm) for subsequent LC–MS/MS analysis.

Qualitative and quantitative mass spectrometry analysis of metabolites is based on the self-built MWBD (MetWare database) and public database of metabolite information and multiple reaction monitoring (MRM). Metabolite identification is based on the accurate mass of metabolites, MS2 fragments, MS2 fragments isotope distribution and retention time (RT). Through the company’s self-developed intelligent secondary spectrum matching method, the secondary spectrum and RT of the metabolites in the project samples are compared with the company’s The database secondary spectrum and RT are intelligently matched one by one, and the MS tolerance and MS2 tolerance are set to 2 ppm and 5 ppm, respectively. Finally, through the ABSciexQTRAPLC-MS/MS detection platform, the peak area data of metabolites in samples were obtained by using the MRM of triple quadrupole mass spectrometry, and the relative content of metabolites in different samples was obtained.

PCA was performed to verify the differences and reliability of metabolites in all the samples. DAMs between groups were filtered with a VIP value ≥1 and an absolute Log _2_FC (fold change) ≥1. Subsequently, the DAMs with significant enrichment were mapped to KEGG pathways (https://www.genome.jp/kegg), and a plant metabolic network (PMN, https://plantcyc.org/) was used to analyze the phenylalanine metabolism pathway, flavonoid metabolism pathway and anthocyanin biosynthesis pathway (Viant et al., 2017; Yi et al., 2020). We constructed metabolic pathways based on the KEGG database.

### RNA extraction, sequencing and transcriptome data analysis

Total RNA from walnut leaves was extracted using the Omega Plant RNA Kit (Omega Biotek, Norcross, GA, USA) according to the manufacturer’s instructions. The integrity and quality of total RNA were examined on 1% agarose gels, and the RNA concentrations were measured by a NanoDrop 1000 spectrophotometer (Thermo Fisher Scientific, Waltham, MA, United States). The cDNA libraries were constructed and sequenced on a HiSeq 2,500 platform (Illumina, San Diego, CA, United States) (NCBI accession PRJNA688391) by Biomarker Biotechnology Corp. (Beijing, China). Each sample (SG-1, SG-2, SG-3, SR-1, SR-2, SR-3) had three biological repeats and was sequenced separately. The raw data of fastq format were firstly processed through in-house perl scripts. At the same time, clean data were obtained by removing reads containing adapter, ploy-N and low quality reads. Furthermore, Q20, Q30, GC-content and sequence duplication level of the clean data were calculated. The filtered data were compared with the walnut reference genome (Chandler v2.0) (Marrano et al., 2020) using HISAT2 (Kim, Langmead & Salzberg, 2015). String Tie (Pertea et al., 2015) was then used to assemble and quantify results. A power analysis was performed by RNASeqpower in R package (Hart et al., 2013). The statistical power of this experimental design, calculated in RNASeqpower was greater than 0.92 (depth = 20, effect = 2, alpha = 0.05).

The expression abundance of genes was represented as fragments per kilobase of transcript per million fragments mapped (FPKM) values, and the DEGs (FDR <0.05 and — Log_2_FC — ≥ 1.5) between red leaves and green leaves were obtained using the DESeq 1.8.3 package (Yu et al., 2012). Mfuzz R package was used for clustering analysis of gene expression trend of DEGs. Functional annotation was performed using the KOBAS, and the KEGG pathways with significant enrichment were identified by employing the GOplot R package (Speer et al., 2020).

### Integrated metabolome and transcriptome analyses

Pearson’s correlation coefficients were calculated between the metabolome and transcriptome data. The coefficients were calculated from log2 (fold change) of each metabolite and log2 (fold change) of each transcript by the EXCEL program. Correlations with a coefficient of R^2^ >0.8 were selected. Metabolome and transcriptome relationships were visualized using Cytoscape (version 3.9.1).

### Quantitative Real-time (qRT) PCR assay

The expression of structural genes and TF genes in the anthocyanin biosynthesis pathway was examined by qRT–PCR. First-stand cDNA was synthesized using the FastQuant RT Kit (with gDNase) (Tiangen Biotech, Beijing, China), and qRT–PCR was performed using ChamQ Universal SYBR qPCR Master Mix (Vazyme, Nanjing, China) on an ABI 7500 Real-Time PCR system (Applied Biosystems, Foster City, CA, United States). Jr18S (GenBank accession number XM_019004991.1) was used as the housekeeping gene (*Yang et al., 2020*). Quantification was evaluated using the 2^−ΔΔCt^ method. All the primers are shown in Table S1.

### Gene amplification and sequence alignment

To clone the CDS of structural genes and TF genes in the anthocyanin and PA biosynthesis pathway, the specific primers for genes were designed using Primer 5.0. (Table S2). The cDNA of different leaves were used as template for PCR amplification. It was performed at 94 °C for 2 min followed by 35 cycles of 94 °C for 30 s, 55 °C for 30 s and 72 °C for 2 min. The target fragment was ligated to the pMD19-T cloning vector (Takara Bio Co., Ltd., China, Beijing) and subjected to blue-white spot screening. The positive bacteria were sent to Sangon Biotech (Shanghai) Co., Ltd. for sequencing. Sequence alignment was performed for the nucleotide sequences using DNAMAN 8.0.

### Statistical analysis

All data were analyzed using SPSS 22.0 software and are expressed as the mean ± standard deviation (SD) of three replicates. Significant differences were determined using a one-sided paired t test (^∗∗^*p*<0.01, ^∗^*p*<0.05) between red leaf and green leaf samples.

## Results

### Widely targeted metabolome profiling and anthocyanin detection in leaves of natural hybrid progenies of red walnut across developmental stages

To maintain consistency of the genetic background, the natural hybrid progenies of RW-1 with separate leaf colors (SG and SR) were investigated. During the young leaf stage, the SR walnut leaves were completely red, while the SG walnut leaves were green. The color difference between SR leaves and SG leaves became inconspicuous with leaf development. The SR leaves retained their red color only along the veins; in contrast, the SG leaves showed only a green color at all times (Fig. 1A).

The metabolome of 18 samples was profiled by a widely targeted metabolome approach, and anthocyanins were detected. A total of 395 compounds detected in the walnut leaves were classified into 19 classes (Tables S3, S4), including 26 anthocyanins and 4 PAs. The quality of the widely targeted metabolome data was reliable, as evidenced by the results of principal component analysis (PCA) (Figs. S2A, S2B), correlation analysis of population samples (Fig. S3) along the time course and two comparative analyses.

As Fig. 1B shows, 87, 20 and 36 differentially accumulated metabolites (DAMs) were identified in the SG-1 *vs.* SR-1, SG-2 *vs.* SR-2, and SG-3 *vs.* SR-3 comparisons, respectively. Furthermore, the histogram showed that the contents of 30 metabolites increased, those of 57 decreased in the first stage, those of 13 increased while those of seven decreased in the second stage, and those of 22 increased while those of 14 decreased in the third stage (Fig. 1C). The enrichment analysis of Kyoto Encyclopedia of Genes and Genomes (KEGG) pathways showed that the most significantly enriched pathway in all three stages was the anthocyanin biosynthesis pathway (Fig. S4), indicating that anthocyanins influenced leaf color development to some extent.

### Components and contents of flavonoids and anthocyanins during walnut leaf development stages

In this work, a total of 88 flavonoid metabolites were identified, including 55 flavonoids, three isoflavonoids, 26 anthocyanins and four PAs.

To determine whether the red pigmentation of red walnut is caused by anthocyanins, we analyzed the soluble anthocyanins in green and red leaves using an ultra-performance liquid chromatography–electrospray ionization–tandem mass spectrometry (UPLC–ESI–MS/MS) system. A total of 26 anthocyanins were detected in walnut leaves, and all contents were significantly higher in SR-1 than in SG-1; moreover, most anthocyanin contents were significantly higher in SR-1 than in SR-2 and SR-3 (Fig. 1D). Furthermore, delphinidin 3-*O*-galactoside, cyanidin 3-*O*-galactoside, delphinidin 3-O-arabinoside and cyanidin 3-*O*-glucoside were the primary components of anthocyanins because of their higher contents. We also found that nine anthocyanin compounds existed only in red leaves, including malvidin 3-*O*-galactoside, malvidin 3-*O*-arabinoside, cyanidin 3-*O*-(6-*O*-malonyl-beta-D-glucoside), delphinidin 3-*O*-glucoside, delphinidin 3,5-*O*-diglucoside (Delphin), peonidin 3-*O*-(6-*O*-malonyl-beta-D-glucoside), petunidin 3-*O*-(6-*O*-malonyl-beta-D-glucoside), and petunidin 3-*O*-arabinoside and pelargonidin 3-*O*-(6-*O*-malonyl-beta-D-glucoside).

PAs, including procyanidin B1, procyanidin B2, procyanidin B3 and procyanidin C1, were detected in the walnut leaves. The content of procyanidin C1 was higher in SR than in SG, while the contents of procyanidin B1 and procyanidin B3 were higher in SR-1 and SR-3 but lower in SR-2 than in SG-1, SG-2 and SG-3 (Fig. 1D). Moreover, the contents of eight flavonoids were found to significantly decrease in the SG-1 *vs.* SR-1 comparison, with two decreased and one increased in the second period, and two decreased and three increased in the third period (Fig. S5). In summary, these patterns of pigment accumulation were consistent with the strikingly different leaf color phenotypes of SR and SG.

### Differentially expressed genes between green- and red-leaved walnuts

RNA sequencing (RNA-Seq) was used to profile genome-wide gene expression and transcriptome changes during leaf development. With three biological replicates, transcriptome sequencing of the 18 walnut leaf samples yielded a total of 134.01 Gb of clean data with Q30 status for 95.22% of bases (Table S5). Furthermore, 92.23%–95.27% of the total clean reads were unique matches to the walnut reference genome, and 4,131 novel genes were identified, including 3,154 annotated genes (Zhao et al., 2021a).

**Figure 2:**
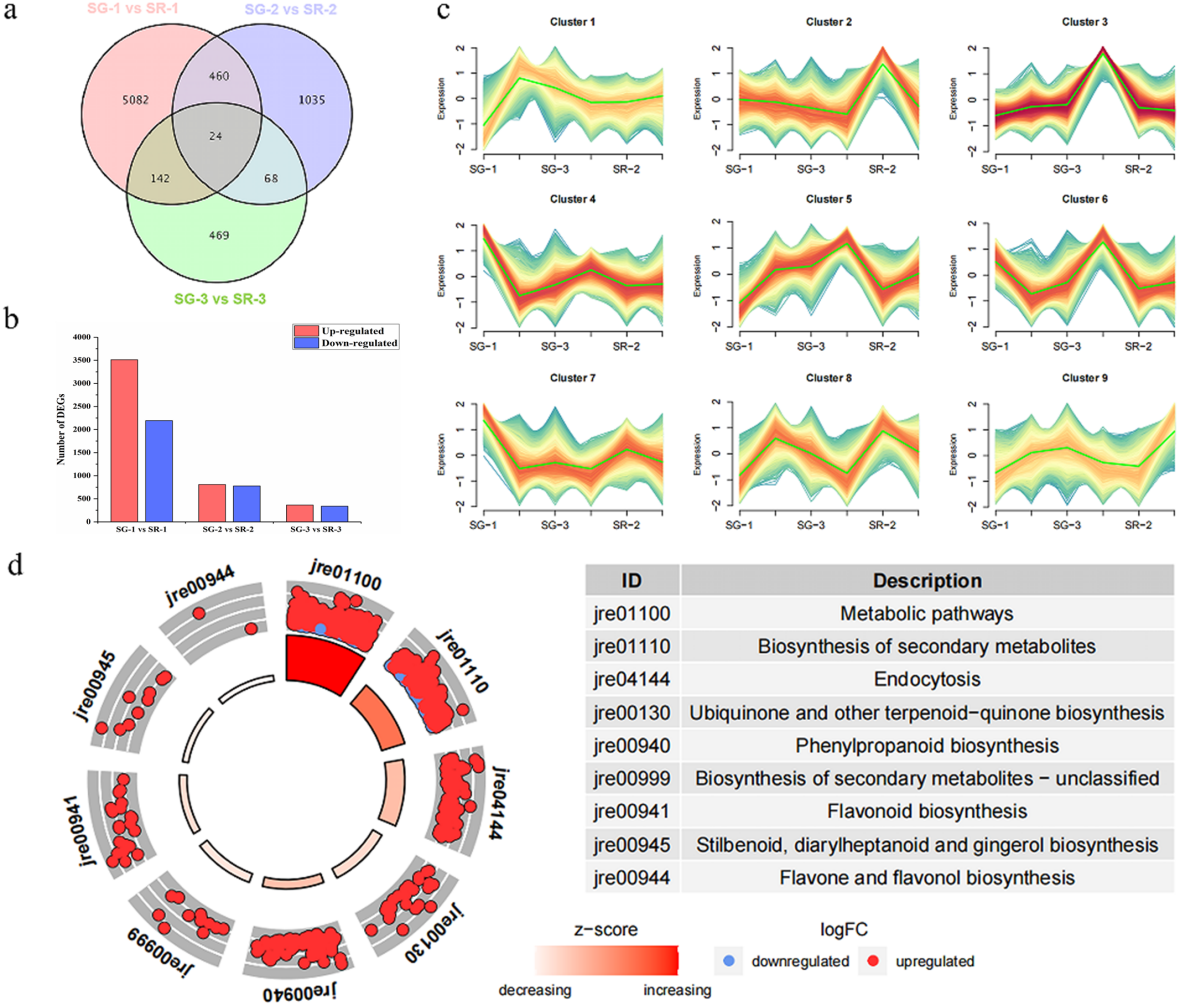
Number, expression trend and KEGG enrichment profiles of differentially expressed genes (DEGs) identified by RNA-seq analysis. (A) Venn diagram depicting the DEGs in the three comparisons of leaf samples. (B) Number of DEGs in the SG-1 *vs.* SR-1, SG-2 *vs.* SR-2 and SG-3 *vs.* SR-3 comparisons. (C) Cluster analysis of expression trend of DEGs. (D) KEGG enrichment profiles of DEGs in the SG-1 *vs.* SR-1, red dots indicate up-regulated DEGs, blue dots indicate down-regulated DEGs.

**Figure 3:**
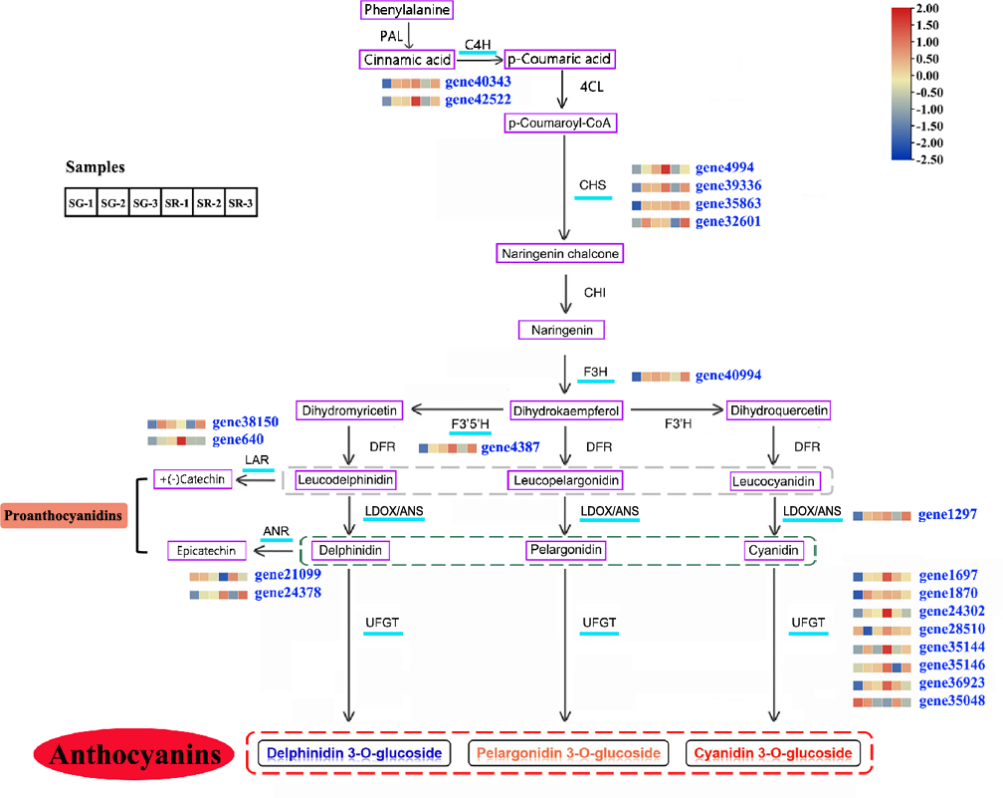
Modulation of anthocyanin and PA biosynthesis pathway genes during walnut leaf development. Structural genes with an available expression profile are underlined in light blue. The heatmap represents the expression of corresponding structural genes in SR and SG, and blue to red in the heatmap indicates the expression levels of structural genes (FPKM value) ranging from low to high. PAL, phenylalanine ammonia lyase; C4H, cinnamate 4-hydroxylase; 4CL, 4-coumarate:CoA ligase; CHS, chalcone synthase; CHI, chalcone isomerase; F3H, flavanone 3-hydroxylase; F3′H, flavonoid 3′-hydroxylase; F3′5′H, flavonoid 3′,5′-hydroxylase; DFR, dihydroflavonol 4-reductase; LDOX, leucoanthocyanidin dioxygenase; ANS, anthocyanidin synthetase; UFGT, UDP-glucose:flavonoid 3-O-glucosyltransferase; LAR, leucoanthocyanidin reductase; ANR, anthocyanidin reductase. Gray dotted line, three types of leucoanthocyanidin as the substrate of LAR; green dotted line, three types of anthocyanidin as the substrate of ANR; red dotted line, three types of anthocyanin as the product of UFGT.

As shown in Fig. 2A, 5,708, 1,587 and 703 DEGs were obtained for the SG-1 *vs.* SR-1, SG-2 *vs.* SR-2, and SG-3 *vs.* SR-3 comparisons, respectively, including 24 genes that were detected in all three comparisons (Fig. 2A). According to the results of transcriptomic analysis, there were 5,708 DEGs between SG-1 and SR-1, of which 3,513 were upregulated and 2,195 were downregulated in expression (Fig. 2B). There were 1,587 DEGs between SG-2 and SR-2, including 810 with upregulated expression and 777 with downregulated expression (Fig. 2B). There were 703 DEGs between SG-3 and SR-3, including 364 with upregulated expression and 339 with downregulated expression (Fig. 2B). According to the phenotypes of different samples at various developmental stages, the DEGs of all samples were clustered based on expression level and divided into nine groups. It was found that the trends of Cluster 3 and Cluster 5 were consistent with the anthocyanin content (Fig. 2C). Then, KEGG enrichment analysis was performed on the DEGs in Cluster 3 and 5. It was found that metabolic pathways such as metabolism, biosynthesis of secondary metabolites, phenylpropanoid biosynthesis and flavonoid biosynthesis were significantly enriched, and most DEGs were significantly higher in SR-1 than SG-1 (Fig. 2D). Further, 17 DEGs were involved in the anthocyanin biosynthesis pathway, four DEGs were involved in the PA biosynthesis pathway, and all of the DEGs were identified in SG-1 and SR-1. Therefore, DEG profiles along with metabolic evidence indicate that these genes are responsible for the increased red color of SR leaves (Fig. 3).

### Analysis of structural genes involved in anthocyanin and PA biosynthesis

On the basis of a reported anthocyanin biosynthesis pathway, a pathway diagram that included the expression heatmap of each structural gene in the anthocyanin biosynthesis pathway of walnut was constructed (Fig. 3). In view of leaf color differences appearing at Stage 1, the combined analysis of transcriptomic and metabolomic data explained the anthocyanin biosynthesis pathway in SG and SR walnuts. Structural genes, including *C4H* genes (gene40343 and gene42522), *CHS* genes (gene4994, gene39336, gene35863 and gene32601) (Zhao et al., 2021b), *F3H* (gene40994), *F3′5′H* (gene4387), *LDOX* (gene1297) and *UFGT* genes (gene28510, gene35146, gene1870, gene24302, gene35144, gene1697, and gene36923), were all upregulated in expression, and only one *UFGT* gene (gene35048) was downregulated in expression in the SG-1 *vs.* SR-1 comparison (Fig. 3). In the biosynthetic pathways of PAs, LAR and ANS, ANR and UFGT compete for substrates, and two *LAR* genes (gene38150 and gene640) and one *ANR* gene (gene24378) were upregulated in expression while one *ANR* gene (gene21099) was downregulated in expression in the SG-1 *vs.* SR-1 comparison. *C4H* genes (gene40343 and gene42522), *CHS* genes (gene4994 and gene39336) and *F3H* (gene40994) were downregulated in expression in the SG-2 *vs.* SR-2 comparison but showed no significant change in the SG-3 *vs.* SR-3 comparison; *CHS* (gene35863) and *UFGT* genes (gene1870, gene24302, gene35048, and gene36923) showed no significant change in the SG-2 *vs.* SR-2 and SG-3 *vs.* SR-3 comparisons; *CHS* (gene32601), *F3′5′H* (gene4387), *LDOX* (gene1297), *UFGT* genes (gene35144, and gene35146), *LAR* (gene38510) and *ANR* (gene24378) were downregulated in expression in the SG-2 *vs.* SR-2 comparison but were upregulated in expression in the SG-3 *vs.* SR-3 comparison; *UFGT* genes (gene1697, and gene28510) and *ANR* (gene21099) were upregulated in expression in the SG-2 *vs.* SR-2 comparison but showed no significant change in the SG-3 *vs.* SR-3 comparison; *LAR* (gene24378) was downregulated in expression in the SG-3 *vs.* SR-3 comparison but no significant change in the SG-2 *vs.* SR-2 comparison; and *PAL*, *4CL*, *CHI*, *DFR*, and *F3′H* showed similar patterns in SG and SR.

### Identification of TFs related to anthocyanin and PA biosynthesis

**Figure 4:**
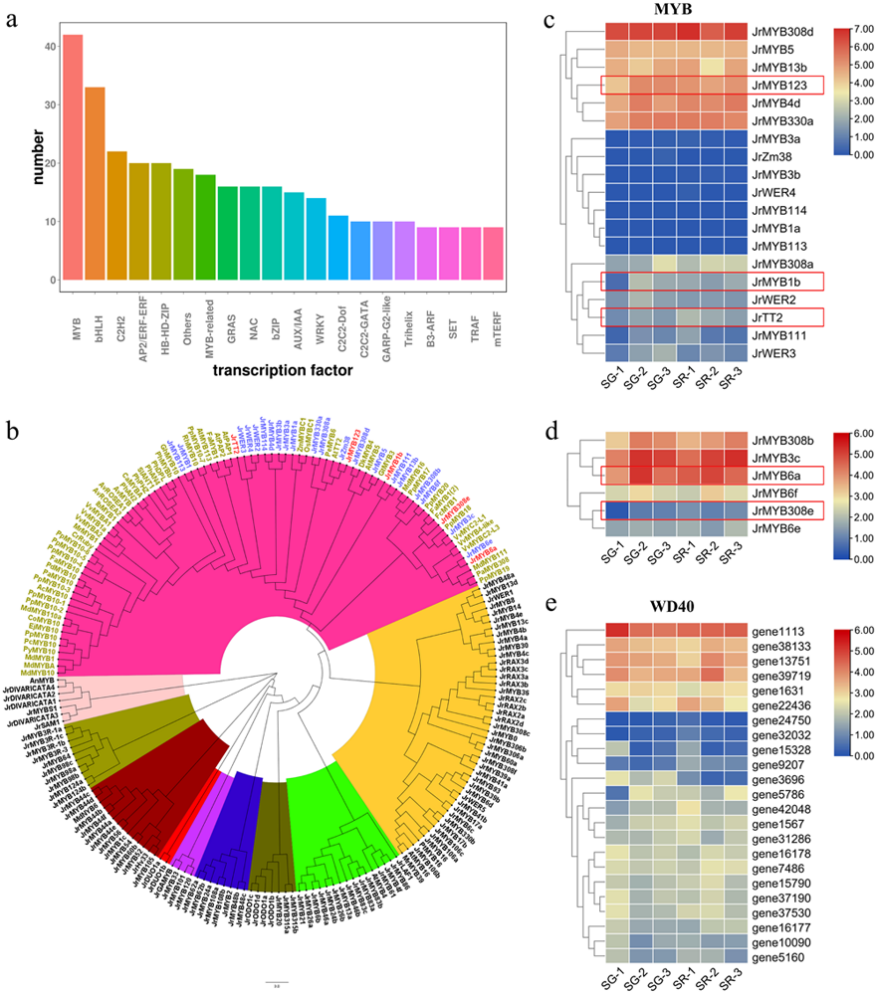
Analysis of transcription factors related to anthocyanin biosynthesis. (A) Number of differentially expressed transcription factors in overall transcriptome data. (B) Phylogenetic tree of MYB proteins in walnut and *Arabidopsis thaliana* and MYBs related to anthocyanin biosynthesis in other species. (C) Expression heatmap of *Jr MYBs* highly homologous to anthocyanin-related MYBs of other species, the genes in red flames were selected based on a false discovery rate (FDR) < 0.05 and an — Log2FC — ≥ 1.5. (D) Expression heatmap of *Jr MYBs* highly homologous to MYB repressors in anthocyanin biosynthesis of other species. (E) Expression heatmap of differentially expressed WD40s in SG and SR. The scale bars represent the log2 transformations of the FPKM values.

Anthocyanin and PA biosynthesis are regulated by the MBW (MYB-bHLH-WD40) complex. As shown in Fig. 4A, MYB and bHLH were the top two TFs related to anthocyanin biosynthesis in walnut leaves. Based on the *Arabidopsis* MYB protein domains, 135 putative walnut MYB protein sequences were obtained with default parameters using HMMER and BLASTP (Table S6). A phylogenetic tree was constructed using 135 JrMYBs and MYBs of other species related to anthocyanin and PA biosynthesis (Fig. 4B). Nine subfamilies and 25 JrMYBs were obtained in the current study. Based on a false discovery rate (FDR) <0.05 and an — Log_2_FC — ≥ 1.5, two differentially expressed *MYB* genes related to anthocyanin biosynthesis in red frames, namely, *JrMYB1b (gene38312)*, and *JrMYB123 (gene9445)*, and one *MYB* gene related to PA biosynthesis, *JrTT2 (gene39085),* were screened from 19 MYBs with high similarity to anthocyanin biosynthesis-related MYBs in other species (Fig. 4C). The other six MYBs showed high similarity to the MYBs that negatively regulated anthocyanin biosynthesis; *JrMYB308e (gene29715)* and *JrMYB6a (gene32351)* were upregulated in the SG-1 *vs.* SR-1 comparison (Fig. 4D). The bHLHs involved in anthocyanin biosynthesis, including *JrbHLHA1*, *JrbHLHA2*, *JrEGL1a*, and *JrEGL1b*, have been reported by our research group (Zhao et al., 2021a). For WD40 (Fig. 4E), we obtained 23 DEGs, including 5 with upregulated expression (*gene1567*, *gene24750*, *gene32032, gene42048*, and *gene5786*) and 11 with downregulated expression (*gene10090*, *gene1113*, *gene13751*, *gene15328*, *gene16177*, *gene16178*, *gene1631*, *gene31286*, *gene3696*, *gene37190*, and *gene38133*) in the first stage, eight with upregulated expression (*gene15790*, *gene22436*, *gene37190*, *gene37530*, *gene39719*, *gene5160*, *gene7486*, and *gene9207*) and one with downregulated expression (*gene3696*) in the second stage, and one with downregulated expression (*gene3696*) in the third stage. Among them, *gene3696* showed a trend of downregulated expression in all three stages. These TFs may affect or participate in the expression regulation of structural and regulatory genes involved in anthocyanin and PA biosynthesis.

### Correlation analysis between selected transcripts and Anthocyanins/PAs

**Figure 5:**
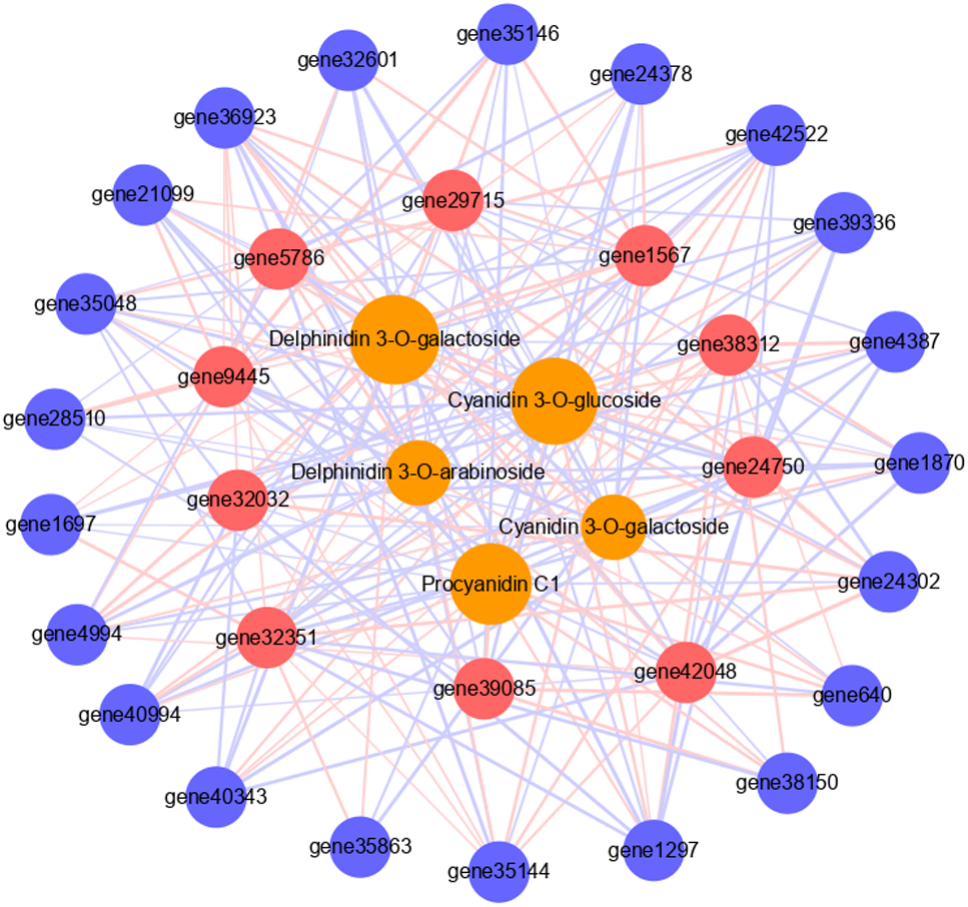
Connection network between core genes and anthocyanin and PA metabolites. The orange circles indicate metabolites, red circles indicate structural genes, blue circles indicate transcription factors. The size of the orange circles indicates the number of genes associated with metabolites. The red lines indicate positive correlation, the blue lines indicate negative correlation, and the thickness of the line indicates the size of correlation.

To further verify the role of candidate genes in anthocyanins and PAs accumulation, we conducted correlation analyses between selected transcripts and key metabolites (Fig. 5, Tables S7, S8). The significant correlation (correlation coefficient, R^2^ >0.8) between a total of 31 transcripts and five metabolites was detected, including 21 structural genes, 10 TFs, four anthocyanins (delphinidin 3-*O*-galactoside, cyanidin 3-*O*-galactoside, delphinidin 3-*O*-arabinoside and cyanidin 3-*O*-glucoside), and 1 PA (procyanidin C1). Each metabolite was correlated with many different transcripts. Cyanidin 3-O-galactoside and delphinidin 3-O-arabinoside were correlated with the fewest transcripts, both 17, and there were five and six positive correlations, 12 and 11 negative correlations respectively. Delphinidin 3-O-galactoside was correlated with the highest number of transcripts: 26 transcripts, there were 15 positive correlations and 11 negative correlations. Among them, six TFs had significant positive correlation with it, including two MYBs and four WD40s. Procyanidin C1 was correlated with 23 transcripts, among which the MYB transcription factor JrTT2 (*gene39085*) related to PA synthesis had a significant positive correlation. In addition, 10 TFs were related to different structural genes. Among them, MYB6a (*gene32351*) was significantly correlated with the most structural genes (16), and JrTT2 (*gene39085*) was correlated with the least structural genes (seven). The results showed that the expression of structural genes regulated by TFs was strongly correlated with the increase of anthocyanin and PA levels in red walnut.

### qPCR analysis of DEGs related to anthocyanin and PA biosynthesis

Based on our results reported above, 12 differentially expressed structural genes involved in the anthocyanin and PA biosynthetic pathway and four *MYB* genes were analyzed using qPCR methods. The results in Fig. S6 showed that eight structural genes, namely, two *JrC4Hs* (gene40343 and gene42522), *JrF3′5′H* (gene4387), and five *JrUFGTs* (gene35144, gene35146, gene1697, gene24302 and gene1870), and four *MYB* genes (gene38312, gene32351, gene9445, and gene39085) were highly expressed in red-leaved walnut at the first stage, which indicated that the high expression of genes involved in anthocyanin biosynthesis was related to the red color of walnut leaves. This result also further suggested that the transcriptome data were accurate and consistent with the expression of genes related to anthocyanin and PA biosynthesis in red walnut.

### Amplification and sequence alignment analysis of DEGs related to anthocyanin and PA biosynthesis

In order to further obtain the cause of pigmentation on the genomic level, the CDS sequences of 21 structural genes and five TFs related to anthocyanin and PA biosynthesis were amplified and analyzed (Fig. S7). The results showed that there were some base differences in the nucleotide sequences of nine structural genes (*C4H*-gene40343, *CHS*-gene39336, *F3′5′H*-gene4387, *LDOX*-gene1297, *LAR*-gene38150, *ANR*-gene24378, *UFGT*-gene1697, *UFGT*-gene1870, *UFGT*-gene35144) and one MYB (MYB1b-gene38312) in the natural hybrid progenies of red walnut with different phenotypes (red leaves and green leaves). Among them, there was only one base difference in the sequences of *C4H*-gene40343, *UFGT*-gene1870 and *MYB1b*-gene38312, while there were more than two base differences in other genes. However, *F3′5′H*-gene4387, *LAR*-gene38150 and *ANR*-gene24378 even had deletion mutations. In addition, we further amplified the promoter regions (the 2,000-bp sequence upstream of the transcription start site) of all the above genes by using the genomic DNA of different samples. The results showed that there was no difference in all promoter sequences between the two color leaves.

## Discussion

The primary pigments in red walnut have been identified as flavonoids, particularly anthocyanins (Li et al., 2018; Zhao et al., 2021b), but previous studies on walnut coloration are limited. The current work aimed to more comprehensively explore the metabolites involved in the color changes in walnut leaves, and 395 metabolites were obtained from walnut leaves using widely targeted metabolomic profiling and anthocyanin detection (Tables S3, S4). This was the first study to present a genome-wide examination of anthocyanins and the gene expression profiles of walnuts, aiming to provide a more complete landscape of the metabolites involved in the color changes in walnut leaves during development. According to the metabolic data, the contents of 26 anthocyanins increased in SR-1 compared with SG-1, while the contents of eight flavonoids decreased in SR-1 compared with SG-1. Following walnut leaf development, the contents of only two flavonoids had decreased in the SG-2 *vs.* SR-2 and SG-3 *vs.* SR-3 comparisons, while those of one and three flavonoids had increased, respectively. Therefore, the variation in anthocyanin contents across development may show the opposite pattern of that in flavonoid contents. Moreover, we also found that PA accumulation was higher in the first and third stages in the SG *vs.* SR comparison (Fig. 1D). By transcriptomic and metabolomic analysis, 21 putative genes were predicted to be involved in anthocyanin and PA biosynthesis (Fig. 3). According to the results of metabolomic and transcriptomic analysis, many more DAMs and DEGs were detected in the SG-1 *vs.* SR-1 comparison than in the others, indicating that the biosynthesis of anthocyanin may also influence other functions in red walnut. These findings provide a theoretical basis for further studies on the mechanism of color formation in red walnut.

Anthocyanins are secondary metabolites in plants, such as ornamental plants, fruits, vegetables and medical plants, and they play various roles in many biological processes, including determining fruit quality and flower colors, improving resistance, and helping plants avoid UV and strong light damage (Petroni & Tonelli, 2011; Naing & Kim, 2018). In addition to producing highly nutritious kernels, walnuts are also ecologically important trees. The red walnut accession RW-1 possesses great ornamental value, nutritional value and economic benefits. A total of 26 anthocyanins were detected in green- and red-leaved walnuts (Fig. 1D), and cyanidin 3-*O*-glucoside, cyanidin 3-*O*-galactoside, delphinidin 3-*O*-galactoside, and delphinidin 3-O-arabinoside were the main anthocyanins in SR. Importantly, the variation in anthocyanin content was consistent with the observed leaf color changes. Interestingly, six anthocyanidin compounds, namely, Cy, Dp, Mv, Pg, Pn, and Pt, were found not only in SR but also in SG (Fig. 2). Similar results were also reported for white *Salvia miltiorrhiza* flowers (Jiang et al., 2020). The six anthocyanidins accumulated less in green-leaved walnuts than in red-leaved walnuts, suggesting that complete anthocyanin metabolic pathways were also present in SG. The color depended on the types and contents of anthocyanins, co-pigments, chlorophyll, vacuolar pH, and metal ions (Tanaka, Sasaki & Ohmiya, 2008). The other factors affecting the color of walnut leaves need to be studied further.

The core genes for walnut anthocyanin and PA biosynthesis involve multiple enzymes encoded by structural genes (*C4H*, *CHS*, *CHI*, *F3H*, *F3′H*, *F3′5′H*, *DFR*, *LDOX*, *UFGT*, *LAR* and *ANR*) (Tanaka, Sasaki & Ohmiya, 2008). The results from this study showed that the expression levels of *C4Hs*, *CHSs*, *F3H* and *F3′5′H* in SR-1 were significantly higher than those in SG-1 (Fig. 3, Fig. S6). These genes are common to both the anthocyanin and PA biosynthesis pathways. The expression of *LDOX* and *UFGT* genes in SR-1 was significantly higher than that in SG-1 (Fig. 3, Fig. S6), suggesting that the genes mainly regulated red coloration in red-leaved walnuts. As walnut leaves developed, the color tended to become green following anthocyanidin degradation, and these structural genes were downregulated in expression in the SG-2 *vs.* SR-2 comparison. In the third stage, the SR leaf color remained stable, with only the veins appearing red, and the expression levels of these structural genes remained stable and were higher in SR-3 than in SG-3.

The *UFGT* gene is involved in the final steps of the flavonoid biosynthesis pathway (biosynthesis and accumulation of anthocyanins). The expression of *UFGT* genes (gene35144, gene35146, gene1697, gene24302 and gene1870) was higher in SR-1 than in SG-1, and the expression of the *UFGT* gene (gene35038) showed the opposite trend. Notably, the trends of expression variation in three *UFGT* genes during the three stages corresponded to the color change in walnut leaves. Anthocyanins are extremely unstable and easily degraded; therefore, glycosylation is important for stabilization. UFGTs, involved in the last step of anthocyanin biosynthesis, are considered to be the key enzymes controlling anthocyanin biosynthesis in many plants, and they play an important role in anthocyanin metabolism (Caputi et al., 2012; Liu et al., 2019; Koes, Verweij & Quattrocchio, 2005; Montefiori et al., 2011). The present study first explored the expression profile of *UFGTs* using red walnuts, providing new insights for studying *UFGT* gene function. In relation to PA biosynthesis, the expression of *LAR* (gene38150) and *ANR* (gene24378) was also higher in SR-1 and SR-3 than in SG-1 and SG-3 but lower in SR-2 than in SG-2, suggesting that these genes play important roles in PA biosynthesis (Fig. 3, Fig. S6). Amplification and sequence analysis of candidate genes showed that there were base deletion mutations in *F3′5′H*-gene4387, *LAR*-gene38150 and *ANR*-gene24378. It is speculated that they may play an important role in the accumulation of anthocyanins and PAs in the process of pigmentation (Fig. S7). Further promoter sequence amplification analysis of candidate transcription factors and structural genes showed that they all had no difference in the two color samples. Therefore, the alteration of the properties of regulatory genes binding or regulatory structural genes may be caused by the variation of their own coding sequence rather than the difference of promoter sequence.

Anthocyanin biosynthesis is regulated by the MBW (MYB-bHLH-WD40) complex. In this complex, bHLH and MYB TFs have DNA binding functions and can specifically bind to the promoters of structural genes in the anthocyanin and PA biosynthesis pathway, while the WD40 protein plays a stabilization role in the MBW complex (Hichri et al., 2011; van Nocker & Ludwig, 2003). In *Actinidia chinensis* Planch, AcMYB123 and AcbHLH42 promote anthocyanin accumulation in the inner pericarp of by activating the promoters of *AcANS* and *AcF3GT1* (Wang et al., 2019); PyWRKY26 and PybHLH3 act together on the *PyMYB114* promoter, and co-transfection can activate the expression of *PyUFGT* to regulate the accumulation and transport of anthocyanin in pear (Li et al., 2020a; Li et al., 2020b; Li et al., 2020c). There are also many MYB repressors that act to reduce anthocyanin production. In this study, we found two *MYB* expression patterns that showed positive correlations with anthocyanin biosynthesis, which may provide feedback regulation in walnut leaves like FaMYB1, PtrMYB57 and TrMYB133 (Wang et al., 2017). In this study, we identified three *MYBs* predicted as the activators of anthocyanin and PA biosynthesis, two *MYBs* predicted as the repressors of anthocyanin biosynthesis, four bHLHs and five WD40s based on their expression levels and phylogenetic analysis, suggesting that these TFs contributed to the expression of genes involved in anthocyanin synthesis. Further, we analyzed the correlation among candidate TFs, structural genes and key metabolites. The results showed that each TF had a strong correlation with all metabolites and different numbers of structural genes, indicating that transcription factor complexes can regulate the expression of structural genes to affect the biosynthesis of anthocyanins and PAs metabolites (Fig. 5). However, the difference of gene coding sequences altered the binding or regulation of regulatory factors to structural genes in different color leaves, resulting in the effective increase of anthocyanins and PAs accumulation in red walnut, and finally promoted the production of red mutation (Fig. S7).

These findings can help elucidate the molecular mechanism and regulatory networks of anthocyanin biosynthesis in walnut and provide a biological basis for breeding new walnut cultivars.

## Conclusions

In this study, metabolomics and transcriptomics were used to reveal the anthocyanin biosynthesis metabolic pathway. Transcriptome analysis demonstrated that the expression of structural genes (*C4H*, *F3H*, *F3′5′H* and *UFGTs*), three *MYBs* predicted as the activators of anthocyanin and PA biosynthesis, two *MYBs* predicted as the repressors of anthocyanin biosynthesis, and five *WD40s* in the anthocyanin biosynthetic pathway was significantly higher in SR walnuts. Delphinidin 3-*O*-galactoside, cyanidin 3-*O*-galactoside, delphinidin 3-*O*-arabinoside, cyanidin 3-*O*-glucoside and procyanidin C1 were identified as the primary components of anthocyanins and PA because of their higher contents. Gene-metabolite correlation analyses revealed a core set of 31 genes that were strongly correlated with four anthocyanin and one PA metabolites. The alteration of gene coding sequences altered the binding or regulation of regulatory factors to structural genes in different color leaves, resulting in the effective increase of anthocyanins and PAs accumulation in red walnut. Our results provide valuable information on anthocyanin and PA metabolites and candidate genes for anthocyanin and PA biosynthesis, yielding new insights into anthocyanin and PA biosynthesis in walnuts.

##  Supplemental Information

10.7717/peerj.14262/supp-1 Supplemental Information 1 Supplemental Tables

10.7717/peerj.14262/supp-2 Supplemental Information 2 Supplemental Figures

10.7717/peerj.14262/supp-3 Supplemental Information 3 Raw data

## Abbreviations

ANR: anthocyanidin reductase
ANS: anthocyanidin synthetase
C4H: cinnamate 4-hydroxylase
CHI: chalcone isoenzyme
CHS: chalcone synthetase
Cy: cyanidin
DAMs: differentially accumulated metabolites
DEGs: differentially expressed genes
DFR: dihydroflavonol 4-reductase
Dp: delphinidin
F3H: flavanone 3-hydroxylase
F3′5′H: flavonoid 3′,5′-hydroxylase
FDR: false discovery rate
FPKM: fragments per kilobase of transcript per million fragments mapped
GO: Gene Ontology
KEGG: Kyoto Encyclopedia of Genes and Genomes
LAR: leucoanthocyanidin reductase
LDOX: leucoanthocyanidin dioxygenase
LC–MS/MS: liquid chromatography/tandem mass spectrometry
Mv: malvidin
PA: proanthocyanidin
PAL: phenylalanine ammonia lyase
PCA: principal component analysis
Pg: pelargonidin
Pn: peonidin
Pt: petunidin
QC: quality control
RW-1: red walnut accession ‘RW-1’
SG: green-leaved walnuts among RW-1 natural hybrid progenies
SR: red-leaved walnuts among RW-1 natural hybrid progenies
UFGT: UDP glucose:flavonoid-3-O-glucosyltransferase
UPLC–ESI–MS/MS: ultra-performance liquid chromatography coupled with electrospray ionization/tandem mass spectrometry

## Additional Information and Declarations

### Data Availability

The following information was supplied regarding data availability: The transcriptome data is available at NCBI: PRJNA688391. The metabolome data is available at Figshare: Wang, Lei (2022): MTBLS3548.zip. figshare. Journal contribution. https://doi.org/10.6084/m9.figshare.21201761.v1.
