# Supplementary material for: Integrated metabolomic and transcriptomic analysis of the anthocyanin and proanthocyanidin regulatory networks in red walnut natural hybrid progeny leaves
Source: PeerJ. 2022 Oct 20;10:e14262. doi: 10.7717/peerj.14262 (PMC9588303; doi:10.7717/peerj.14262)
Supplement: Supplemental Information 1 [file peerj-10-14262-s001.docx]

**Table S1.** List of primers used for qRT-PCR.

| Primer name | Oligonucleotide Primers |
| --- | --- |
| qRT-JrC4H(gene40343)-F  qRT-JrC4H(gene40343)-R  qRT-JrC4H(gene42522)-F  qRT-JrC4H(gene42522)-R  qRT-JrF3H(gene40994)-F  qRT-JrF3H(gene40994)-R  qRT-JrF3’5’H(gene4387)-F  qRT-JrF3’5’H(gene4387)-R  qRT-JrLDOX/ANS(gene1297)-F  qRT-Jr LDOX/ANS(gene1297)-R  qRT-JrUFGT(gene1870)-F  qRT-JrUFGT(gene1870)-R  qRT-JrUFGT(gene24302)-F  qRT-JrUFGT(gene24302)-R  qRT-JrUFGT(gene35144)-F  qRT-JrUFGT(gene35144)-R  qRT-JrUFGT(gene1697)-F  qRT-JrUFGT(gene1697)-R  qRT-JrUFGT(gene25810)-F  qRT-JrUFGT(gene25810)-R  qRT-JrUFGT(gene34146)-F  qRT-JrUFGT(gene34146)-R  qRT-JrUFGT(gene35048)-F  qRT-JrUFGT(gene35048)-R  qRT-JrANR(gene21099)-F  qRT-JrANR(gene21099)-R  qRT-JrANR(gene24378)-F  qRT-JrANR(gene24378)-R  qRT-JrLAR(gene38150)-F  qRT-JrLAR(gene38150)-R  qRT-JrLAR(gene640)-F  qRT-JrLAR(gene640)-R  qRT-JrMYB1b(gene38312)-F  qRT-JrMYB1b(gene38312)-R  qRT-JrMYB6a(gene32351)-F  qRT-JrMYB6a(gene32351)-R  qRT-JrMYB123(gene9445)-F  qRT-JrMYB123(gene9445)-R  qRT-JrMYBTT2(gene39085)-F  qRT-JrMYBTT2(gene39085)-R  qRT-Jr18s-F  qRT-Jr18s-R | CAAAGGCGGAAGGCTTCAAA  GGACCGTATCAAGCTCGTGT  CTGTCGAGCACAAAGGCAAC  TGCAAGCCCCCACTCAATAG  GAGACTATTCGAGGTGGCCG  AATGTGAGGTCTGGCTGTGG  GTTGGTCAAGCTCAGCCTCT  TGAAGAAAGCTGCCGTGGAT  TGAAGAAAGCTGCCGTGGAT  GTCCTCAGGGTAGACGAGGT  GCGGTTCTCCTTCCTCAACA  GCGGCAACGAATAATGGGAC  GAAGCGAGTTGCGTTCCTT  AGCCTTATCCTTATCAATCCC  TGCGATAAGGGCGAGGGTGAAG  CTCAGTTTCGGTCGGTCGTTGC  TGGCATTTCCATTTGGCTGC  TGTGCGTGCGAAAATTGATGA  CTGCTCCTGACGTTCGGTTC  CACATGACCCACCGGTATGC  CCCTTGGATCCCCTGCTACA  CGACGAAGACTGTGACGGTG  GCACGGCTGTATCCAACCACTC  GGCTCAATCGGATTCTCGGTAACC  TGCCCTAGTGGGAGGATATCAA  TGATGGTTGGATTGGCAGCA  GGAAAGAAAACTGCGTGCGT  TACCCCTAGGATTGCTGGCT  GTGAGAAGGTGGACAGAGGC  ATCGTTGGCAGCGGAAAGTA  CCTATGTTCTCGTCCGTCCG  ACCAAACGCCTCACTCTCTG  AGAGTGAGATTTCACACCGAAGA  TGGTGCCCTTCCCATCTCTA  CTCGCCAAAGCGCCTCT  CGTGGAGGAGCTCAGACTTC  TAGCAGCAGCACTACCTGTG  ACTCAGTCAGATCAGGAGCCA  TGATCATTCCGAGCCCAGTG  TTTGTGAACGTGCTTTCGGC  ATTGGTTGCGGATCAGGACT  GCTCCAATGCAACATCAAGC |

**Table S2.** List of primers used for gene amplification.

| Primer name | Oligonucleotide Primers |
| --- | --- |
| RT-JrC4H(gene40343)-F  RT-JrC4H(gene40343)-R  RT-JrC4H(gene42522)-F  RT-JrC4H(gene42522)-R  RT-JrF3H(gene40994)-F  RT-JrF3H(gene40994)-R  RT-JrCHS(gene32061)-F  RT-JrCHS(gene32061)-R  RT-JrCHS(gene39336)-F  RT-JrCHS(gene39336-R  RT-JrCHS(gene4994)-F  RT-JrCHS(gene4994)-R  RT-JrCHS(gene35863)-F  RT-JrCHS(gene35863)-R  RT-JrF3’5’H(gene4387)-F  RT-JrF3’5’H(gene4387)-R  RT-JrLDOX/ANS(gene1297)-F  RT-Jr LDOX/ANS(gene1297)-R  RT-JrUFGT(gene1870)-F  RT-JrUFGT(gene1870)-R  RT-JrUFGT(gene24302)-F  RT-JrUFGT(gene24302)-R  RT-JrUFGT(gene35144)-F  RT-JrUFGT(gene35144)-R  RT-JrUFGT(gene1697)-F  RT-JrUFGT(gene1697)-R  RT-JrUFGT(gene25810)-F  RT-JrUFGT(gene25810)-R  RT-JrUFGT(gene34146)-F  RT-JrUFGT(gene34146)-R  RT-JrUFGT(gene35048)-F  RT-JrUFGT(gene35048)-R  RT-JrUFGT(gene36923)-F  RT-JrUFGT(gene36923)-R  RT-JrANR(gene21099)-F  RT-JrANR(gene21099)-R  RT-JrANR(gene24378)-F  RT-JrANR(gene24378)-R  RT-JrLAR(gene38150)-F  RT-JrLAR(gene38150)-R  RT-JrLAR(gene640)-F  RT-JrLAR(gene640)-R  RT-JrMYB1b(gene38312)-F  RT-JrMYB1b(gene38312)-R  RT-JrMYB6a(gene32351)-F  RT-JrMYB6a(gene32351)-R  RT-JrMYB123(gene9445)-F  RT-JrMYB123(gene9445)-R  RT-JrMYB308e(gene29715)-F  RT-JrMYB308e(gene29715)-R  RT-JrMYBTT2(gene39085)-F  RT-JrMYBTT2(gene39085)-R | ATGGATCTCCTCCTGGTGGA  TCAAAACGATCTTGGCTTTAAAAC  ATGGATATCCTCCTCTTGGAGAA  TTAAGATGATCTTGGCTTAGCAAC  ATGGCCCCAACTACTCTCAC  TTAAGCAAAGATCTCCTCAATAGG  ATGGTGACAGTGGAAGATGTCCG  AGCAGACAAACTATGCAGCACAAC  ATGGTGACAGTGGAAGATGTTCG  AGCAGAGACACTGTGGAGCACG  ATGGCATCTGTGGAAGAATTCCT  GTGAATCGTCTCTACTGGGACACTA  ATGGCGTCCATGGAGGA  GATATTGACACTGTGCAGCA  ATGGCCCTAGACATATTTCTTCTC  TCAAGAAGCATATGCACTCAAAG  ATGGTGACTTCAGTTTTACCAAGAG  TCATTTCAAGGCAAGAGAATCC  ATGAGTCAAAGCTCAGGAAAAAATC  TTATTGAGAGATTAGGTCAAGCAAAGT  ATGAGTCAAAGCTCCGGAAAAAAT  CTAATTATTTTGAATTTTGGTTGTTACGG  ATGGAGATCTCAAAGCCACATGCAGC  TCAGTTGGGCTTTAGTGCTAAACTCG  ATGGATATGGTAGTGGCCGAG  CTACAAGCTTTTAAGATCTCTGATCTTC  ATGTCGCTCACAGAAAAGCC  TTAAGACGCAGATATCAACTCCAC  ATGGAGATCTCAAAGCCCCATGCAG  CTAATCTGTTATCTGCTGTGCCAGCC  ATGCATGACTGTCACCGCAC  TTACTTGGTGATTAACTCAACCAGAG  ATGAGTCAAAGCGCAGGAAAAAATCAGC  TTATTGAGAGATTAGCTCAAGC  ATGGAGAACAGTTACAGTAGAGTCTGC  TTAGATTAGAGCTCCAAATCGCTCT  ATGGCCACCCAACATATCG  TTAGTTCTTCAGCTCGCCCT  ATGTGTGGGTCAAACAGTACTTCTTC  TCATGCTGCAGTAATAGCCAATG  ATGACTATGACACGGCTGCC  TCAAGCACATGTTGCAGTGATT  ATGGGAAGGGCACCATGTT  CTATTTTTCTTCAGTACTACCTTTGTTG  ATGAGAAAACCTTGCTGTGATAAAG  CTAAAAAAGGATGAGGGTAGGAG  ATGGGAAGGAGTCCTCGTTG  TTATTCCAAATTGCATTTTGAA  ATGAGGAAACCTTGCTGTGACA  CTATGCTTTGCTCTCTTCCACAC  ATGGAAGTTAAAAGAGTGAAGCC  TCACTTACCTACCCTTTCACCAT |

**Table S3.** List and characteristics of the metabolites detected in walnut leaf samples.

| Index | Compounds | Class | Rt (min) |
| --- | --- | --- | --- |
| p0-0227.0737-006.82-splash10-0006-0900000000-6b0d58466ecec298fbee | (Z)-Resveratrol | Alcohols | 6.61 |
| p1-0179.1051-006.61-splash10-003u-3900000000-61e67b73a9518d19833a | Methylisoeugenol | Alcohols | 6.74 |
| p1-0316.2820-011.33-splash10-03di-9240000000-f1bcbe09f5ddeef3701a | 6-hydroxysphingosine | Alcohols | 11.26 |
| p1-0325.2746-011.56-splash10-00gv-9810000000-c1a05d15db5b5ba27c5f | PGF1α Alcohol | Alcohols | 11.51 |
| p1-0443.3834-013.76-splash10-0006-0220900000-21a94a7a23ef1a84cb29 | Protopanaxadiol | Alcohols | 13.56 |
| p0-0191.0369-008.16-splash10-0002-1900000000-18b47fc8733b21646083 | 4-METHYLDAPHNETIN | Alkaloids | 7.99 |
| p1-0138.0541-000.74-splash10-0006-9200000000-ffa53f2c69a32c7677f2 | Trigonelline | Alkaloids | 0.68 |
| p1-0144.0789-005.52-splash10-0006-3900000000-3eb3fd3e544b06057375 | 1-Naphthylamine | Alkaloids | 5.8 |
| p1-0170.0927-013.07-splash10-0pbc-3900000000-e4f68b538a942618da77 | Diphenylamine | Alkaloids | 12.89 |
| p1-0243.0628-008.37-splash10-0002-0910000000-99bf092984ac1f77fa8d | 6,2'4'-Trihydroxy-2-phenylbenzofuran | Alkaloids | 8.35 |
| p1-0267.1329-003.46-splash10-00di-1910000000-71402a0c64405e67957f | N5-(4-Methoxybenzyl)glutamine | Alkaloids | 3.19 |
| p1-0344.1036-004.26-splash10-03di-0900000000-842f3b9e83ae60eb3249 | DHBOA-Glc | Alkaloids | 4.24 |
| p1-0375.0823-007.40-splash10-0a4i-0009000000-5d20a7c8a48b06ddf151 | Levistolide A | Alkaloids | 7.2 |
| p1-0507.1003-010.31-splash10-0a4r-0002970000-0c51dfb1ee9ebfff0a5a | Cassiamin C | Alkaloids | 10.52 |
| p1-0186.2207-007.58-splash10-001r-3900000000-fc754022107bf3570698 | Tributylamine | Alkaloids | 7.36 |
| p0-0135.0320-000.72-splash10-056r-9000000000-e4d4b441ecc217c7ef3c | Threonic acid | Amino acid and derivatives | 0.68 |
| p0-0156.0680-003.18-splash10-03di-3900000000-5f70d34e44f33240580c | N-ACETYLPROLINE | Amino acid and derivatives | 3.01 |
| p1-0118.0860-001.11-splash10-0a4i-9000000000-295ae17283d8aa227e7b | Valine | Amino acid and derivatives | 1.04 |
| p1-0148.0591-000.66-splash10-001i-9000000000-f49cd05ab08860b1c112 | L-Glutamic acid | Amino acid and derivatives | 0.65 |
| p1-0203.1482-000.76-splash10-00di-9200000000-f52744fa0f79175966f2 | Symmetric dimethylarginine | Amino acid and derivatives | 0.69 |
| p1-0209.0896-002.84-splash10-00kb-2900000000-93c228c39ae5031a880e | Kinurenine | Amino acid and derivatives | 2.63 |
| p1-0320.1091-004.47-splash10-000i-0900000000-fd9d103d98736db6d27d | Gly Glu Asp | Amino acid and derivatives | 4.27 |
| p1-0323.1072-006.90-splash10-0f89-1980000000-8b04c8e23084b23b5643 | Ser Cys Asn | Amino acid and derivatives | 7.01 |
| p1-0613.1580-002.21-splash10-0a59-0259521000-304e7b3baed0031e7f0b | Glutathione (oxidized) | Amino acid and derivatives | 2.67 |
| p1-0176.1014-000.70-splash10-00di-9100000000-138b690bc40814d918d3 | Citrulline | Amino acid and derivatives | 0.65 |
| p0-0203.0846-003.87-splash10-00l6-2910000000-5c8a4bf0f1e610c0964f | D-Tryptophan | Amino acid and derivatives | 3.61 |
| p1-0328.1080-004.81-splash10-0002-0900000000-776c8bcb04613399bb5a | L-Tyrosine, N-[(2E)-3-(4-hydroxyphenyl)-1-oxo-2-propen-1-yl]- | Amino acid and derivatives | 4.59 |
| p1-0166.0848-002.03-splash10-00di-3900000000-98b6d792ba4b847dc95a | L-Phenylalanine | Amino acid and derivatives | 2.08 |
| p1-0175.1083-000.64-splash10-00di-9000000000-a2257afa6298461adfeb | N-Acetylornithine | Amino acid and derivatives | 0.61 |
| p0-0135.0497-003.79-splash10-001r-2900000000-3934387998d55c34c02f | Phenylacetic acid | Benzene and substituted derivatives | 3.53 |
| p0-0137.0255-007.07-splash10-002f-9340000000-ef2584ebe9e17e8bf7e2 | 4-Hydroxybenzoic acid | Benzene and substituted derivatives | 6.76 |
| p0-0137.0256-004.26-splash10-000i-2900000000-8275cfa8a6dc1c40cd3e | 3,4-Dihydroxybenzaldehyde | Benzene and substituted derivatives | 4 |
| p0-0153.0212-003.35-splash10-014i-9300000000-de3ca9ed4c17bcb4af19 | Protocatechuic acid | Benzene and substituted derivatives | 3.14 |
| p1-0105.0675-004.83-splash10-0fb9-9000000000-a99a938996104dc0a6e8 | 2-Phenylethanol | Benzene and substituted derivatives | 4.67 |
| p1-0105.0705-011.64-splash10-0fb9-9000000000-b0bc48e0d9e77d92d44d | Styrene | Benzene and substituted derivatives | 11.57 |
| p1-0107.0497-003.48-splash10-0fb9-9000000000-e8737622bd04de37675f | Benzaldehyde | Benzene and substituted derivatives | 3.54 |
| p1-0107.0842-013.68-splash10-00kf-9000000000-e71aa244db2587bf05aa | p-Xylene | Benzene and substituted derivatives | 13.7 |
| p1-0121.0637-005.68-splash10-004i-9000000000-2a8a8c77e9fca68e5f1a | 4-Methylbenzaldehyde | Benzene and substituted derivatives | 5.49 |
| p1-0127.0389-000.79-splash10-0gb9-9000000000-a8079f13a72ac67afdab | 1,2,3-Trihydroxybenzene | Benzene and substituted derivatives | 0.7 |
| p1-0133.0638-004.85-splash10-004i-9800000000-cd06448beee6423ec8dd | Atropaldehyde | Benzene and substituted derivatives | 4.67 |
| p1-0133.1025-005.69-splash10-014i-7900000000-1b6ae38c6a1f3e38fe34 | p-Mentha-1,3,5,8-tetraene | Benzene and substituted derivatives | 5.65 |
| p1-0137.0592-003.17-splash10-00kf-9200000000-3a2fd9de45bacfc4b0d3 | 4-Methoxybenzaldehyde | Benzene and substituted derivatives | 2.96 |
| p1-0139.0389-004.28-splash10-01b9-9300000000-67d5eea6bad055a39b6a | Salicylic acid | Benzene and substituted derivatives | 4.3 |
| p1-0165.0536-008.59-splash10-00kf-9500000000-07107dfefd124180848d | Enol-phenylpyruvate | Benzene and substituted derivatives | 8.42 |
| p1-0179.0684-008.42-splash10-014l-6900000000-86db656d00b3cba72f85 | (2E)-3-(4-hydroxy-3-methoxyphenyl)prop-2-enal | Benzene and substituted derivatives | 8.19 |
| p1-0181.0472-005.19-splash10-00n1-3900000000-8a3fdb8c86a6b0ff9626 | Aspirin | Benzene and substituted derivatives | 4.9 |
| p1-0223.0979-009.97-splash10-0005-0910000000-28da0e1b8346d10c30f5 | Diethyl phthalic acid | Benzene and substituted derivatives | 9.88 |
| p1-0231.0689-006.29-splash10-0bw9-0930000000-f01eaa3902df86c355c2 | 2',4'-Dihydroxy-2-biphenylcarboxylic acid | Benzene and substituted derivatives | 6.08 |
| p1-0127.0378-003.01-splash10-004i-6900000000-54ec55de4aca44639e7a | 5-Hydroxymethyl-2-furancarboxaldehyde | Benzene and substituted derivatives | 2.85 |
| p1-0333.0746-009.94-splash10-00kr-0092000000-fb8d84be204d01c35d65 | Fluorescein | Benzene and substituted derivatives | 9.7 |
| p0-0341.1175-003.82-splash10-002r-0900000000-2c24fc9944f2b5cb46e6 | beta-Lactose | Carboxylic acids and derivatives | 3.74 |
| p1-0134.0435-000.65-splash10-00di-9000000000-2ff8e6dacb27017bf055 | 1-deoxy-1-(N6-lysino)-D-fructose | Carboxylic acids and derivatives | 0.64 |
| p1-0146.0912-001.33-splash10-000i-9000000000-f8b178a67b83ec81247a | 4-Guanidinobutanoic acid | Carboxylic acids and derivatives | 1.13 |
| p1-0193.0332-001.18-splash10-02t9-9500000000-e285323acc49c8de368a | Citric acid | Carboxylic acids and derivatives | 1.24 |
| p1-0196.0949-003.32-splash10-000l-4900000000-f43a6cf7d6eb71e6b1ae | Tyrosine methylester | Carboxylic acids and derivatives | 3.13 |
| p1-0277.1247-008.99-splash10-0aor-0920000000-07003f30fb50e32ad8aa | Triethyl citrate | Carboxylic acids and derivatives | 8.84 |
| p1-0312.1457-010.35-splash10-014i-0190000000-4205e658a853dee2a1cb | Domoic acid | Carboxylic acids and derivatives | 10.26 |
| p1-0193.0469-005.92-splash10-001r-1900000000-fc2fb98377fd6f600da1 | Scopoletin | Coumarins | 5.7 |
| p1-0147.0428-005.77-splash10-00kf-9100000000-e7ba70532ea2cce8b224 | Coumarin | Coumarins | 5.75 |
| p1-0175.0364-008.24-splash10-00kf-9800000000-54a7eec3a36f6b8e6c92 | 1-Oxo-1H-2-benzopyran-3-carboxaldehyde | Coumarins | 7.99 |
| p1-0177.0534-005.10-splash10-000i-9700000000-295afe972e5ffd12ff58 | Herniarin | Coumarins | 5.15 |
| p1-0193.0484-006.08-splash10-000i-2900000000-9ea7db6547d49b0fd1c0 | 5,7-DIHYDROXY-4-METHYLCOUMARIN | Coumarins | 6.09 |
| p0-0177.0211-003.33-splash10-001i-5900000000-df6eb5770d39040902dd | Aesculetin | Coumarins | 3.11 |
| p1-0209.0450-004.53-splash10-0w29-1900000000-f461014e79b0c29da879 | FRAXETIN | Coumarins | 4.31 |
| p1-0285.0370-008.94-splash10-000i-0290000000-2eecaff57063b060faf3 | 7,4',5'-Trihydroxy-5,2'-oxido-4-phenylcoumarin | Coumarins | 8.96 |
| p1-0339.1548-002.86-splash10-0002-0910000000-b16bca405f853f9a5458 | Bergamottin | Coumarins | 3.15 |
| p1-0325.0909-007.13-splash10-03di-0910000000-955f799f5ff0e938cb5b | Mahaleboside | Coumarins | 7.33 |
| p1-0335.0383-004.70-splash10-000i-0459000000-d803c7ac45915389d279 | 3,5,6,7,2',3',4'-Heptahydroxyflavone | Flavonoids | 4.5 |
| p0-0255.0311-006.43-splash10-056s-0790000000-1f7cfda552dc4cc6414e | Purpurin | Flavonoids | 6.45 |
| p0-0257.0478-008.96-splash10-0udi-0390000000-fdc88d762dca8daaeea3 | 2,6,7-Trihydroxy-9-methylxanthen-3-one | Flavonoids | 8.74 |
| p0-0271.0641-006.71-splash10-0gb9-0920000000-5c268909d5ba675b595c | (±)-Naringenin | Flavonoids | 6.54 |
| p0-0301.0373-006.63-splash10-0kmi-0690000000-3c16e2812759fa7c1a06 | Morin | Flavonoids | 6.46 |
| p0-0301.0402-008.13-splash10-0udi-0911000000-cd1f05acb2339aa8015c | Quercetin | Flavonoids | 7.88 |
| p0-0343.0849-011.45-splash10-03di-0198000000-bc996fbd3bd83e57162e | Nevadensin | Flavonoids | 11.39 |
| p0-0373.0954-011.19-splash10-0006-0019000000-00bfc82b9f709dee60b9 | Casticin | Flavonoids | 11.08 |
| p0-0417.0856-006.87-splash10-0as0-0090200000-14c6ecefbb19c635e6d7 | Kaempferol-3-O-arabinopyranoside | Flavonoids | 6.84 |
| p0-0417.0857-006.79-splash10-05q9-0090200000-5c37cf051ed8dba462e3 | Kaempferol-3-O-alpha-L-arabinoside | Flavonoids | 6.6 |
| p0-0447.0965-006.42-splash10-053b-0090300000-bbb9363a909f62fbc953 | Astragalin | Flavonoids | 6.41 |
| p0-0449.0750-005.73-splash10-014i-0049300000-47762a9558cac30b8a6a | Myricetin-3-Xyloside | Flavonoids | 5.76 |
| p0-0457.0787-005.16-splash10-014i-0910000000-544278bd4fead3ce7aed | Epigallocatechin gallate | Flavonoids | 4.93 |
| p0-0463.0964-006.15-splash10-0udi-0019100000-47036eea6c587570d81a | Isoquercitrin | Flavonoids | 5.86 |
| p0-0477.1091-007.78-splash10-03di-0019100000-40f70efaa1c498bbf44e | Isorhamnetin 3-glucoside | Flavonoids | 7.61 |
| p0-0479.0843-005.53-splash10-014i-0139100000-bc43b8b1403ccdb4c1cf | Myricetin 3-galactoside | Flavonoids | 5.35 |
| p0-0479.0847-006.21-splash10-014i-0139200000-e6416c06cc9be6230d89 | Myricetin-3-Galactoside | Flavonoids | 5.9 |
| p1-0161.0585-005.96-splash10-014i-2900000000-d6da856dab868bba2dbc | 6-Methylcoumarin | Flavonoids | 5.78 |
| p1-0166.0846-001.41-splash10-00dr-3900000000-7b3326bfb1a2b4acfd53 | 2-amino-4'-hydroxy-Propiophenone | Flavonoids | 1.49 |
| p1-0203.1782-011.53-splash10-05pp-2900000000-ddf9c36c9afb373255ca | alpha-curcumene | Flavonoids | 11.48 |
| p1-0207.0634-006.19-splash10-00kg-3900000000-b10d55bcd2a8e29c09f5 | Scoparone | Flavonoids | 6 |
| p1-0255.0623-011.00-splash10-0a4i-0390000000-044b3c78a987237f4c1a | 5,7-Dihydroxyflavone | Flavonoids | 10.8 |
| p1-0257.0790-011.11-splash10-0udi-0910000000-2bcc9a6835f6f8277ea4 | (S)-Pinocembrin | Flavonoids | 10.97 |
| p1-0259.0631-006.30-splash10-0002-0920000000-e041f4456ac85dc3f8e9 | Isogentisin | Flavonoids | 6.08 |
| p1-0271.0574-008.94-splash10-00di-0490000000-92dd8ada6a080674d2c7 | Apigenin | Flavonoids | 8.72 |
| p1-0275.0893-007.06-splash10-0a4i-0900000000-a979f7ca13faab80c871 | Phloretin | Flavonoids | 6.86 |
| p1-0287.0534-007.21-splash10-000i-0390000000-f35faa99fedd0ce222e5 | Kaempferol | Flavonoids | 7.19 |
| p1-0289.1168-003.21-splash10-0079-0910000000-cefa456c9cc27528c486 | Dihydrocalythropsin | Flavonoids | 3.15 |
| p1-0291.0841-004.59-splash10-0079-0900000000-ea6ed6c79ba23b156b5f | Catechin | Flavonoids | 4.3 |
| p1-0301.0675-008.55-splash10-0pc0-1942000000-fbca8cf98581d80c4cda | Luteolin 5-methyl ether | Flavonoids | 8.56 |
| p1-0305.0632-005.69-splash10-0ff1-0940000000-62e0535dbf0f5be7a286 | (±)-Taxifolin | Flavonoids | 5.51 |
| p1-0305.0654-004.52-splash10-001j-0790000000-9264c691e1ed8867a4b9 | Taxifolin | Flavonoids | 4.23 |
| p1-0317.0618-006.80-splash10-0gb9-0269000000-efce1036c0f1b41f6293 | Tamarixetin | Flavonoids | 6.83 |
| p1-0317.0625-006.60-splash10-0udr-0392000000-3505e0aa390ae8fbb02a | Isorhamnetin | Flavonoids | 6.43 |
| p1-0317.0629-007.48-splash10-0udi-0149000000-0f2ecf6e0356f35e945a | 3-methylquercetin | Flavonoids | 7.62 |
| p1-0319.0426-005.15-splash10-014i-0894000000-5c56d762f38f4cff7a91 | Myricetin | Flavonoids | 5.36 |
| p1-0327.1079-005.05-splash10-0002-0910000000-59ce7613b730055dd29d | Coumaric acid O-glucoside | Flavonoids | 5.01 |
| p1-0331.0780-009.62-splash10-014i-0019000000-b362ad69286963570ed2 | 4',5,7-trihydroxy-3,6-dimethoxyflavone | Flavonoids | 9.44 |
| p1-0361.0886-007.63-splash10-01qa-0019000000-a0a70164c4779e23ee84 | IRIGENIN | Flavonoids | 7.48 |
| p1-0433.1151-004.83-splash10-00di-0090000000-35ee227332245e0cbe47 | Genistein 4'-O-glucoside | Flavonoids | 4.5 |
| p1-0447.1244-009.14-splash10-0udi-0009000000-fff0b5871edecb19fb63 | 3,6,7-Trihydroxy-4'-methoxyflavone 7-rhamnoside | Flavonoids | 8.99 |
| p1-0449.1067-004.55-splash10-000i-0090000000-43ac0bd2b415cfb9a0b8 | Quercitrin | Flavonoids | 4.22 |
| p1-0449.1082-006.44-splash10-000i-0090000000-a6868b506cb70b455469 | Kaempferol 3-alpha-D-glucoside | Flavonoids | 6.38 |
| p1-0451.0830-005.73-splash10-014i-0009000000-51f79da4b199c32da858 | myricetin-3-O-pentoside | Flavonoids | 5.76 |
| p1-0451.1193-004.80-splash10-05a6-0494000000-f9847d1b5dd98879bc92 | Miscanthoside | Flavonoids | 4.74 |
| p1-0463.0827-003.42-splash10-000i-0193000000-15909dc5f887fa01ea9d | Kaempferol glucuronide | Flavonoids | 3.21 |
| p1-0477.1357-006.09-splash10-0udi-0922000000-554b868ced538d45070c | 7,2'-Dihydroxy-3',4'-dimethoxyisoflavone 7-O-glucoside | Flavonoids | 5.94 |
| p1-0481.0936-005.54-splash10-014i-0009000000-82b407e6edf6cacc243f | Myricetin 3-glucoside | Flavonoids | 5.35 |
| p1-0495.0754-005.55-splash10-014i-0009000000-61575ec67b6deb2c8884 | Myricetin 3-glucuronide | Flavonoids | 5.36 |
| p1-0495.1045-007.11-splash10-001i-0129200000-f3d3d36972a586f30e02 | Laricitrin 3-glucoside | Flavonoids | 6.93 |
| p1-0521.0795-010.67-splash10-0ukc-0014980000-b55021e7229faeaffe90 | Pseudohypericin | Flavonoids | 10.53 |
| p1-0595.1613-006.63-splash10-0udi-0209310000-743ce186cecdc6dbfa19 | Quercetin-3,7-O-alpha-L-dirhamnopyranoside | Flavonoids | 6.44 |
| p1-0627.1260-007.35-splash10-014j-0619303000-6af5dad49fde19bfc72c | Myricetin 3'-O-(6''-p-coumaroyl)glucoside | Flavonoids | 7.16 |
| p1-0639.2232-007.66-splash10-001i-0009100000-94aef7163f89df4b636b | 4'-Hydroxy-5,7,2'-trimethoxyflavanone 4'-rhamnosyl-(1->6)-glucoside | Flavonoids | 7.55 |
| p1-0899.1956-003.38-splash10-0a4m-0132974200-5996de75081feb799927 | [Gallocatechin(4alpha->8)]2catechin | Flavonoids | 3.17 |
| p1-0195.0628-006.94-splash10-0f79-0900000000-074b719e83b048273407 | Erbstatin Analog | Isoflavonoids | 6.75 |
| p1-0271.0550-005.16-splash10-01ba-0960000000-39eb83022d12258458b1 | Genistein | Isoflavonoids | 4.94 |
| p1-0285.0737-011.15-splash10-000i-0090000000-a5c7d1a2393961a3afac | Prunetin | Isoflavonoids | 11.25 |
| p0-0251.0699-004.43-splash10-000i-0900000000-2e4faccc542149d44ed2 | Deoxyinosine | Nucleotide and derivates | 4.23 |
| p0-0283.0705-002.81-splash10-0udi-0910000000-dfd1a98ee8eafe063de3 | Xanthosine | Nucleotide and derivates | 2.53 |
| p1-0113.0343-002.26-splash10-006t-9000000000-df91a77426a8f9d35e96 | Uracil | Nucleotide and derivates | 2.6 |
| p1-0136.0616-000.96-splash10-014i-3900000000-1df984cd5064ef50a00b | Adenine | Nucleotide and derivates | 1.11 |
| p1-0153.0394-001.97-splash10-06s9-7900000000-811e5e7c2009dcc8f7a7 | Xanthine | Nucleotide and derivates | 2.24 |
| p1-0284.0982-002.52-splash10-0udi-0900000000-e6146946599aa95fe505 | Guanosine | Nucleotide and derivates | 2.18 |
| p0-0179.0580-000.73-splash10-0570-9100000000-6fae454c1771da84b45b | myo-Inositol | Organooxygen compounds | 0.61 |
| p0-0191.0574-000.75-splash10-0006-8900000000-898968f80f5091dfd46b | Quinic acid | Organooxygen compounds | 0.71 |
| p0-0377.0913-000.81-splash10-0006-4907000000-55c56ad438f07d18d948 | Melibiose | Organooxygen compounds | 0.73 |
| p1-0139.0374-001.10-splash10-066r-9000000000-8acc78093f153fd970fd | Gentisate aldehyde | Organooxygen compounds | 0.97 |
| p1-0151.0586-001.25-splash10-03di-9000000000-9dc5d8efaa85bae04dc8 | D-Apiose | Organooxygen compounds | 1.13 |
| p1-0153.0534-004.38-splash10-000i-1900000000-7f41012f6e16dfab5b91 | 3',5'-Dihydroxyacetophenone | Organooxygen compounds | 4.26 |
| p1-0163.0588-000.90-splash10-0670-9000000000-56a3eea117ae08f27fce | D-Tagatose | Organooxygen compounds | 0.73 |
| p1-0261.0306-000.87-splash10-0002-9100000000-306e87cd05e8b7216a5d | Galactose 1-phosphate | Organooxygen compounds | 0.65 |
| p1-0343.1243-000.98-splash10-002s-7900000000-563257422db0afcb94b6 | D-Maltose | Organooxygen compounds | 0.72 |
| p0-0287.0582-007.11-splash10-004i-0930000000-7514e72803f00d33ce19 | 3,5,7-trihydroxy-2-(4-hydroxyphenyl)-3,4-dihydro-2H-1-benzopyran-4-one | Others | 6.89 |
| p0-0329.0690-009.62-splash10-006t-0191000000-e1e44d0ce2f96f274f99 | 5,7-dihydroxy-2-(4-hydroxy-3,5-dimethoxyphenyl)-4H-chromen-4-one | Others | 9.44 |
| p0-0465.1051-005.14-splash10-0f79-0891000000-d1cf19c19cbc0b32d90f | (2R,3R)-2-(3,4-dihydroxyphenyl)-5,7-dihydroxy-3-[(2S,3R,4S,5S,6R)-3,4,5-trihydroxy-6-(hydroxymethyl)oxan-2-yl]oxy-2,3-dihydrochromen-4-one | Others | 5.05 |
| p0-0477.1035-008.49-splash10-014i-0229200000-ca4b3fb0748d1324568f | 5,7-dihydroxy-2-(4-hydroxy-3-methoxyphenyl)-3-{[3,4,5-trihydroxy-6-(hydroxymethyl)oxan-2-yl]oxy}-4H-chromen-4-one | Others | 8.48 |
| p1-0177.0525-005.45-splash10-0kcr-3900000000-8575277dfa3562154288 | 7-Hydroxy-6-methyl-2H-1-benzopyran-2-one | Others | 5.25 |
| p1-0227.1621-005.06-splash10-0002-8900000000-97f1bc1b997d5456e388 | 2-(7-hydroxy-6-methyloctyl)-2H-furan-5-one | Others | 4.95 |
| p1-0244.1898-009.30-splash10-053r-9600000000-db10778c9aad6a1b220c | 2-(6-hydroxy-6-methyloctyl)-2H-furan-5-one | Others | 9.16 |
| p1-0305.0634-006.28-splash10-0h9v-0950000000-c5454d38d29b610dd358 | 2-(3,4-dihydroxyphenyl)-3,5,7-trihydroxy-3,4-dihydro-2H-1-benzopyran-4-one | Others | 6.34 |
| p1-0317.0650-010.18-splash10-014i-0339000000-9270862e21330b7e1807 | 2-(3,4-dihydroxyphenyl)-3,5-dihydroxy-7-methoxy-4H-chromen-4-one | Others | 9.96 |
| p1-0325.0900-007.37-splash10-03di-1900000000-80ce26bdfea38910e593 | 7-[(2S,3R,4S,5S,6R)-3,4,5-trihydroxy-6-(hydroxymethyl)oxan-2-yl]oxychromen-2-one | Others | 7.57 |
| p1-0347.0732-008.50-splash10-0159-0019000000-ff4666abaa51dc37cef0 | 2-(3,4-dihydroxyphenyl)-5,7-dihydroxy-6,8-dimethoxy-4H-chromen-4-one | Others | 8.28 |
| p1-0355.1006-007.87-splash10-0006-0910000000-2ad8fba988f19316bce6 | 7-methoxy-6-[(2S,3R,4S,5S,6R)-3,4,5-trihydroxy-6-(hydroxymethyl)oxan-2-yl]oxychromen-2-one | Others | 7.7 |
| p1-0359.1094-008.33-splash10-0006-0009000000-2f08f47f6c4a85bd6022 | QUERCETIN TETRAMETHYL (5,7,3',4') ETHER | Others | 8.19 |
| p1-0433.1105-006.00-splash10-03e9-0049000000-9bd2ef95672ad76dc9f6 | 5,7-dihydroxy-2-(4-hydroxyphenyl)-6-[3,4,5-trihydroxy-6-(hydroxymethyl)oxan-2-yl]-4H-chromen-4-one | Others | 5.82 |
| p1-0437.3350-011.99-splash10-0ue9-1898600000-7dcbe811060c29b7f17b | (3beta,5xi,9xi,13alpha,17alpha,18xi)-3-Hydroxy-13,28-epoxyurs-11-en-28-one | Others | 11.88 |
| p1-0479.1148-006.84-splash10-001i-0009000000-64f79b507c693fcd1b9a | 2-(3,4-dihydroxyphenyl)-5,8-dihydroxy-7-methoxy-3-[(2S,3R,4R,5R,6S)-3,4,5-trihydroxy-6-methyloxan-2-yl]oxychromen-4-one | Others | 6.66 |
| p1-0227.0526-003.52-splash10-0w29-0900000000-3213583f9b6a6aaa927d | Prephenate | Others | 3.36 |
| p0-0173.0243-008.16-splash10-0002-0900000000-707afe118e324456776a | LAWSONE | Others | 7.99 |
| p0-0271.0280-006.52-splash10-00di-0390000000-946e675246796979dd10 | QUINALIZARIN | Others | 6.46 |
| p0-0297.1559-012.55-splash10-000t-0890000000-c4fcec1b6e2451f29409 | C10-LAS (SAMPLE) | Others | 12.25 |
| p0-0433.1167-005.77-splash10-03di-0229000000-451fc0fe24811eefb11a | 5,7-dihydroxy-2-(4-hydroxyphenyl)-6-[3,4,5-trihydroxy-6-(hydroxymethyl)oxan-2-yl]-2,3-dihydrochromen-4-one | Others | 5.57 |
| p0-0487.1486-004.55-splash10-000j-0972600000-180628fdd48941d65693 | alpha-D-Glucopyranoside, beta-L-fructofuranosyl 6-O-[(2E)-3-(4-hydroxyphenyl)-1-oxo-2-propen-1-yl]- | Others | 4.65 |
| p0-0489.1864-005.17-splash10-0006-0410900000-ccd2214674034ec41460 | Furo[4,3,2-ij]pyrano[4',3':4,5]cyclohepta[1,2-f][2]benzopyran-1,5,9,13(2H,11H)-tetrone, 3a,5a,6,8,12,13a,13b,13c-octahydro-7-(hydroxymethyl)-2,5a,11,11,13a-pentamethyl- | Others | 5 |
| p0-0493.2326-007.96-splash10-0002-0303900000-4abfbf4c7757824056dd | (6,6-Dimethylbicyclo[3.1.1]hept-2-yl)methyl 6-O-[(2R,3R,4R)-3,4-dihydroxy-4-(hydroxymethyl)tetrahydro-2-furanyl]-beta-D-glucopyranoside | Others | 7.85 |
| p0-0565.0493-000.75-splash10-00di-0129030000-caaabbf78d1326ea4e55 | Uridine diphosphategalactose | Others | 0.68 |
| p0-0599.1079-006.14-splash10-03dj-0359530000-126546adfac6a3505d8f | [6-[5,7-dihydroxy-2-(4-hydroxyphenyl)-4-oxochromen-3-yl]oxy-3,4,5-trihydroxyoxan-2-yl]methyl 3,4,5-trihydroxybenzoate | Others | 5.97 |
| p1-0137.0674-001.11-splash10-00di-9800000000-1700377516dcb690847c | PRALIDOXIME | Others | 1.11 |
| p1-0137.1329-006.48-splash10-0axu-9600000000-37d5f056b14108c0f844 | 2,6-Dimethyl-2,4E,6E-octatriene | Others | 6.39 |
| p1-0164.0403-007.97-splash10-0006-9400000000-ce8fd6025c99829d3ec3 | Benzotriazole-5-carboxylic acid | Others | 7.75 |
| p1-0169.1208-008.30-splash10-001i-9100000000-e48e539a974926d6b874 | 8-Epiiridodial | Others | 8.16 |
| p1-0175.1506-010.26-splash10-00pl-6900000000-1a844e16ad7d0d496df5 | 5,7alpha-Dihydro-1,4,4,7a-tetramethyl-4H-indene | Others | 10.26 |
| p1-0177.0527-007.62-splash10-0fhc-5900000000-829d87059e7579ff1288 | 4-phenyl-5-methyl-1,2,3-Thiadiazole | Others | 7.82 |
| p1-0179.0684-004.85-splash10-014i-0900000000-e97ac48a3e44415a068a | (R)-(-)-Mellein | Others | 4.67 |
| p1-0190.0490-004.24-splash10-0006-0900000000-2e7b0ef82df4295bc76f | Kynurenic acid | Others | 4.01 |
| p1-0191.0294-008.06-splash10-03di-1900000000-8dac9f4f5d8e9675f026 | Ayapin | Others | 7.84 |
| p1-0193.1571-010.30-splash10-0a5c-9800000000-c7128fbe9ccab0658c08 | beta-Ionone | Others | 10.25 |
| p1-0195.0646-004.95-splash10-014s-4900000000-7fb443d66b9208400a1c | Scytalone | Others | 4.9 |
| p1-0195.1347-011.44-splash10-01p9-2900000000-b8d710012b0988a49a6d | 2-(6-hydroxy-6-methylheptyl)-2H-furan-5-one | Others | 11.37 |
| p1-0197.1156-006.61-splash10-0a5c-3900000000-fd32aea8a8996f38d2c0 | 2(4H)-Benzofuranone, 5,6,7,7a-tetrahydro-6-hydroxy-4,4,7a-trimethyl-, (6S,7aR)- | Others | 6.41 |
| p1-0207.0633-007.88-splash10-014m-3900000000-90f9c2a8a3f5345a76af | 2-Hydroxy-8-methylchromene-2-carboxylate | Others | 7.74 |
| p1-0209.1510-005.06-splash10-0fr5-4900000000-edb780f8c2f15b1d93f4 | 1H-Indene-3-carboxylic acid, 3a,4,5,6,7,7a-hexahydro-3a,7,7-trimethyl- | Others | 4.95 |
| p1-0209.1511-004.10-splash10-0a4i-8900000000-48da81c938874832573f | 2,2,6,7-Tetramethylbicyclo[4.3.0]nona-1(9),4-diene-7,8-diol | Others | 4 |
| p1-0209.1516-006.55-splash10-0a4i-4900000000-6ab1be3a3c64807fa0bd | 2-Cyclohexen-1-one, 4-hydroxy-4-(3-hydroxybutyl)-3,5,5-trimethyl- | Others | 6.51 |
| p1-0219.1729-013.15-splash10-0apm-2900000000-b0abb183d8181bca9add | NCGC00385845-01 | Others | 12.97 |
| p1-0221.0420-005.83-splash10-0pdi-0910000000-519d7d6a0418cd89e0ac | (3E)-4-(2-Carboxyphenyl)-2-oxobut-3-enoate | Others | 5.67 |
| p1-0225.0720-006.42-splash10-00ke-1900000000-6becca326f2e6488f9c8 | 6,8-dihydroxy-7-methoxy-3-methyl-3,4-dihydroisochromen-1-one | Others | 6.23 |
| p1-0225.1952-010.61-splash10-06u6-9620000000-07162b942e56064f60f3 | N,N'-Dicyclohexylurea | Others | 10.57 |
| p1-0235.1665-011.98-splash10-0a70-8940000000-2864f5f6bd0d35192d12 | Confertifoline | Others | 11.92 |
| p1-0243.1000-012.09-splash10-00ba-0910000000-23e8ede7080d858da9a7 | Lapachol | Others | 12.04 |
| p1-0247.1316-005.20-splash10-0007-0900000000-514d4136f4af12c05f6d | 1H-3a,6-Epoxyazulene-7-acetic acid, octahydro-4,8a-dimethyl-alpha-methylene-1-oxo-, (3aR,4S,6S,7R,8aS)- | Others | 5.27 |
| p1-0247.1322-007.91-splash10-0079-0900000000-5c7fcf4e498e0d8c374c | Octopine | Others | 7.73 |
| p1-0255.1584-010.79-splash10-0a4s-2900000000-328c528fb434b542238e | (+)-trans-C75 | Others | 10.62 |
| p1-0256.1017-011.39-splash10-01ot-1940000000-5852eddddcc68cd15699 | 3-Methoxy-8,9-methylenedioxy-3,4-dihydrophenanthridine | Others | 11.3 |
| p1-0259.0570-008.12-splash10-0btl-0950000000-bfc2eb043d96c665d01e | Alternariol | Others | 7.88 |
| p1-0259.0581-003.01-splash10-03di-1590000000-0157f95300f53d2506b3 | 3-Carbethoxypsoralen | Others | 2.96 |
| p1-0265.1428-006.12-splash10-0a7i-0900000000-d0a5170504fc0bd19748 | NCGC00381407-01 | Others | 5.97 |
| p1-0266.0994-005.00-splash10-03di-0900000000-bbd2990f9692b1b9df92 | CAY10487 | Others | 5.06 |
| p1-0287.0530-009.09-splash10-000i-0390000000-75f33098f75101b9a6e0 | Fisetin | Others | 8.87 |
| p1-0287.0549-006.43-splash10-000i-0390000000-d567c73009ac85f774f9 | Fisetin | Others | 6.39 |
| p1-0287.1259-011.27-splash10-0ar1-0950000000-ac1f496c34aa6845bf60 | CAY10616 | Others | 11.18 |
| p1-0289.0690-007.63-splash10-0gba-0950000000-18fd5116c1b23dc0e72d | 2,4,6-trihydroxy-2-[(4-hydroxyphenyl)methyl]-2,3-dihydro-1-benzofuran-3-one | Others | 7.42 |
| p1-0291.2101-010.60-splash10-0zfr-0930000000-6406b0c657a861ca34e1 | Verapamil metabolite D-617 | Others | 10.46 |
| p1-0300.1773-005.23-splash10-05ra-0910000000-64decb03e0e222c2c58c | NCGC00170013-02 | Others | 5.06 |
| p1-0303.0838-004.90-splash10-0iki-0891000000-5b70255b54bfc7fc37a9 | Nanaomycin | Others | 4.71 |
| p1-0309.0944-006.95-splash10-0002-0900000000-27902ce2b8ecd3bbbaf2 | beta-D-Glucopyranose, 1-O-[(2E)-3-(2-hydroxyphenyl)-1-oxo-2-propen-1-yl]- | Others | 7.01 |
| p1-0314.0971-002.41-splash10-000i-2910000000-17375b0c8dec24c05e27 | (S)-5'-Deoxy-5'-(methylsulfinyl)adenosine | Others | 2.12 |
| p1-0315.0870-007.19-splash10-0udi-0049000000-c46dd1bd001a578c8a8d | 5-hydroxy-7-(hydroxymethyl)-2-methyl-2-(5-oxooxolan-2-yl)-3H-chromen-4-one | Others | 7.06 |
| p1-0317.1348-006.22-splash10-0f9i-0970000000-03bc77ac86bce553f0d8 | (5E,11E)-7,8,14,16-Tetrahydroxy-3-methyl-3,4,7,8,9,10-hexahydro-1H-2-benzoxacyclotetradecin-1-one | Others | 6.04 |
| p1-0321.0575-005.51-splash10-0f6t-0950000000-618117c5e2592872f4d2 | fuzhuanin A | Others | 5.54 |
| p1-0331.1540-007.53-splash10-0ug0-0970000000-1dd1b86f30ebdf8737ad | Naphtho[2,3-b]furan-9(4H)-one, 4,8-bis(acetyloxy)-4a,5,6,7,8,8a-hexahydro-3,4a,5-trimethyl-, (4S,4aR,5S,8S,8aS)- | Others | 7.36 |
| p1-0333.2393-013.86-splash10-053s-3911000000-114b314ec0cd8d1504ba | 5-cis Carbaprostacyclin | Others | 13.91 |
| p1-0335.2548-011.52-splash10-00ks-4911000000-320a2b4c59d2c921d5da | (±)8(9)-EET methyl ester | Others | 11.46 |
| p1-0337.0877-004.55-splash10-03di-0900000000-f9c7f397ecf42cec4f36 | (3R,4R,5R)-5-[(E)-3-(3,4-dihydroxyphenyl)prop-2-enoyl]oxy-3,4-dihydroxycyclohexene-1-carboxylic acid | Others | 4.3 |
| p1-0341.1351-006.49-splash10-01p9-0390000000-c182c38a5a5d1d569905 | 2-Butenoic acid, 2-methyl-, (3aR,4R,5R,9aS,9bR)-2,3,3a,4,5,7,9a,9b-octahydro-4-hydroxy-6,9-dimethyl-3-methylene-2,7-dioxoazuleno[4,5-b]furan-5-yl ester, (2Z)- | Others | 6.34 |
| p1-0351.2501-010.07-splash10-0a4l-1930000000-02c0c7eb90eacb0bec4c | U-44069 | Others | 9.98 |
| p1-0353.2662-011.50-splash10-001j-3911000000-aed603a2e939a7fa296a | 9,11-methane-epoxy PGF1α | Others | 11.46 |
| p1-0355.2805-011.98-splash10-029t-5931000000-2e6f288a4a140c341881 | MG(18:2(9Z,12Z)/0:0/0:0) | Others | 11.94 |
| p1-0357.3246-012.21-splash10-0a4i-1941000000-bf8306c557121ea5c9ae | CONESSINE | Others | 12.15 |
| p1-0362.1495-004.22-splash10-00xr-1900000000-c12275e4301af5c5be74 | NCGC00380817-01 | Others | 4.22 |
| p1-0369.1141-004.73-splash10-004i-0900000000-4d0a469d1576da81a6ad | Cyclohexanecarboxylic acid, 1,3,5-trihydroxy-4-[[(2E)-3-(4-hydroxy-3-methoxyphenyl)-1-oxo-2-propen-1-yl]oxy]-, (1alpha,3alpha,4alpha,5beta)- | Others | 4.5 |
| p1-0371.2062-006.52-splash10-001r-0900000000-804dd90e07976e87c35d | 2-Butanone, 4-[3-(beta-D-glucopyranosyloxy)-4-hydroxy-2,6,6-trimethyl-1-cyclohexen-1-yl]- | Others | 6.4 |
| p1-0375.2345-005.49-splash10-004i-0900000000-e06259e7c3c20a999cef | 4-(4-Hydroxy-2,6,6-trimethyl-1-cyclohexen-1-yl)-2-butanyl beta-D-glucopyranoside | Others | 5.38 |
| p1-0377.1097-011.18-splash10-01r2-0009000000-e9c070e525de71d019d3 | RESISTOMYCIN | Others | 11.08 |
| p1-0389.2133-005.08-splash10-0ac4-1930000000-8be4d886e7740ca198a1 | icariside B5 | Others | 4.95 |
| p1-0397.3088-011.65-splash10-0002-0942000000-d63908af58ec07e4b6c9 | Spirost-5-en-3-ol, (3beta,25R)- | Others | 11.62 |
| p1-0421.1572-003.78-splash10-0a4i-0290100000-e260059e31a9c62f2fe6 | Shanciol | Others | 3.58 |
| p1-0427.1882-006.47-splash10-004i-0051900000-bc6fe99b55ad2eea3b76 | gamma-CEHC Glc | Others | 6.65 |
| p1-0433.1094-007.18-splash10-000i-1090000000-f322e2ae5275500c12f1 | Afzelin | Others | 7.17 |
| p1-0471.1598-008.52-splash10-0002-0910000000-03b4c1c3ee2b154c9ac8 | Hexopyranose, 2-O-hexopyranosyl-6-O-[(2E)-3-(4-hydroxyphenyl)-1-oxo-2-propen-1-yl]- | Others | 8.36 |
| p1-0480.2426-008.30-splash10-0fsi-2910000000-ca1daf34836d3fc2bb57 | [3,4,5-trihydroxy-6-[(3,4,5-trihydroxyoxan-2-yl)oxymethyl]oxan-2-yl] 2,6,6-trimethylcyclohexene-1-carboxylate | Others | 8.17 |
| p1-0496.3359-011.96-splash10-001i-0900000000-bfdb0785ec0be2346091 | PC(16:0/0:0)[U] / PC(16:0/0:0)[rac] | Others | 11.97 |
| p1-0532.3433-011.78-splash10-03di-1893000000-3e9e7441e0b9a91a41f1 | 9,12,15-Octadecatrienoic acid, 3-(hexopyranosyloxy)-2-hydroxypropyl ester, (9Z,12Z,15Z)- | Others | 11.74 |
| p1-0540.2391-006.02-splash10-02ar-0398000000-fc94571b195da75d35dd | 2-(hydroxymethyl)-6-[5-[3-(hydroxymethyl)-5-(3-hydroxypropyl)-7-methoxy-2,3-dihydro-1-benzofuran-2-yl]-2-methoxyphenoxy]oxane-3,4,5-triol | Others | 5.9 |
| p1-0551.2181-004.71-splash10-000i-0119040000-78579189b27f7bdcaeb6 | Eucommin A | Others | 4.69 |
| p1-0556.2309-004.96-splash10-01p9-0946000000-2c4429afc6db51470ad6 | 4-{[(3S,4R,5S)-3-Hydroxy-5-(4-hydroxy-3-methoxyphenyl)-4-(hydroxymethyl)tetrahydro-3-furanyl]methyl}-2-methoxyphenyl beta-D-glucopyranoside | Others | 4.93 |
| p1-0595.1365-008.19-splash10-000j-0591100000-7760b42eaf4ea3d3a8f6 | [6-[2-(3,4-dihydroxyphenyl)-8-hydroxy-4-oxochromen-7-yl]oxy-3,4,5-trihydroxyoxan-2-yl]methyl (E)-3-(4-hydroxyphenyl)prop-2-enoate | Others | 8.01 |
| p1-0617.1806-007.80-splash10-0002-0910000000-067e113dbed6f984eae9 | Spiro[cyclopenta[c]pyran-7(1H),2'(5'H)-furan]-4-carboxylic acid, 1-(beta-D-glucopyranosyloxy)-4a,7a-dihydro-4'-[(1S)-1-[[(2E)-3-(4-hydroxyphenyl)-1-oxo-2-propen-1-yl]oxy]ethyl]-5'-oxo-, methyl ester, (1S,4aS,7R,7aS)- | Others | 7.63 |
| p1-0677.3689-011.48-splash10-0ik9-0597101000-fbdeb7d1218fb349be38 | Gingerglycolipid A | Others | 11.47 |
| p0-0595.1353-005.92-splash10-0w29-0016930000-edcb61ffcfad07272210 | Rhodalidin | Others | 5.56 |
| p1-0355.0707-005.01-splash10-03di-0900000000-9a01bb1c3f692e69f2c6 | 2-Caffeoylisocitrate | Others | 4.81 |
| p1-0369.1504-005.38-splash10-0ar1-0943000000-e9b758704d914fa0163c | Methyl (R)-9-hydroxy-10-undecene-5,7-diynoate glucoside | Others | 5.25 |
| p0-0152.0724-001.64-splash10-00di-0900000000-bdadccbc9cb292eeebca | Dopamine | Phenolamides | 1.36 |
| p1-0122.0955-003.50-splash10-004i-9400000000-9a73ce65ed9a89b45adf | Phenylethylamine | Phenolamides | 3.23 |
| p1-0198.1842-011.53-splash10-0aor-9200000000-3f01f037479f10c6e234 | N-methylundec-10-enamide | Phenolamides | 11.56 |
| p1-0256.2611-012.89-splash10-0a4i-7390000000-fca419c452f6b9905933 | Palmitic amide | Phenolamides | 12.76 |
| p1-0288.2513-011.57-splash10-0a70-9660000000-79bcffca46b1eb1be5cf | Lauroyl diethanolamide | Phenolamides | 11.53 |
| p1-0341.1359-008.46-splash10-01pc-0390000000-79c1b4a92d0a194fcff7 | 5-[6-(3-hydroxy-4-methoxyphenyl)-1,3,3a,4,6,6a-hexahydrofuro[3,4-c]furan-3-yl]-2-methoxyphenol | Phenolamides | 8.28 |
| p0-0144.0463-002.67-splash10-0006-0900000000-930d3107d5e51f923c7f | Quinolone | Phenolic acids | 2.45 |
| p0-0625.1432-005.36-splash10-0h00-0016908000-4651e5fc34ad5470430c | Quercetin-3,4'-O-di-beta-glucopyranoside | Phenolic acids | 5.41 |
| p1-0133.0639-003.59-splash10-004i-9800000000-b28f94b397594bc15015 | Cinnamaldehyde | Phenolic acids | 3.43 |
| p1-0181.0481-007.95-splash10-000i-8900000000-cd09da28cfc22a044f1a | 3,4-Dihydroxy-trans-cinnamate | Phenolic acids | 7.76 |
| p1-0195.0638-007.99-splash10-014s-3900000000-897a60b6ff00c315ea7d | 3-(4-hydroxy-3-methoxyphenyl)prop-2-enoic acid | Phenolic acids | 7.82 |
| p1-0357.1147-008.34-splash10-004j-0922000000-81143c10cae303c34b93 | 1-O-Feruloylglucose | Phenolic acids | 8.19 |
| p0-0161.0265-003.68-splash10-014i-1900000000-e8f523b1664aa9485ec0 | 4-Coumaryl alcohol | Phenolic acids | 3.75 |
| p0-0163.0476-007.80-splash10-014i-2900000000-1e63550ab427dd57b835 | 2-Hydroxycinnamic acid | Phenolic acids | 7.64 |
| p0-0175.0415-004.87-splash10-00di-0900000000-e0c9778e979bc6b2247b | 6-Hydroxy-4-methylcoumarin, 6-Hydroxy-4-methyl-2-benzopyrone | Phenolic acids | 4.96 |
| p0-0193.0510-006.19-splash10-001i-0900000000-4a139520bb037f200169 | Isoferulic acid | Phenolic acids | 5.96 |
| p1-0153.0527-005.78-splash10-014i-9000000000-da2273c9bd6ce52aabe5 | Vanillin | Phenolic acids | 5.53 |
| p1-0165.0518-004.36-splash10-0006-9200000000-6459219a78606196622c | p-Coumaric acid | Phenolic acids | 4.41 |
| p1-0165.0520-005.79-splash10-00kf-9400000000-13ae8365c1a62d5c59fe | Coumaric acid | Phenolic acids | 5.81 |
| p1-0165.0587-006.04-splash10-00kf-9500000000-a488b5545cdaa2a58f92 | m-Coumaric acid | Phenolic acids | 6.05 |
| p1-0177.0521-011.32-splash10-0f89-2900000000-5ac25f150ad6e7ade4bd | 4-Methylumbelliferone | Phenolic acids | 11.24 |
| p1-0181.0490-004.46-splash10-000i-9700000000-e703e4943b64698cbddb | Caffeic acid | Phenolic acids | 4.32 |
| p1-0211.0963-010.05-splash10-0avi-3900000000-e1d65f6ebb41943119a6 | 3-Methyl-1-(2,4,6-trihydroxyphenyl)-1-butanone | Phenolic acids | 10.26 |
| p1-0301.0683-008.14-splash10-0udi-0449000000-4198c256fe99e6d02a19 | isokaempferide | Phenolic acids | 8.13 |
| p1-0339.1047-002.96-splash10-0002-0900000000-08e391df63fa66cb2b00 | Coumaroylquinic acid | Phenolic acids | 2.77 |
| p1-0339.1047-005.17-splash10-0002-0900000000-b5779bdd512da6446de4 | 3-O-p-Coumaroylquinic acid | Phenolic acids | 4.95 |
| p1-0344.1313-004.56-splash10-0002-0900000000-1be0c65d69dbb06baefd | trans-Melilotoside | Phenolic acids | 4.36 |
| p1-0403.0989-009.56-splash10-000f-0529000000-72226ad08ed77c804419 | 4-O-Demethyl-13-dihydroadriamycinone | Phenolic acids | 9.4 |
| p1-0653.1783-005.97-splash10-03di-0229300000-016ce1456beb9a334265 | Tricin 7-[rhamnosyl-(1->2)-galacturonide] | Phenolic acids | 5.79 |
| p1-0372.1261-005.00-splash10-03di-0900000000-d5b3a307fc74cb60b682 | Chlorogenic acid | Phenolic acids | 4.8 |
| p1-0744.0736-000.75-splash10-0udi-0010239000-5499bee0dc1eb47d8fce | NADP | Phenolic acids | 0.74 |
| p1-0409.1549-004.59-splash10-0596-0149200000-886b1a0080f8994080ed | Ginkgolide A | Phenolic acids | 4.4 |
| p1-0263.0553-005.19-splash10-0a59-0900000000-a974c303ef8408fd1fa9 | Daphnetin Diacetate | Phenolic acids | 4.98 |
| p1-0425.3740-013.76-splash10-05p2-1940100000-3260258466717bc83559 | alpha-Amyrone | Phenolic acids | 13.54 |
| p1-0243.0644-004.89-splash10-00kb-0910000000-cf22130fae53f4db63fa | 2,4,5,6-Phenanthrenetetrol | Phenolic acids | 4.72 |
| p0-0175.0256-004.46-splash10-00kr-9400000000-4fe93c270aad4cfcfeb8 | D-Glucurono-6,3-lactone | Phenolic acids | 4.27 |
| p0-0184.9873-000.67-splash10-004i-9000000000-6ca896804333d49b905d | 2-Phospho-D-glyceric acid | Phenolic acids | 0.68 |
| p0-0193.0364-000.66-splash10-0a4i-9100000000-2e97c3c7b24da02d64b9 | 2-keto-D-Gluconic acid | Phenolic acids | 0.64 |
| p0-0193.0417-004.32-splash10-001i-0900000000-1090dbe7c72a0084c0cc | trans-Ferulic acid | Phenolic acids | 4.11 |
| p0-0193.0726-001.14-splash10-0ab9-9100000000-d6a40d6be107d1290b5c | Methyl-beta-galactopyranoside | Phenolic acids | 1.09 |
| p0-0218.1055-003.26-splash10-0072-9400000000-e8928321bbdb45b8ef87 | Pantothenic acid | Phenolic acids | 3.05 |
| p0-0365.0923-006.26-splash10-0udi-0190000000-533fd672d438561bfabc | 1-hydroxy-4-[(2S,3R,4S,5S,6R)-3,4,5-trihydroxy-6-(hydroxymethyl)oxan-2-yl]oxynaphthalene-2-carboxylic acid | Phenolic acids | 6.09 |
| p0-0401.1113-004.31-splash10-0006-0900000000-7a5e865ba3ebab00c2fb | (E)-3-[4-methoxy-2-[(2S,3R,4S,5S,6R)-3,4,5-trihydroxy-6-(hydroxymethyl)oxan-2-yl]oxyphenyl]prop-2-enoic acid | Phenolic acids | 4.11 |
| p0-0507.1170-006.28-splash10-00di-0190000000-2e5445d017b4fd2e9d05 | [(2R,3S,4S,5R,6R)-6-[(5,8-dihydroxy-4-oxo-2,3-dihydro-1H-naphthalen-1-yl)oxy]-3,4,5-trihydroxyoxan-2-yl]methyl 3,4,5-trihydroxybenzoate | Phenolic acids | 6.1 |
| p0-0533.1603-007.80-splash10-00lr-0692070000-220e61f28299aaf663a7 | Cyclopenta[c]pyran-4-carboxylic acid, 1,4a,5,7a-tetrahydro-7-(hydroxymethyl)-1-[[6-O-[(2E)-3-(4-hydroxyphenyl)-1-oxo-2-propen-1-yl]-beta-D-glucopyranosyl]oxy]-, methyl ester | Phenolic acids | 7.78 |
| p1-0107.0854-000.18-splash10-014l-9000000000-41bd4972412407364678 | m-Xylene | Phenolic acids | 2.99 |
| p1-0130.0867-001.19-splash10-001i-9000000000-a666288471279640e512 | NIPECOTIC ACID | Phenolic acids | 1.12 |
| p1-0133.0633-005.56-splash10-004i-9300000000-0a96619fd7f0d4191201 | Indanone | Phenolic acids | 5.79 |
| p1-0137.0582-007.34-splash10-00di-4900000000-40c83b02e79c10a5991f | 2'-Hydroxyacetophenone | Phenolic acids | 7.22 |
| p1-0137.0597-003.48-splash10-00kf-9000000000-493ada1ab27ccb6c20f8 | M-toluic Acid | Phenolic acids | 3.27 |
| p1-0149.0216-012.18-splash10-006x-9600000000-203af040c1f1e75b93d5 | Phthalic anhydride | Phenolic acids | 12.12 |
| p1-0155.0345-003.90-splash10-000i-5900000000-1e85c01aa3be8fa31d4d | Gentisic acid | Phenolic acids | 3.7 |
| p1-0163.0389-003.80-splash10-000i-9300000000-5d6147a1f87de4f64b9e | 2-Oxo-5-methylthiopentanoic acid | Phenolic acids | 3.77 |
| p1-0163.0752-007.25-splash10-000i-9300000000-48d9ffb40dd70c7bd06a | 8Z-Decene-4,6-diynoic acid | Phenolic acids | 7.01 |
| p1-0164.0405-003.73-splash10-0006-9300000000-751f8eb12a284303f78e | 1H-Benzotriazole-4-carboxylic acid (Tentative) | Phenolic acids | 3.53 |
| p1-0171.0254-002.42-splash10-0a59-9600000000-9490f45149a6a459898f | Thiourocanic acid | Phenolic acids | 2.52 |
| p1-0179.0680-005.28-splash10-00lu-1900000000-b0ffdf21110711b61908 | 10-hydroxy-8E-Decene-4,6-diynoic acid | Phenolic acids | 5.32 |
| p1-0187.0766-008.46-splash10-0006-0900000000-7377ffd1daf328aa015e | 3-Phenoxyphenol | Phenolic acids | 8.74 |
| p1-0195.0639-008.33-splash10-00ks-2900000000-06b9f6a0e62a72c543c4 | Methyl caffeate | Phenolic acids | 8.19 |
| p1-0195.0648-008.89-splash10-03di-2900000000-bfe8fd3613c3d143701f | Dimethyl-phthalate | Phenolic acids | 8.69 |
| p1-0207.0627-008.48-splash10-014m-4900000000-b4930f3251c704fb9543 | Citropten | Phenolic acids | 8.44 |
| p1-0213.1831-005.47-splash10-05fs-7900000000-03297ebbc2f51968940c | delta-Tridecalactone | Phenolic acids | 5.38 |
| p1-0225.0744-004.96-splash10-00mp-1900000000-ad731446ee466807ae08 | Sinapic acid | Phenolic acids | 4.96 |
| p1-0239.0890-008.63-splash10-00mn-1900000000-06ffc386a267dd52139a | 3,4,5-Trimethoxycinnamic acid | Phenolic acids | 8.44 |
| p1-0247.1286-004.08-splash10-0zmi-0900000000-a3a051de82818791e464 | Pentaleno[1,6a-c]pyran-9-carboxylic acid, 1,3,4,5,6,7,7a,9a-octahydro-4,6,6-trimethyl-3-oxo-, (4S,4aR,7aS,9aR)- | Phenolic acids | 3.94 |
| p1-0261.2210-011.78-splash10-0a59-3900000000-e9733f9750aeb6b71c55 | 6-[5]-ladderane-1-hexanol | Phenolic acids | 11.75 |
| p1-0271.0943-011.53-splash10-00di-0930000000-01258ad17f46022e3151 | beta-Zearalenol | Phenolic acids | 11.45 |
| p1-0279.2415-011.28-splash10-0002-0920000000-d1ffedb302bef0c03519 | Pinolenic Acid | Phenolic acids | 11.76 |
| p1-0287.0885-009.62-splash10-00fv-0980000000-5008c2b7bab1faaf4f14 | (9-hydroxy-8,8-dimethyl-2-oxo-9,10-dihydropyrano[2,3-f]chromen-10-yl) acetate | Phenolic acids | 9.44 |
| p1-0287.0891-006.94-splash10-000i-0890000000-883761026a5fe6c24e03 | 5-hydroxy-2-(4-hydroxyphenyl)-7-methoxy-2,3-dihydrochromen-4-one | Phenolic acids | 6.99 |
| p1-0291.1201-005.51-splash10-0h01-0920000000-9c2e4390da4bd3fe82bb | 2H-3-Benzoxacyclododecin-2,10(1H)-dione, 4,5,6,7,8,9-hexahydro-8,11,13-trihydroxy-4-methyl- | Phenolic acids | 5.45 |
| p1-0293.2463-013.62-splash10-00ls-9600000000-9c5fb8f847ad820fb62f | 9(Z),11(E),13(E)-Octadecatrienoic Acid methyl ester | Phenolic acids | 13.5 |
| p1-0303.0472-005.29-splash10-000i-0490000000-1025262b1bf1644f087b | (2Z)-4,6-dihydroxy-2-[(3,4,5-trihydroxyphenyl)methylidene]-1-benzofuran-3-one | Phenolic acids | 5.3 |
| p1-0327.0054-011.38-splash10-0002-9000000000-1a6a8f5f725f0619eef1 | Tris(1-chloro-2-propyl)phosphate | Phenolic acids | 11.34 |
| p1-0333.0785-005.97-splash10-0udi-0900000000-5be4ca401b93bc55cf40 | beta-Glucogallin | Phenolic acids | 5.79 |
| p1-0335.2572-013.76-splash10-00sj-6910000000-1c25db51c6fe627198a0 | 21-hydroxyallopregnanolone | Phenolic acids | 13.91 |
| p1-0335.2573-012.45-splash10-00l5-9400000000-1ca5c1e1b537574bfe07 | (±)5(6)-EET methyl ester | Phenolic acids | 12.16 |
| p1-0343.1006-004.48-splash10-03di-0900000000-2d662f605d59201521ad | 1-O-Caffeoylglucose | Phenolic acids | 7.56 |
| p1-0343.1036-003.86-splash10-03di-0900000000-6af0ca4e2bec6af17b8f | Glucocaffeic acid | Phenolic acids | 3.75 |
| p1-0355.0988-003.32-splash10-000i-0900000000-53e7ce5ca67bb4a13f66 | (3R,5R)-4-[(E)-3-(3,4-dihydroxyphenyl)prop-2-enoyl]oxy-1,3,5-trihydroxycyclohexane-1-carboxylic acid | Phenolic acids | 3.13 |
| p1-0355.1267-004.92-splash10-01t9-0900000000-1e52c7fda78b6ed56560 | Ambonone | Phenolic acids | 4.74 |
| p1-0355.2861-011.67-splash10-01ot-5931000000-5f50e124b3aaf7779180 | 2-Linoleoyl Glycerol | Phenolic acids | 11.66 |
| p1-0357.1150-007.99-splash10-004j-0900000000-c1339b182ea30417c838 | 1-O-Feruloyl-β-D-glucose | Phenolic acids | 7.83 |
| p1-0387.1259-008.24-splash10-0a6r-0972000000-00aba9617b1a7a99d2b6 | 1-O-Sinapoyl-β-D-glucose | Phenolic acids | 8.1 |
| p1-0399.2477-012.07-splash10-052b-5910000000-3857766d95f05e8858de | Tri(butoxyethyl)phosphate | Phenolic acids | 12.03 |
| p1-0433.1082-008.87-splash10-0udi-0009000000-62d7f06daf5e977b248f | Peonidin-3-O-alpha-arabinopyranoside | Phenolic acids | 8.71 |
| p1-0435.0901-004.40-splash10-0udi-0009000000-6d4085cd07186f3150d7 | avicularin | Phenolic acids | 4.18 |
| p1-0479.1219-008.52-splash10-014i-0019100000-0f48628d9f2d15481955 | 6-Hydroxykaempferol 4'-methyl ether 7-glucoside | Phenolic acids | 8.61 |
| p1-0406.2404-005.08-splash10-0avi-0920000000-f421f8d5fd446af1ba4c | Dihydroroseoside | Phenolic acids | 4.95 |
| p1-0154.0846-001.61-splash10-00kf-9300000000-41755174db0148743f4a | p-Octopamine | Phenylpropanoids | 1.32 |
| p1-0167.0315-005.68-splash10-001i-9400000000-03400a8d5450d7141ca5 | 3,4-Dihydroxymandelic acid | Phenylpropanoids | 5.47 |
| p1-0211.0939-006.91-splash10-00dj-4900000000-52fbbd817397e03984a3 | 2-Methoxy-3-(4-methoxyphenyl)propanoic acid | Phenylpropanoids | 6.63 |
| p1-0251.1987-011.75-splash10-00l2-9700000000-495aea36ad82b1fd27ed | Norambreinolide | Phenylpropanoids | 11.53 |
| p1-0321.2740-011.38-splash10-05u2-5910000000-0fde33749ca5096da184 | 5(Z),8(Z),11(Z)-Eicosatrienoic Acid methyl ester | Phenylpropanoids | 11.36 |
| p1-0378.0653-010.91-splash10-03di-0029000000-84ec5e3de60f29896478 | 4-(2-Chlorophenyl)-5-methoxycabonyl-3-ethoxycarbonyl-6-methylpicolinic acid | Phenylpropanoids | 10.71 |
| p0-0219.0301-007.04-splash10-044i-0900000000-93154b3b04a2591db8b5 | PURPUROGALLIN | Phenylpropanoids | 6.89 |
| p1-0321.0941-002.96-splash10-00ds-2900000000-83712fc00cc40df6407a | Ourateacatechin | Phenylpropanoids | 2.77 |
| p1-0135.1189-006.49-splash10-002f-9100000000-513eb070c2982e658d9b | p-Mentha-1,3,8-triene | Prenol lipids | 6.38 |
| p1-0151.1109-005.08-splash10-05r3-9100000000-314a6d816145fedabb14 | Piperitenone | Prenol lipids | 4.96 |
| p1-0181.0562-005.75-splash10-00kf-9300000000-4e53883c00d32485c234 | Vanillylmandelic acid | Prenol lipids | 5.56 |
| p1-0191.1427-006.77-splash10-05o3-3900000000-b64edb67e291ebfc8cdf | xi-3-(4-Isopropylphenyl)-2-methylpropanal | Prenol lipids | 6.38 |
| p1-0193.1565-006.19-splash10-0159-3900000000-bef40699ce202c24ae3f | 2,4-Diisopropyl-5-methylphenol | Prenol lipids | 6.08 |
| p1-0211.1643-007.43-splash10-0aor-5900000000-59baa41bc63b92a812e1 | 3E,5E-tridecadienoic acid | Prenol lipids | 6.91 |
| p1-0221.1892-011.09-splash10-0a59-4900000000-4b1fc256d731b6a38417 | Caryophyllene alpha-oxide | Prenol lipids | 11.18 |
| p1-0263.2277-011.59-splash10-00lr-9510000000-44914c4e4c092f6294ed | 6,10,14-Trimethyl-5,9,13-pentadecatrien-2-one | Prenol lipids | 11.66 |
| p1-0283.2628-011.43-splash10-0ldi-9420000000-f4f2b88f80a108a5bb0b | Petroselinic acid | Prenol lipids | 11.71 |
| p1-0310.1471-002.85-splash10-03dr-5900000000-71b4f97ebb175565868b | (3R)-4,4-Dimethyl-2-oxotetrahydro-3-furanyl beta-D-glucopyranoside | Prenol lipids | 2.68 |
| p1-0291.2286-012.26-splash10-002f-7900000000-65e9fa86f404da2fd277 | Epiandrosterone | Prenol lipids | 12.2 |
| p0-0533.3457-011.33-splash10-000i-0002910000-c62911b9daaaeaa95451 | Asiatic acid | Prenol lipids | 10.95 |
| p1-0325.1138-000.73-splash10-004s-5900000000-08acb0f988ad635e7a45 | Kojibiose | Quinones | 0.71 |
| p1-0133.1003-005.34-splash10-00mo-9500000000-e6bcbdd7ae074457be41 | Tetralin | Quinones | 5.32 |
| p1-0145.0639-004.98-splash10-00or-1900000000-2ea8d36bed3d3f3fc60a | 2-Naphthol | Quinones | 4.81 |
| p1-0191.0321-007.27-splash10-00di-2900000000-c935ae23f5d0ea297801 | 3,5-Dihydroxy-1,4-naphthoquinone | Quinones | 7.05 |
| p1-0207.0629-005.19-splash10-03di-1900000000-7473bba23e48ab87abde | cis-1,2-Dihydroxy-1,2-dihydro-8-carboxynaphthalene | Quinones | 4.98 |
| p1-0339.1039-007.44-splash10-004j-0941000000-f3cf174834e211e90526 | beta-D-Glucopyranoside, 4,8-dihydroxy-1-naphthalenyl | Quinones | 8.37 |
| p0-0183.0311-004.53-splash10-00di-1900000000-8a2b51f6b622c82745f5 | Methylgallate | Tannins | 4.29 |
| p0-0299.0222-005.58-splash10-03fr-0890000000-840f9f866b7b394f8d78 | EMODIC ACID | Tannins | 5.35 |
| p1-0285.0726-008.37-splash10-004j-1950000000-91435c212d009904d50c | BRAZILEIN | Tannins | 8.36 |
| p1-0407.3626-012.90-splash10-0a4i-1941100000-9a446b6412dff59a150d | 4,4'-Diapophytofluene | Terpenoids | 12.79 |
| p1-0135.1150-013.05-splash10-056u-9300000000-0675d61ed0c5e83e8e02 | (S)-p-Mentha-1,8-dien-7-ol | Terpenoids | 13.27 |
| p1-0397.3832-013.42-splash10-0002-1941000000-26c9fd2f034f06b7f9bb | beta-Sitosterol | Terpenoids | 13.49 |
| p1-0443.3824-012.72-splash10-0006-0640900000-86f3ac726795944f34f2 | Uvaol | Terpenoids | 12.63 |
| p1-0597.1391-004.90-splash10-0udi-0009110000-33df34c5ad95b696c44b | Peltatoside | Terpenoids | 4.73 |
| p0-0175.0276-003.35-splash10-000i-9100000000-aebac0d8020edef982db | Ascorbic acid | Vitamins and derivatives | 3.23 |
| Cya-3-O-(6-O-malonyl)-glu | Cyanidin 3-O-(6-O-malonyl-beta-D-glucoside) | Anthocyanins | 8.31 |
| Cya-3-O-ara | Cyanidin 3-O-arabinoside | Anthocyanins | 6.1 |
| Cya-3-O-gal | Cyanidin 3-O-galactoside | Anthocyanins | 5.12 |
| Cya-3-O-glu | Cyanidin 3-O-glucoside | Anthocyanins | 5.68 |
| Cya-3-O-rut | Cyanidin 3-O-rutinoside | Anthocyanins | 6.28 |
| Del-3,5-O-diglu | Delphinidin 3,5-O-diglucoside(Delphin) | Anthocyanins | 3.35 |
| Del-3-O-(6-O-malonyl)-glu | Delphinidin 3-O-(6''-O-malonyl)-beta-D-glucoside | Anthocyanins | 7.25 |
| Del-3-O-ara | Delphinidin 3-O-arabinoside | Anthocyanins | 5.21 |
| Del-3-O-gal | Delphinidin 3-O-galactoside | Anthocyanins | 4.17 |
| Del-3-O-glu | Delphinidin 3-O-glucoside | Anthocyanins | 4.68 |
| Del-3-O-rut | Delphinidin 3-O-rutinoside | Anthocyanins | 6.28 |
| Mal-3-O-(6-O-malonyl)-glu | Malvidin 3-O-(6-O-malonyl-beta-D-glucoside) | Anthocyanins | 10.12 |
| Mal-3-O-ara | Malvidin 3-O-arabinoside | Anthocyanins | 8.34 |
| Mal-3-O-gal | Malvidin 3-O-galactoside | Anthocyanins | 7.42 |
| Mal-3-O-glu | Malvidin 3-O-glucoside | Anthocyanins | 7.86 |
| Pel-3-O-(6-O-malonyl)-glu | Pelargonidin 3-O-(6-O-malonyl-beta-D-glucoside) | Anthocyanins | 9.28 |
| Pel-3-O-ara | Pelargonidin 3-O-arabinoside | Anthocyanins | 6.97 |
| Pel-3-O-glu | Pelargonidin 3-O-glucoside | Anthocyanins | 6.64 |
| Peo-3-O-(6-O-malonyl)-glu | Peonidin 3-O-(6-O-malonyl-beta-D-glucoside) | Anthocyanins | 9.81 |
| Peo-3-O-ara | Peonidin 3-O-arabinoside | Anthocyanins | 7.77 |
| Peo-3-O-gal | Peonidin 3-O-galactoside | Anthocyanins | 6.81 |
| Peo-3-O-glu | Peonidin 3-O-glucoside | Anthocyanins | 7.36 |
| Pet-3-O-(6-O-malonyl)-glu | Petunidin 3-O-(6-O-malonyl-beta-D-glucoside) | Anthocyanins | 8.86 |
| Pet-3-O-ara | Petunidin 3-O-arabinoside | Anthocyanins | 6.9 |
| Pet-3-O-gal | Petunidin 3-O-galactoside | Anthocyanins | 5.93 |
| Pet-3-O-glu | Petunidin 3-O-glucoside | Anthocyanins | 6.43 |
| Procyanidin B1 | Procyanidin B1 | Procyanidins | 2.11 |
| Procyanidin B2 | Procyanidin B2 | Procyanidins | 3.53 |
| Procyanidin C1 | Procyanidin C1 | Procyanidins | 4.66 |
| Procyanidin B3 | Procyanidin B3 | Procyanidins | 1.91 |

**Table S4.** All the metabolites in SR vs. SG.

| ID | SR_1_1 | SR_1_2 | SR_1_3 | SG_1_1 | SG_1_2 | SG_1_3 | SR_2_1 | SR_2_2 | SR_2_3 | SG_2_1 | SG_2_2 | SG_2_3 | SR_3_1 | SR_3_2 | SR_3_3 | SG_3_1 | SG_3_2 | SG_3_3 |
| --- | --- | --- | --- | --- | --- | --- | --- | --- | --- | --- | --- | --- | --- | --- | --- | --- | --- | --- |
| NADP | 1.33E-06 | 2.60E-06 | 1.24E-06 | 3.48E-06 | 2.44E-06 | 1.49E-06 | 1.02E-06 | 3.83E-06 | 2.96E-06 | 3.55E-06 | 2.18E-06 | 2.35E-06 | 1.40E-06 | 2.39E-06 | 1.91E-06 | 1.43E-06 | 1.95E-06 | 2.32E-06 |
| 4-METHYLDAPHNETIN | 3.15E-06 | 4.57E-06 | 2.49E-06 | 6.07E-06 | 3.28E-06 | 9.82E-06 | 4.48E-06 | 5.71E-06 | 1.30E-05 | 1.27E-05 | 5.90E-06 | 6.72E-06 | 1.07E-14 | 5.87E-06 | 5.45E-06 | 4.78E-06 | 6.71E-06 | 8.34E-06 |
| L-Phenylalanine | 8.81E-15 | 1.89E-06 | 1.19E-14 | 1.44E-14 | 3.46E-06 | 1.39E-14 | 5.38E-06 | 9.69E-15 | 2.62E-06 | 1.12E-14 | 1.31E-14 | 6.51E-06 | 1.07E-14 | 1.03E-06 | 1.76E-06 | 2.12E-06 | 1.26E-14 | 2.28E-06 |
| (Z)-Resveratrol | 4.40E-06 | 1.02E-14 | 7.20E-06 | 7.76E-06 | 1.36E-05 | 1.39E-14 | 5.31E-06 | 7.97E-06 | 1.56E-05 | 4.00E-06 | 4.20E-06 | 7.38E-06 | 7.53E-06 | 6.90E-06 | 9.34E-06 | 1.30E-14 | 9.03E-06 | 1.30E-14 |
| Quinolone | 8.23E-07 | 1.64E-06 | 4.86E-06 | 6.09E-06 | 9.17E-06 | 9.20E-06 | 8.41E-06 | 1.63E-06 | 9.18E-06 | 3.90E-06 | 2.67E-06 | 6.55E-06 | 3.35E-06 | 5.40E-06 | 2.58E-06 | 5.67E-06 | 2.72E-06 | 1.77E-06 |
| NCGC00385845-01 | 2.63E-06 | 1.08E-05 | 5.21E-06 | 1.72E-05 | 1.64E-05 | 5.84E-06 | 7.04E-06 | 9.69E-15 | 1.12E-05 | 3.17E-06 | 1.31E-14 | 6.43E-06 | 4.49E-06 | 3.48E-06 | 8.54E-06 | 6.52E-06 | 7.28E-06 | 7.57E-06 |
| 5(Z),8(Z),11(Z)-Eicosatrienoic Acid methyl ester | 5.93E-06 | 3.79E-06 | 1.19E-05 | 1.45E-05 | 4.51E-06 | 9.93E-06 | 4.36E-06 | 3.38E-06 | 4.33E-06 | 3.05E-06 | 6.29E-06 | 5.08E-06 | 3.69E-06 | 9.35E-06 | 6.89E-06 | 5.56E-06 | 4.36E-06 | 4.54E-06 |
| Glutathione (oxidized) | 8.81E-15 | 1.02E-14 | 6.51E-06 | 1.06E-05 | 5.59E-06 | 7.78E-06 | 7.27E-06 | 5.89E-06 | 4.64E-06 | 8.36E-06 | 7.43E-06 | 5.00E-06 | 1.30E-05 | 4.69E-06 | 1.85E-05 | 6.07E-06 | 1.26E-14 | 8.28E-06 |
| CAY10487 | 1.15E-05 | 8.85E-06 | 3.91E-06 | 1.44E-14 | 3.86E-06 | 3.55E-06 | 2.78E-06 | 9.00E-06 | 1.06E-14 | 5.81E-06 | 5.63E-06 | 4.55E-06 | 5.62E-06 | 1.00E-14 | 3.93E-06 | 1.30E-14 | 6.78E-06 | 8.70E-06 |
| Norambreinolide | 4.94E-06 | 1.49E-05 | 7.04E-06 | 1.17E-05 | 1.32E-14 | 1.39E-14 | 8.06E-06 | 5.38E-06 | 7.04E-06 | 9.73E-06 | 1.14E-05 | 9.84E-06 | 6.33E-06 | 6.42E-06 | 9.43E-15 | 1.30E-14 | 8.99E-06 | 2.43E-05 |
| Rhodalidin | 3.41E-06 | 3.47E-06 | 5.87E-06 | 9.93E-06 | 8.83E-06 | 9.04E-06 | 8.32E-06 | 5.97E-06 | 7.54E-06 | 3.29E-06 | 5.14E-06 | 1.46E-05 | 7.29E-06 | 3.19E-06 | 3.71E-06 | 4.79E-06 | 4.83E-06 | 7.75E-06 |
| 2-(3,4-dihydroxyphenyl)-3,5-dihydroxy-7-methoxy-4H-chromen-4-one | 2.78E-06 | 2.18E-06 | 3.28E-06 | 8.36E-06 | 7.48E-06 | 9.27E-06 | 1.02E-05 | 2.88E-06 | 7.55E-06 | 8.51E-06 | 8.79E-06 | 9.55E-06 | 1.18E-05 | 5.80E-06 | 4.67E-06 | 3.58E-06 | 2.69E-06 | 2.67E-06 |
| Xanthine | 7.30E-06 | 8.25E-06 | 5.17E-06 | 5.62E-06 | 2.80E-06 | 5.43E-06 | 3.36E-06 | 8.10E-06 | 4.26E-06 | 3.96E-06 | 8.39E-06 | 8.17E-06 | 2.96E-06 | 3.36E-06 | 3.92E-06 | 6.05E-06 | 6.79E-06 | 8.24E-06 |
| Ourateacatechin | 5.66E-06 | 6.68E-06 | 7.47E-06 | 1.19E-05 | 1.08E-05 | 1.55E-05 | 1.17E-14 | 3.79E-06 | 8.35E-06 | 4.59E-06 | 6.10E-06 | 6.71E-06 | 4.27E-06 | 2.58E-06 | 2.94E-06 | 9.92E-06 | 5.55E-06 | 4.32E-06 |
| Quercetin-3,4'-O-di-beta-glucopyranoside | 1.45E-06 | 2.67E-06 | 5.59E-06 | 3.91E-06 | 1.83E-05 | 5.86E-06 | 7.82E-06 | 5.39E-06 | 9.99E-06 | 1.00E-05 | 2.89E-05 | 1.29E-05 | 9.19E-06 | 9.65E-06 | 3.86E-06 | 5.94E-06 | 3.49E-06 | 7.94E-06 |
| Tricin 7-[rhamnosyl-(1->2)-galacturonide] | 2.07E-05 | 1.55E-05 | 1.47E-05 | 3.56E-06 | 8.44E-06 | 1.39E-14 | 6.36E-06 | 3.56E-06 | 7.05E-06 | 1.30E-05 | 1.06E-05 | 7.30E-06 | 5.82E-06 | 1.02E-05 | 1.21E-05 | 1.01E-05 | 5.31E-06 | 1.57E-05 |
| Kinurenine | 3.60E-06 | 7.27E-06 | 4.24E-06 | 1.06E-05 | 4.55E-06 | 4.03E-06 | 3.21E-06 | 5.92E-06 | 3.59E-06 | 6.05E-06 | 6.85E-06 | 5.27E-06 | 5.28E-06 | 3.78E-06 | 5.27E-06 | 5.49E-06 | 5.92E-06 | 8.13E-06 |
| 5,7-dihydroxy-2-(4-hydroxy-3-methoxyphenyl)-3-{[3,4,5-trihydroxy-6-(hydroxymethyl)oxan-2-yl]oxy}-4H-chromen-4-one | 5.77E-06 | 4.79E-06 | 2.96E-05 | 2.97E-06 | 2.14E-06 | 2.85E-06 | 2.70E-06 | 1.56E-05 | 2.29E-06 | 3.89E-06 | 1.51E-05 | 3.06E-06 | 2.41E-06 | 3.91E-06 | 2.22E-06 | 8.08E-06 | 9.44E-06 | 1.97E-06 |
| PURPUROGALLIN | 8.81E-15 | 6.81E-06 | 8.57E-06 | 1.71E-05 | 1.53E-05 | 1.22E-05 | 1.04E-05 | 6.64E-06 | 1.22E-05 | 8.03E-06 | 1.22E-05 | 1.08E-05 | 1.32E-05 | 7.17E-06 | 8.61E-06 | 7.87E-06 | 7.73E-06 | 1.55E-05 |
| Purpurin | 9.06E-06 | 1.16E-05 | 5.81E-06 | 1.33E-05 | 1.06E-05 | 1.02E-05 | 1.60E-05 | 1.46E-05 | 1.68E-05 | 1.07E-05 | 1.82E-05 | 1.22E-05 | 1.87E-05 | 1.78E-05 | 8.01E-06 | 1.03E-05 | 1.09E-05 | 9.72E-06 |
| [6-[5,7-dihydroxy-2-(4-hydroxyphenyl)-4-oxochromen-3-yl]oxy-3,4,5-trihydroxyoxan-2-yl]methyl 3,4,5-trihydroxybenzoate | 1.25E-05 | 1.47E-05 | 1.12E-05 | 1.26E-05 | 1.02E-05 | 1.79E-05 | 7.53E-06 | 5.05E-06 | 7.49E-06 | 1.98E-05 | 1.50E-05 | 2.79E-05 | 3.89E-06 | 8.42E-06 | 3.12E-06 | 2.00E-05 | 9.29E-06 | 2.06E-05 |
| Cassiamin C | 5.93E-06 | 8.12E-06 | 1.68E-05 | 1.39E-05 | 7.89E-06 | 7.22E-06 | 2.20E-05 | 1.31E-05 | 2.94E-05 | 2.48E-05 | 1.33E-05 | 2.08E-05 | 1.34E-05 | 1.74E-05 | 9.43E-15 | 6.79E-06 | 1.41E-05 | 2.28E-05 |
| PGF1伪 Alcohol | 9.17E-06 | 1.13E-05 | 1.68E-05 | 1.50E-05 | 1.48E-05 | 2.22E-05 | 1.15E-05 | 9.69E-15 | 2.54E-06 | 1.10E-05 | 1.52E-05 | 1.98E-05 | 1.49E-05 | 1.18E-05 | 9.71E-06 | 6.38E-06 | 1.26E-14 | 1.18E-05 |
| 4,4'-Diapophytofluene | 1.08E-05 | 4.00E-06 | 8.51E-06 | 9.76E-06 | 1.01E-05 | 5.23E-06 | 1.06E-05 | 8.48E-06 | 4.53E-06 | 2.51E-05 | 8.20E-06 | 9.00E-06 | 2.68E-05 | 8.40E-06 | 1.36E-05 | 2.45E-05 | 1.42E-05 | 3.28E-05 |
| 4-{[(3S,4R,5S)-3-Hydroxy-5-(4-hydroxy-3-methoxyphenyl)-4-(hydroxymethyl)tetrahydro-3-furanyl]methyl}-2-methoxyphenyl beta-D-glucopyranoside | 1.10E-05 | 2.26E-05 | 2.99E-05 | 1.99E-05 | 1.10E-05 | 5.48E-06 | 4.80E-05 | 1.70E-05 | 1.74E-05 | 1.74E-05 | 1.75E-05 | 1.73E-05 | 2.50E-05 | 2.43E-05 | 1.72E-05 | 1.51E-05 | 6.00E-05 | 1.36E-05 |
| Protopanaxadiol | 9.42E-06 | 4.16E-06 | 1.03E-05 | 7.56E-06 | 1.25E-05 | 7.61E-06 | 1.40E-05 | 8.47E-06 | 6.51E-06 | 1.65E-05 | 2.32E-05 | 1.37E-05 | 3.48E-05 | 8.73E-06 | 1.20E-05 | 6.43E-06 | 6.58E-06 | 9.54E-06 |
| Xanthosine | 1.00E-05 | 1.26E-05 | 8.29E-06 | 1.25E-05 | 8.10E-06 | 3.72E-06 | 9.98E-06 | 2.47E-05 | 9.28E-06 | 1.88E-05 | 2.41E-05 | 1.46E-05 | 1.13E-05 | 6.75E-06 | 1.82E-05 | 1.35E-05 | 1.52E-05 | 3.31E-05 |
| 2-Methoxy-3-(4-methoxyphenyl)propanoic acid | 4.23E-06 | 4.40E-06 | 7.56E-06 | 2.59E-05 | 3.41E-05 | 7.43E-05 | 2.16E-05 | 8.53E-06 | 2.04E-05 | 9.18E-06 | 1.15E-05 | 1.89E-05 | 6.69E-06 | 2.74E-05 | 8.52E-06 | 2.47E-05 | 1.22E-05 | 1.02E-05 |
| alpha-Amyrone | 5.05E-06 | 1.88E-06 | 7.54E-06 | 4.37E-06 | 7.30E-06 | 4.55E-06 | 1.17E-14 | 1.08E-05 | 5.72E-06 | 1.96E-05 | 1.03E-05 | 1.08E-05 | 3.18E-05 | 9.46E-06 | 1.51E-05 | 3.57E-05 | 1.08E-05 | 3.32E-05 |
| Asiatic acid | 1.00E-05 | 1.00E-05 | 4.99E-06 | 1.50E-05 | 1.65E-05 | 1.55E-05 | 6.65E-06 | 1.14E-05 | 1.18E-05 | 1.24E-05 | 5.51E-06 | 1.46E-05 | 1.12E-05 | 1.06E-05 | 1.16E-05 | 8.54E-06 | 1.29E-05 | 8.16E-06 |
| 3,5,6,7,2',3',4'-Heptahydroxyflavone | 9.30E-06 | 3.03E-06 | 7.94E-06 | 1.84E-05 | 3.92E-05 | 1.15E-05 | 3.21E-05 | 1.37E-05 | 5.10E-05 | 1.38E-05 | 2.34E-06 | 2.87E-05 | 3.41E-05 | 2.21E-05 | 4.37E-05 | 1.53E-05 | 1.57E-05 | 1.97E-05 |
| 3,4-Dihydroxybenzaldehyde | 1.62E-05 | 1.84E-05 | 2.52E-05 | 2.22E-05 | 1.88E-05 | 2.00E-05 | 1.54E-05 | 9.66E-06 | 1.18E-05 | 8.46E-06 | 2.00E-05 | 1.24E-05 | 1.61E-05 | 1.46E-05 | 7.76E-06 | 5.42E-06 | 1.18E-05 | 7.15E-06 |
| Dimethyl-phthalate | 3.74E-06 | 1.11E-05 | 1.59E-05 | 2.50E-05 | 1.18E-05 | 1.45E-05 | 9.53E-06 | 1.01E-05 | 6.98E-06 | 1.03E-05 | 8.49E-06 | 1.34E-05 | 8.15E-06 | 7.77E-06 | 4.94E-06 | 9.69E-06 | 6.83E-06 | 8.08E-06 |
| Furo[4,3,2-ij]pyrano[4',3':4,5]cyclohepta[1,2-f][2]benzopyran-1,5,9,13(2H,11H)-tetrone, 3a,5a,6,8,12,13a,13b,13c-octahydro-7-(hydroxymethyl)-2,5a,11,11,13a-pentamethyl- | 4.34E-06 | 8.71E-06 | 1.32E-05 | 1.34E-05 | 1.70E-05 | 2.79E-05 | 2.59E-05 | 1.38E-05 | 1.24E-05 | 1.77E-05 | 6.23E-06 | 1.46E-05 | 1.22E-05 | 5.40E-06 | 9.42E-06 | 2.09E-05 | 7.45E-06 | 8.29E-06 |
| 4-(2-Chlorophenyl)-5-methoxycabonyl-3-ethoxycarbonyl-6-methylpicolinic acid | 9.19E-06 | 8.10E-06 | 1.10E-05 | 1.81E-05 | 1.46E-05 | 1.15E-05 | 2.28E-05 | 1.16E-05 | 1.56E-05 | 1.12E-05 | 7.36E-06 | 1.71E-05 | 2.19E-05 | 2.75E-05 | 1.78E-05 | 1.43E-05 | 2.73E-05 | 1.06E-05 |
| Uracil | 8.89E-06 | 5.32E-06 | 1.29E-05 | 9.41E-06 | 1.19E-05 | 1.63E-05 | 1.04E-05 | 1.73E-05 | 3.24E-05 | 1.50E-05 | 7.09E-05 | 1.75E-05 | 3.29E-06 | 7.71E-06 | 7.50E-06 | 4.03E-05 | 7.56E-06 | 1.15E-05 |
| [Gallocatechin(4alpha->8)]2catechin | 1.13E-05 | 1.38E-05 | 2.52E-05 | 1.02E-05 | 3.18E-05 | 4.34E-05 | 2.20E-05 | 7.95E-06 | 1.84E-05 | 1.98E-05 | 1.02E-05 | 5.34E-05 | 5.72E-05 | 7.79E-05 | 1.93E-05 | 3.59E-05 | 2.30E-05 | 1.88E-05 |
| Verapamil metabolite D-617 | 6.19E-06 | 1.08E-05 | 1.02E-05 | 1.44E-05 | 1.18E-05 | 1.11E-05 | 1.45E-05 | 1.10E-05 | 1.76E-05 | 1.56E-05 | 1.80E-05 | 1.52E-05 | 1.01E-05 | 1.13E-05 | 1.31E-05 | 1.60E-05 | 1.97E-05 | 1.65E-05 |
| trans-Ferulic acid | 3.12E-06 | 1.86E-05 | 1.37E-05 | 1.93E-05 | 1.78E-05 | 1.76E-05 | 1.13E-05 | 3.18E-05 | 7.33E-06 | 2.16E-05 | 9.78E-06 | 1.21E-05 | 9.71E-06 | 7.25E-06 | 1.62E-05 | 1.53E-05 | 3.06E-05 | 4.38E-05 |
| Methylisoeugenol | 1.96E-05 | 5.41E-05 | 2.98E-05 | 1.54E-05 | 1.66E-05 | 1.34E-05 | 1.17E-14 | 4.18E-05 | 1.13E-05 | 2.93E-05 | 3.46E-05 | 3.07E-05 | 1.95E-05 | 2.72E-05 | 1.77E-05 | 1.30E-14 | 7.96E-05 | 2.55E-05 |
| NCGC00381407-01 | 4.60E-05 | 5.14E-05 | 2.47E-05 | 4.95E-05 | 1.55E-05 | 2.07E-05 | 1.15E-05 | 4.12E-05 | 6.53E-06 | 4.23E-05 | 2.91E-05 | 1.79E-05 | 1.99E-05 | 3.95E-06 | 1.91E-05 | 2.00E-05 | 4.21E-05 | 7.55E-05 |
| (9-hydroxy-8,8-dimethyl-2-oxo-9,10-dihydropyrano[2,3-f]chromen-10-yl) acetate | 7.38E-06 | 1.04E-05 | 1.03E-05 | 2.33E-05 | 1.80E-05 | 7.69E-06 | 1.23E-05 | 1.23E-05 | 2.19E-05 | 2.08E-05 | 1.56E-05 | 3.25E-05 | 1.31E-05 | 3.54E-05 | 1.50E-05 | 1.82E-05 | 3.13E-05 | 9.46E-06 |
| isokaempferide | 4.33E-06 | 4.94E-06 | 6.00E-06 | 3.16E-05 | 8.17E-06 | 2.88E-05 | 4.96E-05 | 9.90E-06 | 1.73E-05 | 4.13E-05 | 4.80E-05 | 3.16E-05 | 2.23E-05 | 1.14E-05 | 9.37E-06 | 3.20E-05 | 3.19E-05 | 3.34E-06 |
| 2H-3-Benzoxacyclododecin-2,10(1H)-dione, 4,5,6,7,8,9-hexahydro-8,11,13-trihydroxy-4-methyl- | 8.81E-15 | 5.03E-06 | 4.40E-06 | 6.18E-06 | 5.79E-06 | 7.42E-06 | 9.44E-06 | 7.23E-06 | 2.81E-06 | 1.39E-05 | 5.34E-06 | 2.73E-06 | 1.07E-14 | 4.84E-06 | 7.85E-06 | 7.73E-06 | 8.64E-06 | 8.44E-06 |
| CONESSINE | 1.37E-05 | 1.21E-05 | 1.25E-05 | 6.73E-06 | 7.72E-06 | 9.37E-06 | 2.76E-05 | 3.37E-05 | 3.25E-05 | 4.54E-05 | 2.32E-05 | 1.33E-14 | 1.86E-05 | 9.01E-06 | 1.36E-05 | 8.21E-05 | 1.15E-05 | 7.89E-05 |
| Caryophyllene alpha-oxide | 2.50E-05 | 1.59E-05 | 2.52E-05 | 2.95E-05 | 3.45E-05 | 3.15E-05 | 2.76E-05 | 1.35E-05 | 2.50E-05 | 2.39E-05 | 2.63E-05 | 2.96E-05 | 2.45E-05 | 1.94E-05 | 1.92E-05 | 3.09E-05 | 3.50E-05 | 3.27E-05 |
| 1-Naphthylamine | 1.08E-05 | 1.61E-05 | 2.63E-05 | 3.41E-05 | 3.44E-05 | 2.65E-05 | 1.65E-05 | 2.07E-05 | 2.42E-05 | 3.04E-05 | 1.76E-05 | 2.66E-05 | 1.84E-05 | 1.12E-05 | 3.82E-05 | 2.41E-05 | 2.56E-05 | 2.65E-05 |
| 7,4',5'-Trihydroxy-5,2'-oxido-4-phenylcoumarin | 5.15E-05 | 7.01E-05 | 5.70E-05 | 5.58E-05 | 5.58E-05 | 6.21E-05 | 3.37E-05 | 4.93E-05 | 2.76E-05 | 2.85E-05 | 5.21E-05 | 3.78E-05 | 1.73E-05 | 3.38E-05 | 2.01E-05 | 3.59E-05 | 2.52E-05 | 3.54E-05 |
| (卤)-Naringenin | 1.90E-05 | 2.22E-05 | 2.52E-05 | 2.05E-05 | 3.44E-05 | 2.53E-05 | 3.45E-05 | 1.91E-05 | 2.21E-05 | 4.04E-05 | 1.89E-05 | 3.06E-05 | 3.70E-05 | 3.18E-05 | 2.57E-05 | 3.12E-05 | 4.60E-05 | 3.26E-05 |
| Pentaleno[1,6a-c]pyran-9-carboxylic acid, 1,3,4,5,6,7,7a,9a-octahydro-4,6,6-trimethyl-3-oxo-, (4S,4aR,7aS,9aR)- | 2.69E-05 | 3.12E-05 | 3.36E-05 | 2.69E-05 | 2.19E-05 | 2.95E-05 | 1.48E-05 | 2.17E-05 | 7.36E-06 | 1.64E-05 | 2.81E-05 | 2.08E-05 | 1.22E-05 | 7.75E-06 | 2.05E-05 | 1.02E-05 | 1.16E-05 | 1.22E-05 |
| 3,4,5-Trimethoxycinnamic acid | 1.69E-05 | 2.73E-05 | 2.64E-05 | 3.88E-05 | 4.05E-05 | 4.44E-05 | 2.12E-05 | 1.47E-05 | 2.87E-05 | 2.39E-05 | 2.30E-05 | 2.62E-05 | 1.84E-05 | 2.56E-05 | 2.12E-05 | 1.55E-05 | 2.69E-05 | 2.20E-05 |
| Methyl-beta-galactopyranoside | 1.87E-05 | 1.55E-05 | 1.63E-05 | 3.04E-05 | 2.90E-05 | 2.97E-05 | 3.08E-05 | 2.32E-05 | 3.13E-05 | 3.86E-05 | 1.78E-05 | 3.58E-05 | 2.86E-05 | 2.29E-05 | 2.66E-05 | 3.24E-05 | 2.77E-05 | 3.16E-05 |
| D-Tryptophan | 2.39E-05 | 2.57E-05 | 1.83E-05 | 2.05E-05 | 1.30E-05 | 1.19E-05 | 2.36E-05 | 1.87E-05 | 3.75E-05 | 2.20E-05 | 2.85E-05 | 1.61E-05 | 2.50E-05 | 1.77E-05 | 3.00E-05 | 2.16E-05 | 2.89E-05 | 4.20E-05 |
| beta-Glucogallin | 3.73E-05 | 5.11E-05 | 3.54E-05 | 2.60E-05 | 2.45E-05 | 2.76E-05 | 2.37E-05 | 3.15E-05 | 3.11E-05 | 3.20E-05 | 3.09E-05 | 3.91E-05 | 1.94E-05 | 2.60E-05 | 2.89E-05 | 4.55E-05 | 3.40E-05 | 5.88E-05 |
| L-Tyrosine, N-[(2E)-3-(4-hydroxyphenyl)-1-oxo-2-propen-1-yl]- | 1.12E-05 | 1.43E-05 | 4.08E-05 | 4.58E-05 | 3.98E-05 | 6.67E-05 | 3.71E-05 | 9.69E-15 | 2.93E-05 | 5.78E-05 | 2.92E-05 | 5.83E-05 | 3.05E-05 | 4.66E-05 | 3.63E-05 | 7.38E-05 | 1.29E-05 | 1.30E-14 |
| m-Xylene | 3.26E-05 | 1.02E-14 | 1.19E-14 | 1.44E-14 | 1.32E-14 | 1.39E-14 | 1.17E-14 | 9.69E-15 | 1.06E-14 | 2.92E-05 | 2.00E-05 | 1.33E-14 | 1.07E-14 | 1.00E-14 | 9.43E-15 | 1.30E-14 | 1.26E-14 | 1.30E-14 |
| Peonidin-3-O-alpha-arabinopyranoside | 6.83E-05 | 1.71E-05 | 6.11E-06 | 8.20E-05 | 1.32E-14 | 5.34E-05 | 1.17E-14 | 2.76E-05 | 4.87E-05 | 3.11E-05 | 4.19E-05 | 2.43E-05 | 2.42E-05 | 5.55E-05 | 1.81E-05 | 2.90E-05 | 3.00E-05 | 2.37E-05 |
| Epigallocatechin gallate | 7.21E-05 | 7.11E-05 | 3.16E-05 | 1.41E-05 | 1.22E-05 | 1.05E-05 | 3.96E-05 | 3.75E-05 | 2.41E-05 | 2.42E-05 | 2.94E-05 | 1.58E-05 | 5.80E-05 | 2.02E-05 | 5.41E-05 | 3.43E-05 | 3.24E-05 | 6.30E-05 |
| N5-(4-Methoxybenzyl)glutamine | 8.45E-06 | 1.13E-05 | 2.06E-05 | 9.93E-06 | 3.49E-06 | 1.73E-05 | 1.27E-06 | 9.69E-15 | 1.16E-05 | 4.10E-06 | 1.99E-04 | 1.06E-05 | 5.86E-06 | 1.00E-14 | 9.43E-15 | 3.08E-05 | 1.16E-05 | 1.47E-05 |
| (3R,5R)-4-[(E)-3-(3,4-dihydroxyphenyl)prop-2-enoyl]oxy-1,3,5-trihydroxycyclohexane-1-carboxylic acid | 2.65E-05 | 3.29E-05 | 3.14E-05 | 6.78E-05 | 5.32E-05 | 6.54E-05 | 3.59E-05 | 1.76E-05 | 3.77E-05 | 3.02E-05 | 2.52E-05 | 2.80E-05 | 3.16E-05 | 1.50E-05 | 2.55E-05 | 2.39E-05 | 1.78E-05 | 2.17E-05 |
| Alternariol | 1.82E-05 | 2.12E-05 | 2.30E-05 | 2.26E-05 | 2.54E-05 | 2.86E-05 | 1.98E-05 | 1.58E-05 | 1.40E-05 | 1.58E-05 | 3.46E-05 | 3.00E-05 | 1.30E-05 | 9.07E-06 | 1.11E-05 | 1.20E-05 | 1.26E-14 | 2.08E-05 |
| 6-Hydroxykaempferol 4'-methyl ether 7-glucoside | 2.30E-05 | 1.36E-05 | 1.29E-05 | 2.85E-05 | 3.70E-05 | 3.70E-05 | 1.35E-05 | 3.84E-05 | 4.83E-05 | 1.52E-05 | 1.26E-05 | 3.08E-05 | 1.73E-05 | 3.31E-05 | 1.93E-05 | 1.61E-05 | 1.57E-05 | 1.99E-05 |
| 2-Cyclohexen-1-one, 4-hydroxy-4-(3-hydroxybutyl)-3,5,5-trimethyl- | 1.31E-05 | 2.10E-05 | 2.29E-05 | 3.09E-05 | 5.44E-05 | 5.09E-05 | 3.78E-05 | 2.10E-05 | 1.76E-04 | 2.48E-05 | 2.78E-05 | 1.98E-05 | 1.39E-05 | 1.92E-05 | 1.28E-05 | 2.68E-05 | 1.45E-05 | 2.07E-05 |
| Phloretin | 2.43E-06 | 4.29E-06 | 7.57E-06 | 5.43E-06 | 1.19E-05 | 5.62E-06 | 2.73E-05 | 5.43E-05 | 3.12E-05 | 1.42E-05 | 1.31E-14 | 2.58E-05 | 2.46E-05 | 2.01E-05 | 2.52E-05 | 4.35E-05 | 4.83E-05 | 2.11E-04 |
| Luteolin 5-methyl ether | 1.69E-05 | 2.15E-05 | 3.37E-05 | 5.30E-05 | 3.18E-05 | 5.00E-05 | 3.83E-05 | 4.87E-05 | 3.58E-05 | 7.89E-05 | 6.63E-05 | 5.71E-05 | 4.17E-05 | 4.98E-05 | 3.44E-05 | 1.14E-04 | 8.06E-05 | 1.76E-05 |
| 5-hydroxy-7-(hydroxymethyl)-2-methyl-2-(5-oxooxolan-2-yl)-3H-chromen-4-one | 2.09E-05 | 1.56E-05 | 2.45E-05 | 1.97E-05 | 3.25E-05 | 3.29E-05 | 9.69E-05 | 3.67E-05 | 4.47E-05 | 6.18E-05 | 3.58E-05 | 1.89E-05 | 2.31E-05 | 1.24E-05 | 1.76E-05 | 2.59E-05 | 8.46E-06 | 1.63E-05 |
| 6,8-dihydroxy-7-methoxy-3-methyl-3,4-dihydroisochromen-1-one | 2.36E-05 | 2.09E-05 | 1.53E-05 | 5.10E-05 | 3.95E-05 | 3.92E-05 | 6.86E-05 | 2.90E-05 | 3.12E-05 | 3.16E-05 | 2.86E-05 | 3.71E-05 | 3.37E-05 | 2.25E-05 | 3.72E-05 | 3.36E-05 | 2.74E-05 | 1.88E-05 |
| 1-O-Caffeoylglucose | 3.25E-05 | 2.05E-05 | 2.91E-05 | 2.82E-05 | 3.44E-05 | 3.04E-05 | 3.15E-05 | 2.86E-05 | 5.13E-05 | 2.45E-05 | 3.08E-05 | 2.74E-05 | 3.11E-05 | 4.48E-05 | 3.56E-05 | 3.49E-05 | 7.23E-05 | 3.98E-05 |
| 3-Phenoxyphenol | 8.81E-15 | 2.35E-05 | 3.66E-05 | 1.44E-14 | 3.35E-05 | 2.67E-05 | 1.17E-14 | 2.53E-05 | 3.54E-05 | 2.18E-05 | 3.62E-05 | 1.33E-14 | 1.35E-05 | 3.73E-05 | 2.58E-05 | 7.35E-05 | 1.81E-05 | 4.12E-05 |
| NCGC00380817-01 | 2.04E-05 | 3.70E-05 | 2.74E-05 | 3.24E-05 | 3.97E-05 | 4.73E-05 | 5.31E-05 | 3.83E-05 | 4.01E-05 | 4.36E-05 | 3.27E-05 | 4.00E-05 | 2.26E-05 | 2.01E-05 | 3.83E-05 | 3.21E-05 | 2.72E-05 | 5.02E-05 |
| NCGC00170013-02 | 6.00E-05 | 7.11E-05 | 2.65E-05 | 1.94E-05 | 1.80E-05 | 1.05E-05 | 9.88E-06 | 6.43E-05 | 7.55E-06 | 3.53E-05 | 4.77E-05 | 1.16E-05 | 1.85E-05 | 5.85E-06 | 3.34E-05 | 1.38E-05 | 4.00E-05 | 4.84E-05 |
| QUINALIZARIN | 2.45E-05 | 3.19E-05 | 2.26E-05 | 4.22E-05 | 3.53E-05 | 2.79E-05 | 4.63E-05 | 3.86E-05 | 2.51E-05 | 3.39E-05 | 2.42E-05 | 4.56E-05 | 6.16E-05 | 3.80E-05 | 3.29E-05 | 3.10E-05 | 3.98E-05 | 3.62E-05 |
| 4'-Hydroxy-5,7,2'-trimethoxyflavanone 4'-rhamnosyl-(1->6)-glucoside | 1.13E-05 | 1.51E-05 | 4.16E-05 | 1.93E-05 | 2.02E-05 | 2.17E-05 | 6.58E-05 | 1.83E-05 | 2.78E-05 | 2.91E-05 | 3.42E-05 | 3.65E-05 | 2.31E-05 | 3.69E-05 | 3.82E-05 | 3.04E-05 | 2.84E-05 | 1.71E-05 |
| Uvaol | 1.91E-05 | 1.13E-05 | 1.92E-05 | 8.93E-06 | 3.28E-05 | 1.71E-05 | 3.28E-05 | 3.34E-05 | 1.63E-05 | 7.12E-05 | 2.30E-05 | 2.78E-05 | 5.75E-05 | 2.03E-05 | 3.95E-05 | 1.98E-05 | 3.85E-05 | 2.23E-05 |
| Apigenin | 6.01E-05 | 2.67E-05 | 3.10E-05 | 4.29E-05 | 6.34E-05 | 8.20E-05 | 2.50E-05 | 2.34E-05 | 2.88E-05 | 4.64E-05 | 4.03E-05 | 4.05E-05 | 3.32E-05 | 1.74E-05 | 2.43E-05 | 3.00E-05 | 1.26E-14 | 3.70E-05 |
| Pseudohypericin | 3.48E-05 | 3.37E-05 | 1.57E-04 | 2.12E-05 | 3.72E-05 | 1.46E-05 | 1.86E-05 | 6.95E-05 | 3.33E-05 | 4.61E-05 | 4.91E-05 | 4.45E-05 | 3.21E-05 | 2.75E-05 | 3.46E-05 | 1.63E-05 | 6.78E-05 | 3.60E-05 |
| beta-Sitosterol | 5.01E-05 | 2.56E-05 | 4.25E-05 | 5.62E-05 | 3.48E-05 | 4.70E-05 | 3.50E-05 | 5.31E-05 | 2.79E-05 | 5.99E-05 | 1.01E-04 | 1.80E-05 | 6.15E-05 | 5.29E-05 | 3.59E-05 | 2.06E-05 | 4.22E-05 | 4.93E-05 |
| U-44069 | 2.08E-05 | 2.95E-05 | 1.41E-05 | 3.05E-05 | 2.20E-05 | 1.97E-05 | 9.39E-05 | 8.57E-06 | 8.09E-05 | 1.21E-04 | 7.99E-05 | 6.74E-05 | 2.44E-05 | 3.60E-05 | 3.66E-05 | 2.17E-05 | 2.95E-05 | 8.93E-06 |
| 3-Carbethoxypsoralen | 1.08E-05 | 2.53E-05 | 3.84E-05 | 5.54E-05 | 8.41E-05 | 1.05E-04 | 5.01E-05 | 2.70E-05 | 5.32E-05 | 2.07E-05 | 2.33E-05 | 3.37E-05 | 2.09E-05 | 1.81E-05 | 1.70E-05 | 2.37E-05 | 1.46E-05 | 1.74E-05 |
| 5,7-dihydroxy-2-(4-hydroxyphenyl)-6-[3,4,5-trihydroxy-6-(hydroxymethyl)oxan-2-yl]-2,3-dihydrochromen-4-one | 6.49E-05 | 8.18E-05 | 1.00E-04 | 1.30E-04 | 7.15E-05 | 1.44E-04 | 8.64E-05 | 3.68E-05 | 1.52E-04 | 1.02E-04 | 3.00E-05 | 1.18E-04 | 8.04E-05 | 1.12E-04 | 3.06E-05 | 1.14E-04 | 7.90E-05 | 8.08E-05 |
| delta-Tridecalactone | 2.79E-05 | 2.86E-05 | 4.38E-05 | 5.62E-05 | 8.00E-05 | 8.64E-05 | 6.55E-05 | 4.85E-05 | 5.66E-05 | 3.10E-05 | 5.26E-05 | 5.24E-05 | 2.62E-05 | 2.60E-05 | 3.33E-05 | 4.58E-05 | 2.65E-05 | 2.76E-05 |
| BRAZILEIN | 2.94E-05 | 4.36E-05 | 5.59E-05 | 5.57E-05 | 7.69E-05 | 5.31E-05 | 4.90E-05 | 4.78E-05 | 6.07E-05 | 4.99E-05 | 5.28E-05 | 7.11E-05 | 2.80E-05 | 2.88E-05 | 3.75E-05 | 4.93E-05 | 4.67E-05 | 5.67E-05 |
| Peltatoside | 2.89E-05 | 4.50E-05 | 3.35E-05 | 7.90E-05 | 3.26E-05 | 2.34E-05 | 6.21E-05 | 4.66E-05 | 4.02E-05 | 4.43E-05 | 3.31E-05 | 8.66E-05 | 3.96E-05 | 3.50E-05 | 3.84E-05 | 2.24E-05 | 8.09E-05 | 3.72E-05 |
| 5-cis Carbaprostacyclin | 2.59E-05 | 2.58E-05 | 4.01E-05 | 5.02E-05 | 3.52E-05 | 4.45E-05 | 3.24E-05 | 4.73E-05 | 2.72E-05 | 6.22E-05 | 6.18E-05 | 1.75E-05 | 7.72E-05 | 3.04E-05 | 3.28E-05 | 1.85E-05 | 4.85E-05 | 3.56E-05 |
| Diphenylamine | 1.87E-04 | 8.62E-05 | 4.42E-05 | 9.13E-05 | 1.74E-04 | 1.39E-14 | 4.00E-05 | 1.81E-04 | 7.95E-05 | 7.08E-05 | 9.46E-05 | 1.33E-14 | 3.58E-05 | 5.61E-05 | 9.43E-15 | 1.78E-04 | 1.26E-14 | 6.00E-05 |
| EMODIC ACID | 1.48E-05 | 2.06E-05 | 4.31E-05 | 6.74E-05 | 8.23E-05 | 1.00E-04 | 6.99E-05 | 1.55E-05 | 4.79E-05 | 2.78E-05 | 2.78E-05 | 4.81E-05 | 3.50E-05 | 4.42E-05 | 2.18E-05 | 4.33E-05 | 2.76E-05 | 4.29E-05 |
| 2,4,6-trihydroxy-2-[(4-hydroxyphenyl)methyl]-2,3-dihydro-1-benzofuran-3-one | 1.06E-05 | 9.58E-06 | 1.23E-05 | 8.87E-06 | 1.03E-05 | 1.40E-05 | 9.54E-06 | 9.71E-06 | 1.78E-05 | 4.35E-06 | 1.52E-04 | 7.75E-06 | 6.99E-06 | 7.57E-06 | 7.52E-06 | 2.61E-04 | 7.85E-06 | 3.52E-04 |
| Shanciol | 2.74E-05 | 3.40E-05 | 5.98E-05 | 7.79E-05 | 6.47E-05 | 6.48E-05 | 5.98E-05 | 4.94E-05 | 5.99E-05 | 5.36E-05 | 4.23E-05 | 6.67E-05 | 6.17E-05 | 6.26E-05 | 4.94E-05 | 6.57E-05 | 3.84E-05 | 4.53E-05 |
| Glucocaffeic acid | 1.97E-05 | 5.93E-05 | 4.98E-05 | 3.93E-05 | 4.95E-05 | 1.39E-14 | 5.03E-05 | 5.63E-05 | 4.62E-05 | 6.64E-05 | 2.40E-05 | 2.65E-05 | 4.09E-05 | 6.57E-05 | 6.01E-05 | 3.85E-05 | 6.70E-05 | 6.21E-05 |
| Ascorbic acid | 8.81E-15 | 2.28E-05 | 1.19E-14 | 1.44E-14 | 1.32E-14 | 1.39E-14 | 1.17E-14 | 9.69E-15 | 4.09E-05 | 3.73E-05 | 1.31E-14 | 1.33E-14 | 5.38E-05 | 6.55E-05 | 3.66E-05 | 1.30E-14 | 1.26E-14 | 1.30E-14 |
| 2-(6-hydroxy-6-methylheptyl)-2H-furan-5-one | 4.10E-05 | 5.24E-05 | 4.71E-05 | 6.14E-05 | 6.19E-05 | 6.14E-05 | 4.92E-05 | 4.20E-05 | 4.17E-05 | 4.59E-05 | 5.85E-05 | 5.65E-05 | 4.34E-05 | 4.46E-05 | 3.71E-05 | 5.77E-05 | 6.66E-05 | 5.33E-05 |
| (S)-p-Mentha-1,8-dien-7-ol | 3.70E-05 | 3.45E-05 | 3.78E-05 | 4.69E-05 | 2.74E-05 | 2.32E-05 | 3.24E-05 | 3.09E-05 | 2.99E-05 | 5.37E-05 | 2.11E-05 | 2.46E-05 | 7.52E-05 | 3.21E-05 | 4.79E-05 | 1.87E-05 | 5.66E-05 | 2.66E-05 |
| alpha-curcumene | 4.06E-05 | 4.61E-05 | 5.36E-05 | 6.82E-05 | 6.82E-05 | 6.15E-05 | 5.22E-05 | 4.39E-05 | 4.85E-05 | 5.63E-05 | 6.63E-05 | 6.34E-05 | 4.71E-05 | 5.52E-05 | 4.15E-05 | 5.84E-05 | 5.92E-05 | 5.94E-05 |
| (E)-3-[4-methoxy-2-[(2S,3R,4S,5S,6R)-3,4,5-trihydroxy-6-(hydroxymethyl)oxan-2-yl]oxyphenyl]prop-2-enoic acid | 1.05E-05 | 4.20E-05 | 3.35E-05 | 4.35E-05 | 2.45E-05 | 2.10E-05 | 3.32E-05 | 1.09E-04 | 2.21E-05 | 6.18E-05 | 2.62E-05 | 3.29E-05 | 4.19E-05 | 2.85E-05 | 5.85E-05 | 4.92E-05 | 1.03E-04 | 1.44E-04 |
| 5,7-Dihydroxyflavone | 1.63E-04 | 1.19E-04 | 1.55E-04 | 2.51E-04 | 1.44E-04 | 2.09E-04 | 1.12E-05 | 9.60E-06 | 1.95E-05 | 7.74E-06 | 9.00E-06 | 2.27E-05 | 3.58E-06 | 6.41E-06 | 4.80E-06 | 5.37E-06 | 5.05E-06 | 6.54E-06 |
| Citropten | 4.04E-05 | 5.74E-05 | 4.49E-05 | 6.13E-05 | 6.81E-05 | 6.88E-05 | 4.55E-05 | 3.85E-05 | 6.22E-05 | 4.48E-05 | 2.98E-05 | 3.61E-05 | 3.27E-05 | 4.91E-05 | 4.58E-05 | 2.83E-05 | 4.50E-05 | 3.48E-05 |
| Aesculetin | 6.00E-05 | 7.90E-05 | 6.47E-05 | 6.36E-05 | 3.59E-05 | 3.50E-05 | 5.98E-05 | 6.33E-05 | 4.32E-05 | 5.65E-05 | 4.85E-05 | 4.97E-05 | 3.67E-05 | 2.99E-05 | 4.04E-05 | 3.66E-05 | 1.26E-14 | 5.62E-05 |
| Prunetin | 1.15E-04 | 7.52E-05 | 1.87E-04 | 1.52E-04 | 6.98E-05 | 1.28E-04 | 1.15E-05 | 2.55E-06 | 1.12E-05 | 3.28E-06 | 4.88E-06 | 1.09E-05 | 2.47E-06 | 3.59E-06 | 2.24E-06 | 4.48E-06 | 5.51E-06 | 4.65E-06 |
| Thiourocanic acid | 8.81E-15 | 1.58E-05 | 2.04E-05 | 8.93E-05 | 1.24E-04 | 9.53E-05 | 2.92E-05 | 1.21E-05 | 2.01E-05 | 1.58E-05 | 1.50E-05 | 1.18E-05 | 1.58E-05 | 1.89E-05 | 1.64E-05 | 3.05E-05 | 2.78E-05 | 1.77E-05 |
| 3-Methoxy-8,9-methylenedioxy-3,4-dihydrophenanthridine | 4.46E-05 | 5.88E-05 | 7.03E-05 | 7.71E-05 | 6.53E-05 | 5.63E-05 | 5.80E-05 | 4.67E-05 | 4.99E-05 | 5.77E-05 | 7.07E-05 | 7.60E-05 | 4.93E-05 | 5.73E-05 | 4.78E-05 | 6.79E-05 | 6.38E-05 | 6.58E-05 |
| 4-(4-Hydroxy-2,6,6-trimethyl-1-cyclohexen-1-yl)-2-butanyl beta-D-glucopyranoside | 1.83E-05 | 2.98E-05 | 5.54E-05 | 6.15E-05 | 9.93E-05 | 1.31E-04 | 9.32E-05 | 3.50E-05 | 9.57E-05 | 3.26E-05 | 6.70E-05 | 6.15E-05 | 2.69E-05 | 3.56E-05 | 2.09E-05 | 6.41E-05 | 2.73E-05 | 3.33E-05 |
| fuzhuanin A | 7.42E-05 | 8.26E-05 | 7.88E-05 | 4.22E-05 | 5.82E-05 | 4.18E-05 | 5.55E-05 | 7.01E-05 | 5.90E-05 | 3.32E-05 | 4.76E-05 | 5.54E-05 | 6.56E-05 | 9.07E-05 | 7.79E-05 | 8.03E-05 | 6.40E-05 | 4.91E-05 |
| Sinapic acid | 3.17E-05 | 3.49E-05 | 2.94E-05 | 3.88E-05 | 2.50E-05 | 6.35E-05 | 1.02E-04 | 1.28E-04 | 6.26E-05 | 5.77E-05 | 2.58E-05 | 6.20E-05 | 5.44E-05 | 3.72E-05 | 6.06E-05 | 4.84E-05 | 7.80E-05 | 1.21E-04 |
| Octopine | 1.28E-04 | 1.19E-04 | 9.87E-05 | 1.19E-04 | 1.50E-04 | 1.19E-04 | 6.57E-05 | 5.31E-05 | 4.74E-05 | 4.81E-05 | 3.58E-05 | 3.20E-05 | 3.08E-05 | 2.11E-05 | 3.87E-05 | 6.32E-05 | 4.84E-05 | 5.00E-05 |
| 2,6,7-Trihydroxy-9-methylxanthen-3-one | 8.88E-05 | 1.47E-04 | 2.33E-04 | 1.04E-04 | 6.43E-05 | 8.03E-05 | 6.01E-05 | 1.23E-04 | 2.69E-05 | 5.26E-05 | 9.73E-05 | 6.40E-05 | 2.53E-05 | 5.75E-05 | 3.21E-05 | 6.48E-05 | 7.76E-05 | 7.26E-05 |
| beta-Zearalenol | 1.89E-05 | 3.65E-05 | 3.03E-05 | 6.94E-05 | 3.23E-05 | 1.77E-05 | 4.40E-05 | 3.16E-05 | 2.14E-05 | 3.12E-05 | 1.05E-04 | 6.18E-05 | 2.32E-05 | 8.64E-05 | 3.22E-05 | 5.07E-05 | 6.07E-05 | 3.01E-05 |
| (S)-Pinocembrin | 1.79E-04 | 1.26E-04 | 1.66E-04 | 2.98E-04 | 1.49E-04 | 1.95E-04 | 2.00E-05 | 1.49E-05 | 1.03E-05 | 1.18E-05 | 3.34E-05 | 3.42E-05 | 1.60E-05 | 1.16E-05 | 1.46E-05 | 1.61E-05 | 9.96E-06 | 1.71E-05 |
| alpha-D-Glucopyranoside, beta-L-fructofuranosyl 6-O-[(2E)-3-(4-hydroxyphenyl)-1-oxo-2-propen-1-yl]- | 2.22E-05 | 3.75E-05 | 9.44E-05 | 8.76E-05 | 1.20E-04 | 1.06E-04 | 6.00E-05 | 5.05E-05 | 6.54E-05 | 1.16E-04 | 4.82E-05 | 7.75E-05 | 1.02E-04 | 1.08E-04 | 9.59E-05 | 1.35E-04 | 2.34E-05 | 7.13E-05 |
| 2,2,6,7-Tetramethylbicyclo[4.3.0]nona-1(9),4-diene-7,8-diol | 1.55E-05 | 1.59E-05 | 6.17E-05 | 7.66E-05 | 1.04E-04 | 8.67E-05 | 1.19E-04 | 3.02E-05 | 1.11E-04 | 4.78E-05 | 7.06E-05 | 6.35E-05 | 2.97E-05 | 4.09E-05 | 2.91E-05 | 9.62E-05 | 2.23E-05 | 2.84E-05 |
| 5,7-DIHYDROXY-4-METHYLCOUMARIN | 6.59E-05 | 7.28E-05 | 9.22E-05 | 8.13E-05 | 1.12E-04 | 1.34E-04 | 5.27E-05 | 6.03E-05 | 4.33E-05 | 5.96E-05 | 7.91E-05 | 7.04E-05 | 3.99E-05 | 4.95E-05 | 5.70E-05 | 6.01E-05 | 3.41E-05 | 7.19E-05 |
| 3-methylquercetin | 1.21E-04 | 6.81E-05 | 5.84E-05 | 7.09E-05 | 8.13E-05 | 1.07E-04 | 5.70E-05 | 3.24E-05 | 3.30E-05 | 1.25E-04 | 1.43E-04 | 3.74E-05 | 1.09E-04 | 3.97E-05 | 9.43E-15 | 3.99E-05 | 7.69E-05 | 4.09E-05 |
| (5E,11E)-7,8,14,16-Tetrahydroxy-3-methyl-3,4,7,8,9,10-hexahydro-1H-2-benzoxacyclotetradecin-1-one | 9.20E-05 | 9.49E-05 | 7.47E-05 | 6.74E-05 | 4.06E-05 | 2.91E-05 | 3.25E-05 | 7.40E-05 | 2.68E-05 | 6.83E-05 | 5.16E-05 | 7.09E-05 | 5.82E-05 | 5.87E-05 | 9.73E-05 | 6.24E-05 | 1.15E-04 | 8.81E-05 |
| Galactose 1-phosphate | 4.30E-05 | 5.75E-05 | 3.93E-05 | 6.14E-05 | 5.28E-05 | 4.35E-05 | 8.91E-05 | 9.28E-05 | 6.08E-05 | 9.36E-05 | 9.80E-05 | 5.30E-05 | 9.68E-05 | 8.23E-05 | 1.10E-04 | 5.68E-05 | 1.45E-04 | 9.79E-05 |
| [6-[2-(3,4-dihydroxyphenyl)-8-hydroxy-4-oxochromen-7-yl]oxy-3,4,5-trihydroxyoxan-2-yl]methyl (E)-3-(4-hydroxyphenyl)prop-2-enoate | 4.25E-05 | 4.36E-05 | 6.24E-05 | 1.59E-04 | 8.53E-05 | 1.30E-04 | 1.04E-04 | 5.37E-05 | 7.30E-05 | 8.71E-05 | 4.61E-05 | 1.09E-04 | 5.43E-05 | 6.23E-05 | 5.92E-05 | 7.48E-05 | 5.93E-05 | 6.45E-05 |
| Lapachol | 3.65E-05 | 1.11E-04 | 5.11E-05 | 9.55E-05 | 4.70E-05 | 1.77E-05 | 8.26E-05 | 7.28E-05 | 5.90E-05 | 6.23E-05 | 1.41E-04 | 9.36E-05 | 2.12E-05 | 8.83E-05 | 2.77E-05 | 7.06E-05 | 1.14E-04 | 4.54E-05 |
| Nanaomycin | 6.91E-05 | 7.01E-05 | 7.05E-05 | 6.07E-05 | 4.08E-05 | 6.03E-05 | 7.16E-05 | 9.48E-05 | 8.12E-05 | 8.83E-05 | 7.50E-05 | 9.85E-05 | 9.64E-05 | 6.78E-05 | 7.77E-05 | 4.49E-05 | 1.18E-04 | 1.27E-04 |
| Methyl (R)-9-hydroxy-10-undecene-5,7-diynoate glucoside | 2.70E-05 | 3.52E-05 | 5.68E-05 | 9.13E-05 | 1.63E-04 | 1.20E-04 | 1.28E-04 | 6.20E-05 | 1.20E-04 | 4.91E-05 | 1.31E-14 | 8.30E-05 | 6.42E-05 | 7.03E-05 | 4.46E-05 | 8.49E-05 | 6.66E-05 | 3.94E-05 |
| N-ACETYLPROLINE | 5.07E-05 | 5.05E-05 | 6.32E-05 | 5.90E-05 | 9.34E-05 | 9.32E-05 | 1.10E-04 | 9.73E-05 | 7.26E-05 | 8.11E-05 | 6.19E-05 | 9.20E-05 | 8.04E-05 | 7.32E-05 | 8.49E-05 | 4.21E-05 | 7.35E-05 | 9.91E-05 |
| 5-Hydroxymethyl-2-furancarboxaldehyde | 2.41E-05 | 3.05E-05 | 3.69E-05 | 5.57E-05 | 8.69E-05 | 7.02E-05 | 1.10E-04 | 5.73E-05 | 1.26E-04 | 6.28E-05 | 5.18E-05 | 8.71E-05 | 4.20E-05 | 6.55E-05 | 4.90E-05 | 7.84E-05 | 6.19E-05 | 4.04E-05 |
| p-Mentha-1,3,8-triene | 8.81E-15 | 3.70E-05 | 6.34E-05 | 1.13E-04 | 1.25E-04 | 1.39E-14 | 9.50E-05 | 3.28E-05 | 7.56E-05 | 3.18E-05 | 8.91E-05 | 1.33E-14 | 3.42E-05 | 4.90E-05 | 6.49E-05 | 8.04E-05 | 6.73E-05 | 1.30E-14 |
| 2,4-Diisopropyl-5-methylphenol | 7.25E-05 | 9.64E-05 | 1.05E-04 | 9.64E-05 | 9.50E-05 | 1.46E-04 | 5.77E-05 | 7.18E-05 | 4.58E-05 | 6.85E-05 | 8.60E-05 | 7.56E-05 | 5.93E-05 | 5.36E-05 | 5.38E-05 | 5.66E-05 | 3.58E-05 | 6.45E-05 |
| 1-O-Sinapoyl-尾-D-glucose | 7.89E-05 | 9.27E-05 | 6.16E-05 | 8.67E-05 | 1.03E-04 | 7.08E-05 | 1.08E-04 | 1.02E-04 | 6.44E-05 | 7.84E-05 | 7.41E-05 | 7.73E-05 | 8.52E-05 | 6.59E-05 | 8.86E-05 | 4.49E-05 | 1.07E-04 | 9.06E-05 |
| Methyl caffeate | 6.21E-05 | 8.98E-05 | 7.81E-05 | 7.21E-05 | 6.03E-05 | 8.42E-05 | 8.89E-05 | 6.09E-05 | 4.83E-05 | 6.89E-05 | 6.22E-05 | 5.95E-05 | 6.98E-05 | 4.74E-05 | 5.80E-05 | 4.07E-05 | 7.56E-05 | 5.91E-05 |
| Genistein 4'-O-glucoside | 5.16E-04 | 4.33E-04 | 1.09E-04 | 1.85E-05 | 1.59E-05 | 8.41E-06 | 8.20E-05 | 9.30E-05 | 5.68E-05 | 4.03E-06 | 6.26E-06 | 4.79E-06 | 2.02E-04 | 9.48E-05 | 9.99E-05 | 1.30E-14 | 1.20E-05 | 1.41E-05 |
| 4-Hydroxybenzoic acid | 1.27E-04 | 8.92E-05 | 9.23E-05 | 1.01E-04 | 4.81E-05 | 7.50E-05 | 5.50E-05 | 3.42E-05 | 3.20E-05 | 4.48E-05 | 5.77E-05 | 3.57E-05 | 6.69E-05 | 4.29E-05 | 4.47E-05 | 4.49E-05 | 5.21E-05 | 4.08E-05 |
| Isoferulic acid | 7.69E-05 | 1.07E-04 | 9.32E-05 | 1.22E-04 | 6.22E-05 | 6.28E-05 | 4.81E-05 | 6.72E-05 | 3.71E-05 | 8.31E-05 | 1.29E-04 | 5.16E-05 | 9.49E-05 | 6.28E-05 | 7.42E-05 | 4.33E-05 | 7.34E-05 | 2.32E-05 |
| Scytalone | 2.88E-05 | 5.77E-05 | 6.75E-05 | 1.02E-04 | 5.63E-05 | 7.74E-05 | 9.97E-05 | 5.82E-05 | 6.26E-05 | 8.01E-05 | 6.13E-05 | 6.51E-05 | 4.88E-05 | 4.76E-05 | 4.61E-05 | 9.14E-05 | 4.18E-05 | 8.31E-05 |
| CAY10616 | 3.09E-05 | 4.74E-05 | 6.95E-05 | 7.23E-05 | 5.67E-05 | 1.72E-05 | 5.70E-05 | 7.29E-05 | 5.54E-05 | 6.98E-05 | 1.13E-04 | 1.11E-04 | 4.49E-05 | 1.22E-04 | 5.81E-05 | 8.40E-05 | 1.36E-04 | 7.20E-05 |
| Prephenate | 4.50E-05 | 1.05E-04 | 9.80E-05 | 8.58E-05 | 5.39E-05 | 5.13E-05 | 9.73E-05 | 2.03E-04 | 8.55E-05 | 7.57E-05 | 8.51E-05 | 7.84E-05 | 6.44E-05 | 5.58E-05 | 7.43E-05 | 1.05E-04 | 1.54E-04 | 2.52E-04 |
| Vanillylmandelic acid | 3.51E-05 | 7.21E-05 | 1.10E-04 | 1.97E-04 | 3.21E-04 | 3.02E-04 | 1.21E-04 | 7.04E-05 | 1.89E-04 | 7.28E-05 | 7.46E-05 | 1.21E-04 | 4.31E-05 | 4.84E-05 | 5.05E-05 | 8.05E-05 | 4.38E-05 | 4.15E-05 |
| (3R)-4,4-Dimethyl-2-oxotetrahydro-3-furanyl beta-D-glucopyranoside | 1.78E-05 | 2.15E-05 | 4.98E-05 | 3.97E-05 | 3.34E-05 | 2.37E-05 | 1.24E-04 | 6.27E-05 | 8.91E-05 | 9.40E-05 | 7.68E-05 | 9.87E-05 | 9.92E-05 | 5.60E-05 | 8.26E-05 | 9.65E-05 | 6.37E-05 | 6.92E-05 |
| Epiandrosterone | 4.42E-05 | 5.70E-05 | 2.79E-05 | 5.87E-05 | 3.42E-05 | 4.57E-05 | 1.53E-04 | 1.38E-05 | 9.71E-05 | 1.95E-04 | 1.39E-04 | 1.01E-04 | 4.10E-05 | 4.78E-05 | 5.98E-05 | 3.03E-05 | 3.98E-05 | 2.12E-05 |
| 7,2'-Dihydroxy-3',4'-dimethoxyisoflavone 7-O-glucoside | 8.72E-05 | 8.00E-05 | 9.08E-05 | 3.69E-05 | 7.69E-05 | 5.96E-05 | 6.31E-05 | 1.05E-04 | 1.02E-04 | 1.19E-04 | 1.04E-04 | 1.89E-04 | 5.31E-05 | 5.81E-05 | 8.79E-05 | 1.10E-04 | 1.22E-04 | 1.42E-04 |
| Piperitenone | 4.15E-05 | 5.59E-05 | 1.10E-04 | 1.59E-04 | 1.51E-04 | 1.94E-04 | 9.30E-05 | 4.94E-05 | 1.08E-04 | 6.17E-05 | 1.04E-04 | 1.06E-04 | 4.08E-05 | 6.48E-05 | 4.83E-05 | 1.15E-04 | 4.38E-05 | 5.31E-05 |
| Diethyl phthalic acid | 8.64E-05 | 9.98E-05 | 1.16E-04 | 1.41E-04 | 1.30E-04 | 1.48E-04 | 1.13E-04 | 9.71E-05 | 1.06E-04 | 9.83E-05 | 1.30E-04 | 1.40E-04 | 9.85E-05 | 9.43E-05 | 8.44E-05 | 1.16E-04 | 1.24E-04 | 1.17E-04 |
| Protocatechuic acid | 3.37E-05 | 8.51E-05 | 4.53E-05 | 7.20E-05 | 1.84E-05 | 5.85E-05 | 2.64E-05 | 4.45E-05 | 9.51E-06 | 2.05E-04 | 4.07E-05 | 2.88E-04 | 2.96E-04 | 1.12E-04 | 1.86E-04 | 7.71E-05 | 1.20E-04 | 1.60E-04 |
| 4-Coumaryl alcohol | 8.81E-15 | 9.30E-05 | 1.04E-04 | 8.73E-05 | 7.99E-05 | 1.34E-04 | 8.57E-05 | 1.51E-04 | 1.06E-14 | 1.22E-04 | 5.12E-05 | 5.53E-05 | 8.84E-05 | 1.13E-04 | 1.12E-04 | 1.30E-14 | 1.08E-04 | 1.34E-04 |
| Ser Cys Asn | 7.60E-05 | 1.35E-04 | 1.07E-04 | 1.07E-04 | 9.24E-05 | 9.48E-05 | 8.68E-05 | 1.30E-04 | 8.80E-05 | 1.47E-04 | 9.26E-05 | 9.73E-05 | 8.94E-05 | 9.72E-05 | 9.37E-05 | 6.57E-05 | 1.56E-04 | 9.75E-05 |
| Myricetin 3'-O-(6''-p-coumaroyl)glucoside | 8.70E-05 | 1.10E-04 | 7.31E-05 | 1.12E-04 | 5.98E-05 | 9.39E-05 | 1.35E-04 | 2.00E-04 | 1.61E-04 | 1.17E-04 | 7.22E-05 | 1.20E-04 | 1.29E-04 | 1.64E-04 | 1.73E-04 | 1.14E-04 | 7.90E-05 | 6.94E-05 |
| Genistein | 3.95E-05 | 7.49E-05 | 8.56E-05 | 8.53E-05 | 1.31E-04 | 1.40E-04 | 1.70E-04 | 1.26E-04 | 1.41E-04 | 1.74E-04 | 9.99E-05 | 1.85E-04 | 1.37E-04 | 1.44E-04 | 1.38E-04 | 1.73E-04 | 1.86E-04 | 1.87E-04 |
| (卤)8(9)-EET methyl ester | 9.37E-05 | 4.54E-05 | 2.56E-05 | 7.79E-05 | 7.53E-05 | 2.05E-04 | 1.61E-04 | 1.45E-05 | 1.21E-04 | 3.21E-04 | 1.03E-04 | 1.73E-04 | 1.42E-04 | 8.28E-05 | 1.57E-04 | 4.72E-05 | 5.73E-05 | 3.57E-05 |
| Eucommin A | 1.83E-05 | 2.56E-05 | 4.32E-05 | 5.76E-05 | 1.20E-04 | 7.40E-05 | 2.19E-04 | 7.51E-05 | 3.53E-04 | 5.98E-05 | 3.10E-05 | 1.68E-04 | 6.96E-05 | 1.36E-04 | 6.23E-05 | 1.32E-04 | 4.95E-05 | 5.43E-05 |
| (S)-5'-Deoxy-5'-(methylsulfinyl)adenosine | 5.47E-05 | 8.03E-05 | 6.73E-05 | 1.22E-04 | 6.56E-05 | 3.75E-05 | 7.40E-05 | 9.49E-05 | 3.66E-05 | 1.28E-04 | 2.51E-05 | 7.04E-05 | 1.07E-04 | 7.61E-05 | 9.86E-05 | 5.24E-05 | 1.08E-04 | 1.71E-04 |
| Methylgallate | 2.20E-04 | 1.19E-04 | 1.30E-04 | 3.88E-05 | 4.04E-05 | 3.21E-05 | 3.89E-05 | 5.31E-05 | 5.21E-05 | 1.24E-04 | 1.03E-04 | 7.71E-05 | 7.12E-05 | 5.14E-05 | 7.32E-05 | 8.69E-05 | 6.85E-05 | 2.46E-04 |
| 2-Butenoic acid, 2-methyl-, (3aR,4R,5R,9aS,9bR)-2,3,3a,4,5,7,9a,9b-octahydro-4-hydroxy-6,9-dimethyl-3-methylene-2,7-dioxoazuleno[4,5-b]furan-5-yl ester, (2Z)- | 8.33E-05 | 5.25E-05 | 1.48E-04 | 6.27E-05 | 5.70E-05 | 4.85E-05 | 7.40E-05 | 2.19E-04 | 2.57E-04 | 5.94E-05 | 3.53E-05 | 4.64E-05 | 6.19E-05 | 1.41E-04 | 1.64E-04 | 2.27E-04 | 1.04E-04 | 2.62E-04 |
| 8-Epiiridodial | 4.63E-05 | 4.37E-05 | 1.22E-04 | 1.14E-04 | 2.14E-04 | 2.07E-04 | 1.91E-04 | 9.21E-05 | 2.53E-04 | 1.19E-04 | 1.68E-04 | 1.66E-04 | 1.24E-04 | 1.19E-04 | 6.58E-05 | 1.24E-04 | 6.44E-05 | 7.95E-05 |
| D-Maltose | 1.07E-04 | 1.24E-04 | 1.69E-04 | 4.44E-04 | 1.34E-04 | 2.05E-04 | 1.51E-04 | 1.32E-04 | 1.11E-04 | 1.04E-04 | 1.92E-04 | 1.22E-04 | 1.59E-04 | 1.44E-04 | 9.02E-05 | 1.33E-04 | 1.75E-04 | 1.27E-04 |
| Benzotriazole-5-carboxylic acid | 7.12E-05 | 1.11E-04 | 1.14E-04 | 1.17E-04 | 8.69E-05 | 1.11E-04 | 1.28E-04 | 2.02E-04 | 1.23E-04 | 1.49E-04 | 1.19E-04 | 1.24E-04 | 7.96E-05 | 1.47E-04 | 8.17E-05 | 8.82E-05 | 2.01E-04 | 9.18E-05 |
| 2-Phospho-D-glyceric acid | 1.37E-04 | 1.39E-04 | 1.10E-04 | 2.63E-04 | 1.41E-04 | 1.47E-04 | 1.54E-04 | 2.02E-04 | 1.57E-04 | 1.58E-04 | 2.09E-04 | 1.51E-04 | 1.89E-04 | 2.19E-04 | 2.88E-04 | 1.87E-04 | 2.38E-04 | 2.46E-04 |
| Aspirin | 1.73E-04 | 1.24E-04 | 3.41E-04 | 1.04E-04 | 5.14E-05 | 6.84E-05 | 5.65E-05 | 1.24E-04 | 6.35E-05 | 1.92E-04 | 7.53E-05 | 9.59E-05 | 1.08E-04 | 2.22E-04 | 1.27E-04 | 7.48E-05 | 1.58E-04 | 1.15E-04 |
| 2,4,5,6-Phenanthrenetetrol | 1.33E-04 | 1.79E-04 | 1.28E-04 | 1.66E-04 | 1.57E-04 | 8.31E-05 | 2.11E-04 | 1.85E-04 | 1.91E-04 | 1.81E-04 | 1.59E-04 | 2.05E-04 | 1.38E-04 | 1.41E-04 | 1.52E-04 | 1.22E-04 | 1.87E-04 | 2.17E-04 |
| M-toluic Acid | 1.04E-04 | 1.24E-04 | 1.71E-04 | 2.26E-04 | 2.88E-04 | 2.85E-04 | 2.12E-04 | 7.81E-05 | 1.86E-04 | 9.31E-05 | 3.53E-04 | 1.38E-04 | 9.43E-05 | 9.23E-05 | 9.43E-15 | 1.59E-04 | 1.14E-04 | 1.15E-04 |
| 4-Methoxybenzaldehyde | 6.11E-05 | 1.22E-04 | 1.43E-04 | 1.26E-04 | 1.32E-04 | 1.38E-04 | 2.02E-04 | 7.13E-05 | 4.86E-05 | 6.70E-05 | 1.21E-04 | 8.51E-05 | 1.07E-14 | 4.46E-05 | 6.52E-05 | 1.30E-14 | 9.91E-05 | 1.08E-04 |
| 3-(4-hydroxy-3-methoxyphenyl)prop-2-enoic acid | 1.03E-04 | 1.92E-04 | 1.65E-04 | 1.80E-04 | 1.60E-04 | 1.78E-04 | 1.38E-04 | 1.15E-04 | 1.05E-04 | 1.20E-04 | 1.07E-04 | 1.32E-04 | 1.26E-04 | 9.93E-05 | 9.04E-05 | 9.41E-05 | 1.43E-04 | 1.06E-04 |
| DHBOA-Glc | 4.49E-05 | 8.34E-05 | 1.39E-04 | 1.40E-04 | 1.66E-04 | 1.29E-04 | 1.02E-04 | 1.31E-04 | 1.29E-04 | 9.54E-05 | 1.20E-04 | 1.44E-04 | 7.58E-05 | 2.00E-04 | 7.48E-05 | 1.33E-04 | 1.71E-04 | 9.83E-05 |
| Quercetin-3,7-O-alpha-L-dirhamnopyranoside | 9.61E-05 | 1.39E-04 | 1.13E-04 | 1.70E-04 | 1.70E-04 | 1.39E-04 | 2.33E-04 | 2.18E-04 | 1.65E-04 | 1.67E-04 | 1.04E-04 | 2.26E-04 | 1.87E-04 | 1.85E-04 | 1.70E-04 | 9.90E-05 | 1.48E-04 | 1.21E-04 |
| Tyrosine methylester | 7.63E-05 | 1.07E-04 | 1.16E-04 | 1.75E-04 | 1.37E-04 | 1.59E-04 | 1.45E-04 | 8.84E-05 | 1.23E-04 | 1.48E-04 | 1.99E-04 | 1.31E-04 | 8.29E-05 | 9.94E-05 | 9.59E-05 | 1.67E-04 | 5.86E-05 | 9.90E-05 |
| 3,4-Dihydroxy-trans-cinnamate | 4.45E-05 | 6.84E-05 | 1.03E-04 | 1.00E-04 | 1.06E-04 | 9.98E-05 | 1.15E-04 | 1.65E-04 | 1.02E-04 | 1.28E-04 | 1.01E-04 | 1.32E-04 | 9.19E-05 | 1.34E-04 | 8.72E-05 | 8.41E-05 | 1.67E-04 | 9.46E-05 |
| Kaempferol | 1.94E-04 | 1.88E-04 | 2.22E-04 | 1.59E-04 | 1.58E-04 | 8.22E-05 | 1.60E-04 | 2.29E-04 | 1.83E-04 | 1.73E-04 | 1.31E-14 | 1.64E-04 | 1.56E-04 | 1.42E-04 | 1.35E-04 | 1.27E-04 | 2.32E-04 | 2.25E-04 |
| Levistolide A | 2.60E-04 | 1.71E-04 | 9.52E-04 | 5.77E-05 | 3.10E-05 | 4.41E-05 | 3.04E-05 | 1.51E-04 | 2.29E-05 | 2.05E-05 | 2.71E-04 | 3.30E-05 | 2.00E-05 | 2.94E-05 | 1.36E-05 | 3.17E-05 | 2.63E-04 | 2.80E-05 |
| Deoxyinosine | 4.91E-05 | 6.88E-05 | 1.52E-04 | 1.63E-04 | 2.11E-04 | 1.64E-04 | 1.49E-04 | 1.54E-04 | 1.74E-04 | 1.08E-04 | 1.45E-04 | 1.76E-04 | 9.20E-05 | 2.77E-04 | 8.28E-05 | 1.60E-04 | 1.81E-04 | 1.05E-04 |
| Citric acid | 1.36E-04 | 1.93E-04 | 1.92E-04 | 2.48E-04 | 1.73E-04 | 2.54E-04 | 1.33E-04 | 1.98E-04 | 1.79E-04 | 2.06E-04 | 1.76E-04 | 1.99E-04 | 1.19E-04 | 2.04E-04 | 2.04E-04 | 1.89E-04 | 1.93E-04 | 1.32E-04 |
| Petroselinic acid | 8.64E-05 | 1.24E-04 | 2.04E-04 | 1.65E-04 | 1.24E-04 | 1.37E-04 | 1.76E-04 | 8.18E-05 | 9.54E-05 | 1.42E-04 | 1.23E-04 | 1.74E-04 | 1.10E-04 | 9.55E-05 | 6.63E-05 | 1.31E-04 | 1.45E-04 | 3.00E-04 |
| (6,6-Dimethylbicyclo[3.1.1]hept-2-yl)methyl 6-O-[(2R,3R,4R)-3,4-dihydroxy-4-(hydroxymethyl)tetrahydro-2-furanyl]-beta-D-glucopyranoside | 6.97E-05 | 9.60E-05 | 1.54E-04 | 3.64E-05 | 6.10E-05 | 6.17E-05 | 2.23E-04 | 2.73E-05 | 3.01E-05 | 8.15E-05 | 2.94E-04 | 3.44E-05 | 3.39E-04 | 2.49E-04 | 1.64E-04 | 3.37E-04 | 2.71E-04 | 1.64E-04 |
| Isorhamnetin 3-glucoside | 2.51E-05 | 2.60E-05 | 6.10E-05 | 1.51E-04 | 1.53E-04 | 2.44E-04 | 3.96E-04 | 9.95E-05 | 1.97E-04 | 2.68E-04 | 1.29E-04 | 3.11E-04 | 2.66E-04 | 1.34E-04 | 7.97E-05 | 1.37E-04 | 1.26E-04 | 3.62E-05 |
| 3,6,7-Trihydroxy-4'-methoxyflavone 7-rhamnoside | 3.76E-05 | 5.45E-05 | 6.92E-05 | 1.10E-04 | 1.04E-04 | 1.48E-04 | 2.51E-04 | 1.45E-04 | 2.05E-04 | 2.73E-04 | 3.50E-04 | 2.59E-04 | 1.39E-04 | 1.29E-04 | 1.23E-04 | 2.18E-04 | 2.19E-04 | 4.56E-05 |
| Taxifolin | 6.75E-05 | 1.03E-04 | 1.27E-04 | 9.97E-05 | 1.31E-04 | 6.75E-05 | 6.13E-04 | 3.13E-04 | 3.30E-04 | 2.22E-04 | 1.09E-04 | 3.34E-04 | 3.11E-04 | 2.36E-04 | 1.53E-04 | 1.16E-04 | 1.45E-04 | 1.15E-04 |
| 1H-3a,6-Epoxyazulene-7-acetic acid, octahydro-4,8a-dimethyl-alpha-methylene-1-oxo-, (3aR,4S,6S,7R,8aS)- | 2.24E-04 | 1.02E-14 | 1.19E-14 | 6.63E-05 | 5.72E-05 | 7.84E-05 | 1.17E-14 | 1.62E-04 | 9.62E-05 | 1.05E-04 | 1.16E-04 | 1.17E-04 | 1.21E-04 | 1.14E-04 | 1.10E-04 | 1.54E-04 | 1.39E-04 | 1.30E-14 |
| Vanillin | 1.31E-04 | 1.57E-04 | 1.10E-04 | 1.88E-04 | 1.40E-04 | 1.45E-04 | 1.10E-04 | 1.60E-04 | 1.59E-04 | 2.02E-04 | 1.75E-04 | 2.05E-04 | 1.18E-04 | 8.98E-05 | 1.56E-04 | 2.31E-04 | 1.31E-04 | 1.94E-04 |
| 3E,5E-tridecadienoic acid | 5.86E-05 | 9.76E-05 | 1.60E-04 | 2.87E-04 | 4.15E-04 | 3.27E-04 | 3.06E-04 | 1.44E-04 | 2.99E-04 | 1.15E-04 | 1.32E-04 | 1.58E-04 | 1.39E-04 | 2.20E-04 | 1.68E-04 | 2.48E-04 | 1.68E-04 | 1.28E-04 |
| (+)-trans-C75 | 1.44E-04 | 1.56E-04 | 1.76E-04 | 2.34E-04 | 2.24E-04 | 2.25E-04 | 1.87E-04 | 1.69E-04 | 1.94E-04 | 1.73E-04 | 1.92E-04 | 2.17E-04 | 1.83E-04 | 1.64E-04 | 1.55E-04 | 1.97E-04 | 2.06E-04 | 2.01E-04 |
| Uridine diphosphategalactose | 3.02E-05 | 9.87E-05 | 6.06E-05 | 2.10E-04 | 1.74E-04 | 2.40E-04 | 1.57E-04 | 2.70E-04 | 1.35E-04 | 3.05E-04 | 2.59E-04 | 2.04E-04 | 1.82E-04 | 1.48E-04 | 2.25E-04 | 1.70E-04 | 3.27E-04 | 3.62E-04 |
| 4-Methylumbelliferone | 7.53E-05 | 2.68E-04 | 1.64E-04 | 1.52E-04 | 7.30E-05 | 1.35E-05 | 1.45E-04 | 1.66E-04 | 1.26E-04 | 9.66E-05 | 4.24E-04 | 1.99E-04 | 3.00E-05 | 1.97E-04 | 5.10E-05 | 1.21E-04 | 2.51E-04 | 5.20E-05 |
| Daphnetin Diacetate | 1.33E-04 | 1.70E-04 | 2.28E-04 | 2.97E-04 | 1.75E-04 | 2.36E-04 | 1.39E-04 | 1.19E-04 | 1.40E-04 | 1.14E-04 | 1.11E-04 | 1.09E-04 | 9.50E-05 | 7.95E-05 | 9.79E-05 | 1.92E-04 | 1.19E-04 | 1.28E-04 |
| Gentisate aldehyde | 1.42E-04 | 1.33E-04 | 1.31E-04 | 2.65E-04 | 1.41E-04 | 1.41E-04 | 1.98E-04 | 1.61E-04 | 1.18E-04 | 1.33E-04 | 1.63E-04 | 1.08E-04 | 1.21E-04 | 7.33E-05 | 1.23E-04 | 2.09E-04 | 1.51E-04 | 3.16E-04 |
| Pantothenic acid | 1.12E-04 | 1.22E-04 | 1.36E-04 | 2.02E-04 | 1.23E-04 | 1.14E-04 | 2.60E-04 | 2.45E-04 | 1.86E-04 | 1.81E-04 | 1.64E-04 | 1.19E-04 | 2.14E-04 | 2.07E-04 | 2.65E-04 | 1.70E-04 | 2.74E-04 | 2.49E-04 |
| 3,4-Dihydroxymandelic acid | 2.78E-04 | 4.40E-04 | 2.56E-04 | 9.74E-05 | 1.32E-04 | 1.44E-04 | 4.20E-04 | 2.01E-04 | 1.55E-04 | 1.05E-04 | 1.31E-14 | 8.95E-05 | 3.32E-04 | 2.48E-04 | 2.16E-04 | 1.94E-04 | 1.06E-04 | 1.30E-04 |
| Salicylic acid | 3.76E-05 | 7.64E-05 | 1.30E-04 | 1.26E-04 | 2.47E-04 | 2.51E-04 | 2.01E-04 | 5.32E-05 | 1.31E-04 | 3.28E-04 | 8.57E-05 | 5.84E-04 | 3.91E-04 | 2.20E-04 | 1.10E-04 | 1.94E-04 | 1.49E-04 | 1.68E-04 |
| IRIGENIN | 1.37E-04 | 9.68E-05 | 1.28E-04 | 2.22E-04 | 9.52E-05 | 1.57E-04 | 1.66E-04 | 2.06E-04 | 1.26E-04 | 4.70E-04 | 2.49E-04 | 9.39E-05 | 2.28E-04 | 1.24E-04 | 2.38E-04 | 1.37E-04 | 2.46E-04 | 3.38E-04 |
| N,N'-Dicyclohexylurea | 1.68E-04 | 1.84E-04 | 1.94E-04 | 2.62E-04 | 2.42E-04 | 2.63E-04 | 2.07E-04 | 1.86E-04 | 1.94E-04 | 2.05E-04 | 2.27E-04 | 2.41E-04 | 1.79E-04 | 1.94E-04 | 1.68E-04 | 2.14E-04 | 2.32E-04 | 2.12E-04 |
| 6,10,14-Trimethyl-5,9,13-pentadecatrien-2-one | 1.11E-04 | 6.17E-05 | 2.01E-05 | 6.35E-05 | 7.21E-05 | 9.86E-05 | 3.73E-04 | 3.82E-05 | 1.35E-04 | 4.86E-04 | 1.63E-04 | 2.66E-04 | 1.72E-04 | 6.83E-05 | 1.56E-04 | 5.78E-05 | 9.16E-05 | 6.27E-05 |
| icariside B5 | 7.75E-05 | 9.61E-05 | 1.83E-04 | 3.11E-04 | 2.70E-04 | 4.59E-04 | 1.89E-04 | 7.95E-05 | 1.83E-04 | 1.57E-04 | 2.11E-04 | 2.20E-04 | 1.05E-04 | 1.06E-04 | 9.72E-05 | 1.79E-04 | 8.69E-05 | 8.18E-05 |
| 3,5,7-trihydroxy-2-(4-hydroxyphenyl)-3,4-dihydro-2H-1-benzopyran-4-one | 1.07E-04 | 1.25E-04 | 1.26E-04 | 1.86E-04 | 1.93E-04 | 1.98E-04 | 1.91E-04 | 1.30E-04 | 1.63E-04 | 2.30E-04 | 2.19E-04 | 3.50E-04 | 2.04E-04 | 1.72E-04 | 1.73E-04 | 1.77E-04 | 2.38E-04 | 1.16E-04 |
| cis-1,2-Dihydroxy-1,2-dihydro-8-carboxynaphthalene | 1.21E-04 | 1.22E-04 | 2.32E-04 | 3.03E-04 | 4.31E-04 | 3.61E-04 | 4.62E-04 | 1.17E-04 | 3.57E-04 | 1.76E-04 | 2.25E-04 | 2.68E-04 | 1.75E-04 | 1.96E-04 | 9.93E-05 | 2.78E-04 | 1.58E-04 | 1.46E-04 |
| p-Mentha-1,3,5,8-tetraene | 1.11E-04 | 1.54E-04 | 1.47E-04 | 1.29E-04 | 4.50E-04 | 1.02E-04 | 1.75E-04 | 1.20E-04 | 8.44E-05 | 1.05E-04 | 1.25E-04 | 1.45E-04 | 1.75E-04 | 1.03E-04 | 1.36E-04 | 1.13E-04 | 3.71E-04 | 1.40E-04 |
| Hexopyranose, 2-O-hexopyranosyl-6-O-[(2E)-3-(4-hydroxyphenyl)-1-oxo-2-propen-1-yl]- | 3.39E-05 | 6.32E-05 | 1.13E-04 | 1.62E-04 | 3.31E-04 | 2.41E-04 | 3.33E-04 | 2.30E-04 | 3.96E-04 | 3.31E-04 | 1.27E-04 | 5.20E-04 | 2.10E-04 | 1.65E-04 | 1.75E-04 | 1.82E-04 | 1.20E-04 | 1.13E-04 |
| Phenylacetic acid | 2.16E-04 | 2.58E-04 | 2.60E-04 | 3.14E-04 | 2.72E-04 | 2.70E-04 | 1.91E-04 | 1.60E-04 | 1.51E-04 | 1.72E-04 | 1.76E-04 | 2.15E-04 | 1.75E-04 | 1.68E-04 | 1.73E-04 | 2.10E-04 | 2.11E-04 | 2.50E-04 |
| 1-O-Feruloylglucose | 2.28E-04 | 3.73E-04 | 2.63E-04 | 3.03E-04 | 2.01E-04 | 3.05E-04 | 1.88E-04 | 2.03E-04 | 1.59E-04 | 2.14E-04 | 2.07E-04 | 1.66E-04 | 2.03E-04 | 1.53E-04 | 1.71E-04 | 1.25E-04 | 2.76E-04 | 2.08E-04 |
| 2-(3,4-dihydroxyphenyl)-5,8-dihydroxy-7-methoxy-3-[(2S,3R,4R,5R,6S)-3,4,5-trihydroxy-6-methyloxan-2-yl]oxychromen-4-one | 4.90E-04 | 2.34E-04 | 2.06E-04 | 2.42E-04 | 1.80E-04 | 1.19E-04 | 8.90E-05 | 2.30E-04 | 4.98E-05 | 6.84E-04 | 3.57E-04 | 6.44E-05 | 3.03E-04 | 1.28E-04 | 3.01E-04 | 1.25E-04 | 3.78E-04 | 1.93E-04 |
| 5,7-dihydroxy-2-(4-hydroxyphenyl)-6-[3,4,5-trihydroxy-6-(hydroxymethyl)oxan-2-yl]-4H-chromen-4-one | 2.10E-04 | 1.80E-04 | 2.31E-04 | 1.79E-04 | 3.22E-04 | 2.51E-04 | 3.16E-04 | 1.93E-04 | 2.02E-04 | 3.31E-04 | 2.03E-04 | 1.57E-04 | 2.55E-04 | 1.69E-04 | 1.67E-04 | 2.62E-04 | 1.18E-04 | 1.95E-04 |
| 2-(7-hydroxy-6-methyloctyl)-2H-furan-5-one | 8.05E-05 | 1.18E-04 | 2.84E-04 | 3.64E-04 | 3.52E-04 | 4.60E-04 | 2.44E-04 | 1.08E-04 | 2.77E-04 | 1.80E-04 | 2.39E-04 | 2.55E-04 | 1.14E-04 | 1.61E-04 | 1.27E-04 | 2.80E-04 | 1.20E-04 | 1.33E-04 |
| LAWSONE | 1.34E-04 | 2.06E-04 | 1.64E-04 | 3.34E-04 | 2.22E-04 | 1.74E-04 | 1.96E-04 | 2.61E-04 | 1.75E-04 | 3.06E-04 | 2.29E-04 | 2.98E-04 | 2.31E-04 | 2.16E-04 | 2.51E-04 | 2.02E-04 | 2.82E-04 | 2.68E-04 |
| 2(4H)-Benzofuranone, 5,6,7,7a-tetrahydro-6-hydroxy-4,4,7a-trimethyl-, (6S,7aR)- | 9.94E-05 | 1.40E-04 | 1.21E-04 | 1.76E-04 | 1.52E-04 | 1.11E-04 | 1.04E-04 | 1.26E-04 | 5.22E-05 | 7.41E-05 | 9.76E-05 | 6.50E-05 | 6.06E-05 | 1.26E-04 | 7.96E-05 | 1.30E-04 | 1.51E-04 | 1.47E-04 |
| PRALIDOXIME | 3.97E-04 | 3.14E-04 | 2.89E-04 | 2.46E-04 | 1.70E-04 | 2.01E-04 | 1.20E-04 | 2.10E-04 | 8.77E-05 | 2.65E-04 | 3.75E-04 | 1.75E-04 | 1.19E-04 | 8.79E-05 | 9.89E-05 | 2.74E-04 | 2.23E-04 | 2.01E-04 |
| (2R,3R)-2-(3,4-dihydroxyphenyl)-5,7-dihydroxy-3-[(2S,3R,4S,5S,6R)-3,4,5-trihydroxy-6-(hydroxymethyl)oxan-2-yl]oxy-2,3-dihydrochromen-4-one | 1.42E-04 | 1.62E-04 | 1.49E-04 | 1.35E-04 | 1.81E-04 | 1.42E-04 | 3.30E-04 | 4.19E-04 | 2.91E-04 | 3.27E-04 | 2.35E-04 | 2.52E-04 | 2.34E-04 | 1.91E-04 | 1.96E-04 | 2.96E-04 | 1.99E-04 | 1.95E-04 |
| D-Glucurono-6,3-lactone | 2.44E-04 | 3.21E-04 | 4.05E-04 | 5.70E-04 | 5.59E-04 | 8.14E-04 | 2.74E-04 | 2.09E-04 | 3.14E-04 | 2.23E-04 | 1.89E-04 | 2.38E-04 | 1.36E-04 | 1.45E-04 | 1.49E-04 | 2.66E-04 | 1.15E-04 | 1.94E-04 |
| Cyclopenta[c]pyran-4-carboxylic acid, 1,4a,5,7a-tetrahydro-7-(hydroxymethyl)-1-[[6-O-[(2E)-3-(4-hydroxyphenyl)-1-oxo-2-propen-1-yl]-beta-D-glucopyranosyl]oxy]-, methyl ester | 5.93E-05 | 1.16E-04 | 2.05E-04 | 3.80E-04 | 4.47E-04 | 3.50E-04 | 3.53E-04 | 2.93E-04 | 2.83E-04 | 2.77E-04 | 2.39E-04 | 3.64E-04 | 2.40E-04 | 1.37E-04 | 1.53E-04 | 2.40E-04 | 1.24E-04 | 1.12E-04 |
| 9(Z),11(E),13(E)-Octadecatrienoic Acid methyl ester | 2.61E-04 | 1.43E-04 | 8.12E-05 | 2.58E-04 | 2.17E-04 | 4.36E-04 | 2.78E-04 | 3.80E-05 | 1.96E-04 | 7.34E-04 | 2.13E-04 | 3.70E-04 | 3.98E-04 | 2.49E-04 | 4.24E-04 | 1.05E-04 | 1.57E-04 | 1.12E-04 |
| Myricetin-3-Galactoside | 3.28E-04 | 2.77E-04 | 2.03E-04 | 2.48E-04 | 2.34E-04 | 2.20E-04 | 2.71E-04 | 2.42E-04 | 1.82E-04 | 2.37E-04 | 1.54E-04 | 2.72E-04 | 2.66E-04 | 2.88E-04 | 3.44E-04 | 2.43E-04 | 2.77E-04 | 2.26E-04 |
| Laricitrin 3-glucoside | 2.38E-04 | 8.49E-05 | 1.44E-04 | 1.07E-04 | 1.72E-04 | 1.50E-04 | 4.29E-04 | 2.47E-04 | 4.05E-04 | 4.61E-04 | 2.47E-04 | 3.65E-04 | 6.73E-04 | 4.64E-04 | 2.26E-04 | 3.41E-04 | 3.38E-04 | 6.06E-05 |
| Chlorogenic acid | 3.93E-04 | 4.77E-04 | 2.60E-04 | 3.45E-04 | 3.05E-04 | 3.53E-04 | 2.41E-04 | 8.28E-04 | 1.87E-04 | 2.70E-04 | 2.26E-04 | 1.92E-04 | 2.06E-04 | 3.10E-04 | 4.91E-04 | 2.40E-04 | 4.37E-04 | 2.47E-04 |
| 5-[6-(3-hydroxy-4-methoxyphenyl)-1,3,3a,4,6,6a-hexahydrofuro[3,4-c]furan-3-yl]-2-methoxyphenol | 5.30E-04 | 1.97E-04 | 5.36E-04 | 3.20E-04 | 2.38E-04 | 2.37E-04 | 1.94E-04 | 2.78E-04 | 3.81E-04 | 1.46E-04 | 2.81E-04 | 1.75E-04 | 1.97E-04 | 3.73E-04 | 3.18E-04 | 4.55E-04 | 1.28E-04 | 9.96E-05 |
| QUERCETIN TETRAMETHYL (5,7,3',4') ETHER | 2.79E-04 | 2.54E-04 | 2.21E-04 | 7.94E-05 | 1.79E-04 | 1.34E-04 | 3.41E-04 | 4.48E-04 | 2.65E-04 | 6.19E-04 | 3.30E-04 | 9.38E-05 | 1.99E-04 | 1.90E-04 | 3.39E-04 | 2.34E-04 | 1.11E-04 | 7.46E-05 |
| Symmetric dimethylarginine | 2.77E-04 | 2.61E-04 | 4.35E-04 | 5.85E-04 | 3.29E-04 | 8.36E-04 | 4.95E-04 | 1.91E-04 | 4.13E-04 | 1.67E-04 | 3.93E-04 | 9.89E-05 | 1.32E-04 | 1.41E-04 | 1.19E-04 | 1.60E-04 | 1.67E-04 | 9.58E-05 |
| Ambonone | 1.50E-04 | 3.33E-04 | 4.62E-04 | 5.31E-04 | 3.54E-04 | 5.26E-04 | 1.63E-04 | 1.72E-04 | 1.41E-04 | 1.53E-04 | 1.93E-04 | 1.34E-04 | 8.45E-05 | 6.63E-05 | 8.58E-05 | 1.87E-04 | 1.09E-04 | 1.33E-04 |
| Dihydrocalythropsin | 1.15E-04 | 2.37E-04 | 1.01E-04 | 2.46E-04 | 7.77E-05 | 1.06E-04 | 1.02E-04 | 1.65E-04 | 4.53E-05 | 6.71E-04 | 1.29E-04 | 8.08E-04 | 8.49E-04 | 3.60E-04 | 5.25E-04 | 2.02E-04 | 3.41E-04 | 4.63E-04 |
| Miscanthoside | 1.12E-04 | 1.44E-04 | 1.32E-04 | 2.38E-04 | 2.10E-04 | 2.53E-04 | 4.49E-04 | 2.37E-04 | 3.04E-04 | 4.60E-04 | 2.23E-04 | 6.56E-04 | 2.91E-04 | 3.62E-04 | 2.43E-04 | 3.18E-04 | 3.13E-04 | 2.49E-04 |
| 21-hydroxyallopregnanolone | 1.97E-04 | 1.81E-04 | 3.05E-04 | 3.71E-04 | 2.27E-04 | 2.95E-04 | 2.44E-04 | 3.69E-04 | 1.93E-04 | 4.89E-04 | 4.16E-04 | 1.32E-04 | 5.15E-04 | 2.25E-04 | 2.49E-04 | 1.28E-04 | 3.48E-04 | 2.53E-04 |
| Bergamottin | 2.58E-04 | 2.92E-04 | 3.43E-04 | 4.92E-04 | 5.42E-04 | 7.14E-04 | 2.92E-04 | 2.65E-04 | 4.08E-04 | 2.21E-04 | 2.35E-04 | 1.96E-04 | 2.00E-04 | 1.80E-04 | 3.93E-04 | 3.79E-04 | 1.67E-04 | 2.06E-04 |
| Gly Glu Asp | 4.97E-05 | 5.79E-05 | 2.53E-05 | 1.88E-04 | 2.64E-04 | 1.45E-04 | 5.23E-04 | 2.30E-04 | 5.56E-04 | 4.12E-04 | 1.80E-05 | 7.35E-04 | 5.66E-04 | 3.51E-04 | 5.53E-04 | 1.78E-04 | 2.18E-04 | 1.64E-04 |
| RESISTOMYCIN | 6.40E-05 | 1.01E-04 | 2.15E-04 | 1.24E-04 | 1.44E-04 | 1.93E-04 | 2.04E-04 | 1.05E-04 | 1.15E-04 | 2.08E-04 | 1.62E-04 | 1.90E-04 | 1.38E-04 | 6.78E-05 | 9.54E-05 | 2.51E-04 | 8.24E-05 | 1.63E-04 |
| Pinolenic Acid | 2.29E-04 | 1.15E-04 | 9.58E-05 | 2.47E-04 | 2.20E-04 | 4.75E-04 | 3.38E-04 | 3.34E-05 | 2.45E-04 | 6.70E-04 | 2.80E-04 | 3.70E-04 | 3.29E-04 | 1.92E-04 | 4.23E-04 | 1.30E-04 | 1.53E-04 | 8.89E-05 |
| Gentisic acid | 4.12E-04 | 5.90E-04 | 5.85E-04 | 7.11E-04 | 4.82E-04 | 8.11E-04 | 2.84E-04 | 1.47E-04 | 1.77E-04 | 3.05E-04 | 3.42E-04 | 2.52E-04 | 3.08E-04 | 2.33E-04 | 2.38E-04 | 4.58E-04 | 2.72E-04 | 2.65E-04 |
| Lauroyl diethanolamide | 2.05E-04 | 2.68E-04 | 3.22E-04 | 3.56E-04 | 3.06E-04 | 3.62E-04 | 3.04E-04 | 2.22E-04 | 2.65E-04 | 3.06E-04 | 3.07E-04 | 3.42E-04 | 2.94E-04 | 2.41E-04 | 2.27E-04 | 3.45E-04 | 3.04E-04 | 3.37E-04 |
| Tetralin | 1.68E-04 | 2.64E-04 | 2.95E-04 | 3.48E-04 | 3.70E-04 | 3.19E-04 | 3.76E-04 | 3.56E-04 | 3.46E-04 | 4.04E-04 | 6.14E-04 | 4.77E-04 | 3.14E-04 | 3.16E-04 | 3.04E-04 | 3.22E-04 | 4.44E-04 | 4.00E-04 |
| avicularin | 3.62E-03 | 2.37E-03 | 4.53E-04 | 2.60E-05 | 5.39E-05 | 1.15E-05 | 1.36E-04 | 4.51E-04 | 9.57E-05 | 1.56E-05 | 1.54E-05 | 2.33E-05 | 1.18E-03 | 3.03E-04 | 4.14E-04 | 4.51E-05 | 7.01E-05 | 4.10E-05 |
| Coumaroylquinic acid | 3.35E-04 | 3.93E-04 | 4.82E-04 | 8.97E-04 | 6.23E-04 | 9.69E-04 | 4.65E-04 | 2.50E-04 | 3.54E-04 | 3.32E-04 | 2.91E-04 | 2.62E-04 | 3.13E-04 | 1.72E-04 | 2.55E-04 | 4.39E-04 | 2.88E-04 | 3.06E-04 |
| FRAXETIN | 1.35E-04 | 1.51E-04 | 3.05E-04 | 4.72E-04 | 5.57E-04 | 6.35E-04 | 6.12E-04 | 2.00E-04 | 7.77E-04 | 3.04E-04 | 5.06E-04 | 3.80E-04 | 2.22E-04 | 2.45E-04 | 1.96E-04 | 5.37E-04 | 2.60E-04 | 2.75E-04 |
| 2-keto-D-Gluconic acid | 3.84E-04 | 3.00E-04 | 2.57E-04 | 3.66E-04 | 1.29E-04 | 7.92E-05 | 2.71E-04 | 5.15E-04 | 2.33E-04 | 3.45E-04 | 4.91E-04 | 2.62E-04 | 3.03E-04 | 3.59E-04 | 5.33E-04 | 2.90E-04 | 5.04E-04 | 5.42E-04 |
| Ginkgolide A | 1.48E-04 | 2.39E-04 | 2.62E-04 | 3.72E-04 | 3.99E-04 | 3.67E-04 | 2.82E-04 | 1.64E-04 | 2.25E-04 | 2.19E-04 | 1.83E-04 | 1.70E-04 | 1.61E-04 | 2.03E-04 | 1.96E-04 | 3.10E-04 | 1.85E-04 | 2.62E-04 |
| 2,6-Dimethyl-2,4E,6E-octatriene | 1.46E-04 | 1.86E-04 | 3.64E-04 | 3.19E-04 | 7.34E-04 | 6.43E-04 | 6.77E-04 | 2.45E-04 | 7.63E-04 | 1.95E-04 | 3.14E-04 | 3.13E-04 | 2.18E-04 | 2.57E-04 | 2.01E-04 | 4.71E-04 | 8.61E-05 | 2.14E-04 |
| Morin | 2.32E-04 | 3.24E-04 | 2.61E-04 | 4.11E-04 | 3.60E-04 | 3.39E-04 | 4.40E-04 | 3.85E-04 | 3.30E-04 | 3.96E-04 | 2.70E-04 | 3.94E-04 | 3.64E-04 | 3.71E-04 | 3.05E-04 | 2.73E-04 | 3.57E-04 | 2.86E-04 |
| 7-Hydroxy-6-methyl-2H-1-benzopyran-2-one | 1.45E-04 | 1.71E-04 | 2.69E-04 | 2.04E-04 | 2.07E-04 | 1.51E-04 | 1.66E-04 | 1.69E-04 | 1.73E-04 | 1.53E-04 | 1.63E-04 | 1.58E-04 | 1.37E-04 | 2.35E-04 | 1.24E-04 | 1.44E-04 | 2.63E-04 | 4.69E-03 |
| (卤)5(6)-EET methyl ester | 1.76E-04 | 2.34E-04 | 3.42E-04 | 4.46E-04 | 2.84E-04 | 4.22E-04 | 2.79E-04 | 4.02E-04 | 2.39E-04 | 5.47E-04 | 5.73E-04 | 1.51E-04 | 5.67E-04 | 2.71E-04 | 2.64E-04 | 1.50E-04 | 4.41E-04 | 2.80E-04 |
| Scoparone | 1.55E-04 | 2.12E-04 | 2.28E-04 | 5.53E-04 | 6.36E-04 | 4.35E-04 | 6.80E-04 | 3.93E-04 | 3.95E-04 | 3.44E-04 | 3.66E-04 | 6.57E-04 | 3.84E-04 | 3.40E-04 | 4.05E-04 | 2.85E-04 | 3.68E-04 | 1.65E-04 |
| [3,4,5-trihydroxy-6-[(3,4,5-trihydroxyoxan-2-yl)oxymethyl]oxan-2-yl] 2,6,6-trimethylcyclohexene-1-carboxylate | 1.00E-04 | 1.23E-04 | 4.18E-04 | 3.58E-04 | 6.06E-04 | 5.83E-04 | 7.74E-04 | 2.56E-04 | 9.17E-04 | 3.86E-04 | 5.10E-04 | 5.81E-04 | 4.21E-04 | 4.40E-04 | 2.11E-04 | 4.69E-04 | 2.03E-04 | 1.98E-04 |
| 2-Butanone, 4-[3-(beta-D-glucopyranosyloxy)-4-hydroxy-2,6,6-trimethyl-1-cyclohexen-1-yl]- | 1.48E-04 | 2.45E-04 | 4.86E-04 | 3.33E-04 | 7.88E-04 | 8.52E-04 | 9.96E-04 | 3.18E-04 | 1.01E-03 | 2.71E-04 | 4.10E-04 | 3.97E-04 | 2.29E-04 | 2.98E-04 | 1.94E-04 | 4.86E-04 | 1.06E-04 | 2.15E-04 |
| Erbstatin Analog | 2.96E-04 | 4.78E-04 | 6.57E-04 | 1.00E-03 | 1.46E-03 | 1.31E-03 | 4.70E-04 | 4.08E-04 | 6.97E-04 | 3.96E-04 | 2.83E-04 | 4.65E-04 | 1.41E-04 | 1.82E-04 | 2.42E-04 | 2.69E-04 | 2.25E-04 | 1.70E-04 |
| Kaempferol glucuronide | 2.36E-04 | 2.81E-04 | 5.02E-04 | 2.94E-04 | 6.42E-04 | 4.52E-04 | 5.05E-04 | 5.76E-04 | 7.12E-04 | 4.45E-04 | 3.27E-04 | 5.23E-04 | 8.00E-04 | 1.14E-03 | 5.80E-04 | 7.27E-04 | 4.31E-04 | 3.47E-04 |
| 2-Hydroxy-8-methylchromene-2-carboxylate | 3.65E-04 | 4.46E-04 | 3.67E-04 | 4.87E-04 | 7.51E-04 | 5.23E-04 | 6.58E-04 | 5.93E-04 | 4.97E-04 | 3.81E-04 | 3.35E-04 | 4.64E-04 | 4.44E-04 | 3.33E-04 | 4.43E-04 | 2.99E-04 | 5.33E-04 | 4.11E-04 |
| 2'-Hydroxyacetophenone | 2.16E-04 | 3.08E-04 | 5.13E-04 | 7.96E-04 | 1.10E-03 | 1.03E-03 | 5.99E-04 | 4.69E-04 | 1.18E-03 | 5.10E-04 | 3.21E-04 | 6.61E-04 | 2.61E-04 | 3.46E-04 | 3.03E-04 | 5.23E-04 | 2.90E-04 | 2.73E-04 |
| Herniarin | 4.13E-04 | 3.78E-04 | 5.90E-04 | 5.86E-04 | 7.12E-04 | 7.64E-04 | 6.20E-04 | 2.86E-04 | 5.79E-04 | 3.21E-04 | 5.08E-04 | 5.77E-04 | 3.03E-04 | 3.87E-04 | 2.47E-04 | 6.06E-04 | 4.08E-04 | 4.36E-04 |
| 6,2'4'-Trihydroxy-2-phenylbenzofuran | 1.77E-04 | 2.64E-04 | 3.75E-04 | 4.16E-04 | 5.09E-04 | 2.37E-04 | 4.86E-04 | 4.14E-04 | 6.10E-04 | 3.90E-04 | 3.46E-04 | 7.26E-04 | 4.89E-04 | 3.36E-04 | 3.17E-04 | 6.18E-04 | 4.37E-04 | 3.80E-04 |
| 1-O-Feruloyl-尾-D-glucose | 5.54E-04 | 8.54E-04 | 6.55E-04 | 7.95E-04 | 7.23E-04 | 7.95E-04 | 5.37E-04 | 5.32E-04 | 4.76E-04 | 4.83E-04 | 4.33E-04 | 5.00E-04 | 5.29E-04 | 3.82E-04 | 4.65E-04 | 4.53E-04 | 6.74E-04 | 5.06E-04 |
| 1-hydroxy-4-[(2S,3R,4S,5S,6R)-3,4,5-trihydroxy-6-(hydroxymethyl)oxan-2-yl]oxynaphthalene-2-carboxylic acid | 2.91E-04 | 4.50E-04 | 5.88E-04 | 4.68E-04 | 4.63E-04 | 5.89E-04 | 5.40E-04 | 6.19E-04 | 5.15E-04 | 7.07E-04 | 5.19E-04 | 9.19E-04 | 5.33E-04 | 3.75E-04 | 4.58E-04 | 5.78E-04 | 4.58E-04 | 7.11E-04 |
| Nevadensin | 5.97E-04 | 4.91E-04 | 3.50E-04 | 2.31E-04 | 3.76E-04 | 3.19E-04 | 3.03E-04 | 4.54E-04 | 2.32E-04 | 9.17E-04 | 6.57E-04 | 1.53E-04 | 6.17E-04 | 3.98E-04 | 8.39E-04 | 5.16E-04 | 5.13E-04 | 5.93E-04 |
| Tamarixetin | 8.78E-04 | 5.45E-04 | 3.83E-04 | 5.38E-04 | 2.82E-04 | 2.74E-04 | 6.09E-04 | 7.35E-04 | 3.86E-04 | 7.85E-04 | 5.19E-04 | 4.11E-04 | 1.07E-03 | 5.85E-04 | 9.09E-04 | 4.98E-04 | 7.92E-04 | 1.04E-03 |
| MG(18:2(9Z,12Z)/0:0/0:0) | 4.20E-04 | 1.99E-04 | 7.90E-05 | 2.31E-04 | 2.74E-04 | 3.58E-04 | 8.41E-04 | 1.08E-04 | 3.82E-04 | 1.43E-03 | 7.00E-04 | 6.32E-04 | 4.94E-04 | 1.51E-04 | 5.09E-04 | 1.66E-04 | 3.88E-04 | 1.69E-04 |
| Valine | 6.50E-04 | 9.85E-04 | 7.31E-04 | 1.21E-03 | 6.43E-04 | 7.89E-04 | 7.33E-04 | 7.58E-04 | 1.22E-03 | 8.62E-04 | 7.29E-04 | 6.86E-04 | 5.68E-04 | 5.23E-04 | 6.30E-04 | 4.10E-04 | 1.08E-03 | 5.84E-04 |
| 4-phenyl-5-methyl-1,2,3-Thiadiazole | 8.81E-15 | 1.02E-14 | 1.19E-14 | 6.60E-04 | 6.02E-04 | 9.80E-04 | 5.63E-04 | 5.57E-04 | 1.06E-14 | 5.15E-04 | 1.31E-14 | 4.78E-04 | 4.71E-04 | 1.11E-03 | 3.94E-04 | 1.30E-14 | 6.34E-04 | 1.54E-03 |
| 6-[5]-ladderane-1-hexanol | 4.65E-04 | 2.58E-04 | 1.45E-04 | 3.88E-04 | 3.87E-04 | 8.52E-04 | 6.39E-04 | 6.84E-05 | 3.88E-04 | 1.27E-03 | 4.60E-04 | 6.27E-04 | 5.83E-04 | 3.75E-04 | 6.59E-04 | 2.36E-04 | 2.66E-04 | 1.76E-04 |
| Kynurenic acid | 2.84E-04 | 3.85E-04 | 5.12E-04 | 5.64E-04 | 9.06E-04 | 8.59E-04 | 8.79E-04 | 7.73E-04 | 8.16E-04 | 7.00E-04 | 8.63E-04 | 6.44E-04 | 4.58E-04 | 5.44E-04 | 4.77E-04 | 7.03E-04 | 5.77E-04 | 6.85E-04 |
| Indanone | 3.83E-04 | 4.99E-04 | 5.87E-04 | 3.51E-04 | 4.76E-04 | 3.89E-04 | 2.88E-04 | 2.81E-04 | 3.83E-04 | 5.17E-04 | 3.68E-04 | 4.83E-04 | 2.86E-04 | 2.89E-04 | 2.77E-04 | 4.36E-04 | 3.41E-04 | 6.39E-04 |
| Fluorescein | 1.53E-04 | 2.04E-04 | 3.59E-04 | 3.48E-04 | 7.02E-04 | 5.03E-04 | 4.08E-04 | 2.43E-04 | 2.79E-04 | 3.09E-04 | 3.85E-04 | 2.44E-04 | 1.59E-04 | 1.61E-04 | 1.39E-04 | 4.11E-04 | 4.09E-04 | 1.80E-04 |
| xi-3-(4-Isopropylphenyl)-2-methylpropanal | 2.29E-04 | 3.15E-04 | 6.75E-04 | 5.86E-04 | 1.21E-03 | 9.96E-04 | 1.30E-03 | 3.82E-04 | 1.34E-03 | 3.19E-04 | 5.66E-04 | 4.79E-04 | 3.50E-04 | 4.20E-04 | 3.06E-04 | 6.48E-04 | 1.37E-04 | 3.84E-04 |
| 2-Hydroxycinnamic acid | 5.56E-05 | 6.39E-05 | 6.24E-05 | 4.34E-05 | 7.54E-04 | 5.86E-05 | 7.08E-05 | 7.88E-05 | 6.24E-05 | 1.30E-03 | 1.76E-03 | 1.25E-03 | 6.46E-05 | 4.73E-05 | 5.25E-05 | 1.69E-03 | 1.20E-03 | 1.37E-03 |
| 5,7-dihydroxy-2-(4-hydroxy-3,5-dimethoxyphenyl)-4H-chromen-4-one | 4.71E-04 | 2.80E-04 | 3.25E-04 | 5.41E-04 | 3.48E-04 | 6.55E-04 | 5.32E-04 | 3.54E-04 | 3.93E-04 | 8.49E-04 | 6.05E-04 | 2.55E-04 | 1.27E-03 | 4.23E-04 | 5.44E-04 | 5.09E-04 | 6.58E-04 | 1.43E-03 |
| Dopamine | 1.02E-03 | 8.46E-04 | 1.91E-03 | 2.88E-04 | 4.38E-05 | 7.62E-05 | 1.73E-05 | 6.83E-05 | 9.72E-05 | 7.86E-05 | 2.68E-03 | 2.60E-04 | 4.48E-05 | 1.38E-05 | 4.72E-06 | 3.20E-04 | 3.23E-04 | 6.55E-05 |
| N-methylundec-10-enamide | 5.22E-04 | 6.54E-04 | 7.05E-04 | 8.03E-04 | 7.73E-04 | 8.69E-04 | 6.27E-04 | 5.99E-04 | 6.14E-04 | 6.67E-04 | 7.55E-04 | 7.32E-04 | 6.11E-04 | 6.13E-04 | 5.71E-04 | 7.47E-04 | 7.47E-04 | 7.48E-04 |
| 4-Guanidinobutanoic acid | 4.89E-04 | 3.82E-04 | 3.05E-04 | 1.55E-03 | 6.44E-04 | 5.45E-04 | 4.79E-04 | 4.16E-04 | 8.73E-04 | 5.57E-04 | 1.17E-03 | 7.25E-04 | 4.97E-04 | 8.58E-04 | 4.20E-04 | 5.40E-04 | 8.23E-04 | 1.81E-03 |
| Fisetin | 6.91E-04 | 6.62E-04 | 5.54E-04 | 9.20E-04 | 9.65E-04 | 8.80E-04 | 7.14E-04 | 4.48E-04 | 7.82E-04 | 7.80E-04 | 1.17E-03 | 6.42E-04 | 6.00E-04 | 7.87E-04 | 7.82E-04 | 5.68E-04 | 5.56E-04 | 3.68E-04 |
| Triethyl citrate | 4.20E-04 | 5.17E-04 | 6.89E-04 | 7.92E-04 | 6.56E-04 | 6.67E-04 | 6.58E-04 | 5.12E-04 | 5.39E-04 | 6.20E-04 | 9.22E-04 | 7.38E-04 | 5.74E-04 | 5.12E-04 | 4.98E-04 | 6.72E-04 | 6.09E-04 | 7.27E-04 |
| Styrene | 5.22E-04 | 5.30E-04 | 5.23E-04 | 7.59E-04 | 8.46E-04 | 9.25E-04 | 6.19E-04 | 4.96E-04 | 5.67E-04 | 5.49E-04 | 5.32E-04 | 6.41E-04 | 6.16E-04 | 5.15E-04 | 5.56E-04 | 5.91E-04 | 8.39E-04 | 5.55E-04 |
| 2-(3,4-dihydroxyphenyl)-5,7-dihydroxy-6,8-dimethoxy-4H-chromen-4-one | 9.11E-04 | 5.60E-04 | 3.75E-04 | 4.51E-04 | 3.84E-04 | 4.25E-04 | 4.13E-04 | 6.50E-04 | 4.47E-04 | 1.39E-03 | 5.75E-04 | 2.90E-04 | 1.40E-03 | 5.00E-04 | 9.78E-04 | 4.48E-04 | 6.79E-04 | 2.04E-03 |
| 4-O-Demethyl-13-dihydroadriamycinone | 5.98E-04 | 6.78E-04 | 6.73E-04 | 9.85E-04 | 9.19E-04 | 9.95E-04 | 8.28E-04 | 7.16E-04 | 7.79E-04 | 7.07E-04 | 7.69E-04 | 9.11E-04 | 6.65E-04 | 7.24E-04 | 6.09E-04 | 8.34E-04 | 8.25E-04 | 8.72E-04 |
| PC(16:0/0:0)[U] / PC(16:0/0:0)[rac] | 2.35E-04 | 3.10E-04 | 4.98E-04 | 6.60E-04 | 3.43E-04 | 3.91E-04 | 6.79E-04 | 6.97E-04 | 5.47E-04 | 8.13E-04 | 8.86E-04 | 7.64E-04 | 4.79E-04 | 4.24E-04 | 5.61E-04 | 5.22E-04 | 7.33E-04 | 5.46E-04 |
| Spiro[cyclopenta[c]pyran-7(1H),2'(5'H)-furan]-4-carboxylic acid, 1-(beta-D-glucopyranosyloxy)-4a,7a-dihydro-4'-[(1S)-1-[[(2E)-3-(4-hydroxyphenyl)-1-oxo-2-propen-1-yl]oxy]ethyl]-5'-oxo-, methyl ester, (1S,4aS,7R,7aS)- | 2.57E-04 | 5.91E-04 | 6.19E-04 | 9.75E-04 | 1.08E-03 | 8.40E-04 | 6.63E-04 | 9.96E-04 | 7.52E-04 | 1.22E-03 | 4.15E-04 | 1.37E-03 | 1.06E-03 | 8.11E-04 | 9.99E-04 | 6.78E-04 | 7.06E-04 | 6.70E-04 |
| 1-Oxo-1H-2-benzopyran-3-carboxaldehyde | 4.27E-04 | 4.60E-04 | 4.59E-04 | 8.99E-04 | 8.55E-04 | 1.02E-03 | 6.25E-04 | 5.60E-04 | 4.80E-04 | 6.19E-04 | 6.81E-04 | 6.50E-04 | 5.50E-04 | 4.84E-04 | 5.19E-04 | 5.97E-04 | 6.00E-04 | 6.36E-04 |
| 7-methoxy-6-[(2S,3R,4S,5S,6R)-3,4,5-trihydroxy-6-(hydroxymethyl)oxan-2-yl]oxychromen-2-one | 5.39E-04 | 1.11E-03 | 7.97E-04 | 8.54E-04 | 6.45E-04 | 4.93E-04 | 7.85E-04 | 7.50E-04 | 7.04E-04 | 1.04E-03 | 8.95E-04 | 7.20E-04 | 9.70E-04 | 7.04E-04 | 7.50E-04 | 5.75E-04 | 1.03E-03 | 8.26E-04 |
| 2-Linoleoyl Glycerol | 4.72E-04 | 2.49E-04 | 9.43E-05 | 3.18E-04 | 3.49E-04 | 4.87E-04 | 1.66E-03 | 1.35E-04 | 6.45E-04 | 2.24E-03 | 6.86E-04 | 1.15E-03 | 6.93E-04 | 2.98E-04 | 7.76E-04 | 2.68E-04 | 4.30E-04 | 2.58E-04 |
| Myricetin 3-glucuronide | 5.19E-04 | 6.45E-04 | 5.88E-04 | 5.63E-04 | 6.97E-04 | 7.02E-04 | 1.12E-03 | 9.23E-04 | 9.60E-04 | 6.15E-04 | 6.05E-04 | 8.45E-04 | 1.07E-03 | 1.17E-03 | 8.60E-04 | 9.88E-04 | 8.66E-04 | 9.60E-04 |
| 6-hydroxysphingosine | 8.36E-04 | 6.69E-04 | 9.24E-04 | 1.07E-03 | 7.45E-04 | 4.63E-04 | 7.35E-04 | 4.22E-04 | 4.52E-04 | 6.67E-04 | 5.33E-04 | 4.31E-04 | 5.65E-04 | 4.78E-04 | 6.02E-04 | 4.87E-04 | 7.35E-04 | 6.54E-04 |
| Quercetin | 8.94E-04 | 7.53E-04 | 6.41E-04 | 1.14E-03 | 9.98E-04 | 8.60E-04 | 1.12E-03 | 5.85E-04 | 8.08E-04 | 8.40E-04 | 7.20E-04 | 1.11E-03 | 1.12E-03 | 9.48E-04 | 8.22E-04 | 4.53E-04 | 5.82E-04 | 3.39E-04 |
| Naphtho[2,3-b]furan-9(4H)-one, 4,8-bis(acetyloxy)-4a,5,6,7,8,8a-hexahydro-3,4a,5-trimethyl-, (4S,4aR,5S,8S,8aS)- | 2.64E-04 | 2.95E-04 | 4.15E-04 | 8.24E-04 | 6.61E-04 | 4.94E-04 | 1.13E-03 | 4.73E-04 | 6.49E-04 | 1.13E-03 | 1.54E-03 | 1.34E-03 | 6.10E-04 | 4.51E-04 | 4.27E-04 | 8.45E-04 | 3.95E-04 | 2.94E-04 |
| 2-(hydroxymethyl)-6-[5-[3-(hydroxymethyl)-5-(3-hydroxypropyl)-7-methoxy-2,3-dihydro-1-benzofuran-2-yl]-2-methoxyphenoxy]oxane-3,4,5-triol | 1.77E-04 | 3.24E-04 | 3.20E-04 | 5.26E-04 | 3.66E-04 | 3.85E-04 | 1.02E-03 | 7.10E-04 | 5.54E-04 | 8.45E-04 | 8.31E-04 | 9.00E-04 | 3.83E-04 | 3.02E-04 | 4.72E-04 | 1.41E-03 | 7.50E-04 | 1.13E-03 |
| Enol-phenylpyruvate | 2.88E-04 | 5.13E-04 | 5.19E-04 | 1.08E-03 | 9.99E-04 | 8.08E-04 | 1.16E-03 | 1.05E-03 | 1.11E-03 | 1.33E-03 | 7.44E-04 | 1.56E-03 | 1.01E-03 | 8.49E-04 | 8.25E-04 | 7.72E-04 | 7.08E-04 | 8.80E-04 |
| 2-Caffeoylisocitrate | 1.36E-03 | 1.61E-03 | 7.99E-04 | 1.14E-03 | 1.06E-03 | 9.10E-04 | 7.08E-04 | 2.71E-03 | 6.21E-04 | 7.13E-04 | 8.01E-04 | 6.82E-04 | 8.00E-04 | 8.26E-04 | 1.47E-03 | 6.99E-04 | 1.24E-03 | 9.02E-04 |
| (3beta,5xi,9xi,13alpha,17alpha,18xi)-3-Hydroxy-13,28-epoxyurs-11-en-28-one | 6.23E-04 | 7.22E-04 | 8.23E-04 | 9.48E-04 | 8.52E-04 | 9.22E-04 | 7.44E-04 | 6.42E-04 | 7.98E-04 | 9.75E-04 | 9.79E-04 | 9.16E-04 | 6.90E-04 | 6.93E-04 | 7.16E-04 | 7.53E-04 | 8.63E-04 | 1.02E-03 |
| myo-Inositol | 6.42E-04 | 7.36E-04 | 9.65E-04 | 1.06E-03 | 1.06E-03 | 1.18E-03 | 9.00E-04 | 8.72E-04 | 8.99E-04 | 8.27E-04 | 1.10E-03 | 9.69E-04 | 6.87E-04 | 6.48E-04 | 5.91E-04 | 1.04E-03 | 6.77E-04 | 8.52E-04 |
| Casticin | 5.55E-04 | 4.52E-04 | 3.98E-04 | 6.78E-04 | 4.79E-04 | 5.20E-04 | 1.21E-03 | 9.80E-04 | 5.92E-04 | 2.19E-03 | 9.91E-04 | 6.40E-04 | 1.88E-03 | 5.96E-04 | 1.02E-03 | 8.73E-04 | 1.41E-03 | 7.09E-04 |
| Gingerglycolipid A | 6.68E-04 | 4.49E-04 | 1.78E-04 | 5.84E-04 | 6.96E-04 | 1.35E-03 | 1.17E-03 | 1.12E-04 | 9.14E-04 | 1.73E-03 | 8.17E-04 | 1.16E-03 | 1.05E-03 | 5.80E-04 | 9.89E-04 | 3.69E-04 | 4.41E-04 | 2.63E-04 |
| Cinnamaldehyde | 1.04E-03 | 1.36E-03 | 1.40E-03 | 9.12E-04 | 9.55E-04 | 6.42E-04 | 7.06E-04 | 1.03E-03 | 9.61E-04 | 1.01E-03 | 1.18E-03 | 1.09E-03 | 7.38E-04 | 6.75E-04 | 7.41E-04 | 7.06E-04 | 9.23E-04 | 1.10E-03 |
| beta-Ionone | 8.34E-04 | 9.29E-04 | 1.06E-03 | 1.37E-03 | 1.26E-03 | 1.33E-03 | 1.04E-03 | 9.30E-04 | 9.84E-04 | 1.02E-03 | 1.15E-03 | 1.20E-03 | 9.74E-04 | 9.44E-04 | 8.50E-04 | 1.18E-03 | 1.23E-03 | 1.20E-03 |
| Melibiose | 8.93E-04 | 1.09E-03 | 1.72E-03 | 1.91E-03 | 2.13E-03 | 2.23E-03 | 1.54E-03 | 8.97E-04 | 1.40E-03 | 1.45E-03 | 1.26E-03 | 1.39E-03 | 1.49E-03 | 1.29E-03 | 1.17E-03 | 1.63E-03 | 1.70E-03 | 1.28E-03 |
| D-Apiose | 9.01E-04 | 8.64E-04 | 5.99E-04 | 1.36E-03 | 1.06E-03 | 9.40E-04 | 9.99E-04 | 6.60E-04 | 7.74E-04 | 1.32E-03 | 5.34E-04 | 7.38E-04 | 7.82E-04 | 8.72E-04 | 9.40E-04 | 1.09E-03 | 9.86E-04 | 8.93E-04 |
| Coumaric acid | 2.80E-04 | 1.02E-14 | 1.19E-14 | 1.44E-14 | 1.32E-14 | 1.89E-03 | 1.27E-03 | 4.14E-04 | 1.31E-03 | 1.12E-14 | 1.31E-14 | 1.40E-03 | 1.28E-03 | 1.09E-03 | 7.64E-04 | 9.95E-04 | 2.74E-04 | 3.13E-04 |
| C10-LAS (SAMPLE) | 3.19E-04 | 2.52E-04 | 2.91E-04 | 1.59E-04 | 7.92E-05 | 1.54E-05 | 1.57E-04 | 1.59E-04 | 1.73E-04 | 1.42E-04 | 3.81E-03 | 1.57E-04 | 3.35E-05 | 1.00E-14 | 2.21E-04 | 2.29E-04 | 2.66E-04 | 7.71E-04 |
| Guanosine | 1.67E-03 | 1.57E-03 | 1.15E-03 | 9.69E-04 | 5.51E-04 | 3.60E-04 | 7.20E-04 | 1.24E-03 | 5.10E-04 | 1.04E-03 | 1.62E-03 | 7.93E-04 | 5.85E-04 | 4.49E-04 | 6.29E-04 | 7.51E-04 | 9.78E-04 | 1.05E-03 |
| (3R,4R,5R)-5-[(E)-3-(3,4-dihydroxyphenyl)prop-2-enoyl]oxy-3,4-dihydroxycyclohexene-1-carboxylic acid | 1.39E-03 | 1.57E-03 | 1.11E-03 | 1.43E-03 | 1.52E-03 | 1.23E-03 | 9.71E-04 | 1.72E-03 | 7.17E-04 | 7.92E-04 | 7.79E-04 | 7.65E-04 | 8.36E-04 | 8.07E-04 | 1.52E-03 | 8.14E-04 | 1.00E-03 | 9.59E-04 |
| Isorhamnetin | 9.48E-04 | 4.91E-04 | 1.15E-04 | 1.27E-04 | 4.57E-04 | 5.24E-04 | 7.40E-04 | 6.18E-04 | 4.09E-04 | 8.75E-04 | 1.28E-03 | 3.82E-04 | 7.38E-04 | 4.84E-04 | 6.73E-04 | 4.89E-04 | 7.69E-04 | 8.20E-04 |
| Benzaldehyde | 8.48E-04 | 1.20E-03 | 1.11E-03 | 1.35E-03 | 1.31E-03 | 1.13E-03 | 9.13E-04 | 6.75E-04 | 6.86E-04 | 8.08E-04 | 8.29E-04 | 9.43E-04 | 7.25E-04 | 7.68E-04 | 7.68E-04 | 8.28E-04 | 9.34E-04 | 1.03E-03 |
| Cyclohexanecarboxylic acid, 1,3,5-trihydroxy-4-[[(2E)-3-(4-hydroxy-3-methoxyphenyl)-1-oxo-2-propen-1-yl]oxy]-, (1alpha,3alpha,4alpha,5beta)- | 1.68E-03 | 1.88E-03 | 1.57E-03 | 1.88E-03 | 1.47E-03 | 2.09E-03 | 8.70E-04 | 1.18E-03 | 7.03E-04 | 1.08E-03 | 1.44E-03 | 1.39E-03 | 1.14E-03 | 6.51E-04 | 9.81E-04 | 1.43E-03 | 1.61E-03 | 1.50E-03 |
| 3',5'-Dihydroxyacetophenone | 6.82E-04 | 9.86E-04 | 1.62E-03 | 2.29E-03 | 2.11E-03 | 2.38E-03 | 1.01E-03 | 6.62E-04 | 1.07E-03 | 7.46E-04 | 7.27E-04 | 7.93E-04 | 3.86E-04 | 4.37E-04 | 4.63E-04 | 8.74E-04 | 4.35E-04 | 7.49E-04 |
| Quinic acid | 1.28E-03 | 1.37E-03 | 1.07E-03 | 1.57E-03 | 1.28E-03 | 9.54E-04 | 1.13E-03 | 1.47E-03 | 6.47E-04 | 9.32E-04 | 1.59E-03 | 1.56E-03 | 8.68E-04 | 7.31E-04 | 1.05E-03 | 1.18E-03 | 1.27E-03 | 1.48E-03 |
| Ayapin | 1.86E-03 | 1.85E-03 | 2.01E-03 | 2.21E-03 | 2.20E-03 | 1.90E-03 | 1.20E-03 | 1.61E-03 | 1.12E-03 | 1.39E-03 | 1.48E-03 | 1.49E-03 | 1.33E-03 | 1.07E-03 | 1.19E-03 | 1.57E-03 | 1.44E-03 | 1.44E-03 |
| 5,7alpha-Dihydro-1,4,4,7a-tetramethyl-4H-indene | 1.21E-03 | 1.29E-03 | 1.45E-03 | 1.83E-03 | 1.72E-03 | 1.87E-03 | 1.49E-03 | 1.21E-03 | 1.41E-03 | 1.47E-03 | 1.65E-03 | 1.65E-03 | 1.38E-03 | 1.36E-03 | 1.18E-03 | 1.62E-03 | 1.72E-03 | 1.62E-03 |
| (2E)-3-(4-hydroxy-3-methoxyphenyl)prop-2-enal | 6.67E-04 | 1.02E-03 | 1.29E-03 | 1.55E-03 | 1.66E-03 | 1.79E-03 | 1.29E-03 | 1.03E-03 | 1.38E-03 | 2.36E-03 | 8.17E-04 | 1.72E-03 | 2.58E-03 | 1.81E-03 | 2.21E-03 | 1.45E-03 | 4.97E-04 | 1.55E-03 |
| 2-(6-hydroxy-6-methyloctyl)-2H-furan-5-one | 1.25E-03 | 1.45E-03 | 1.60E-03 | 2.13E-03 | 1.88E-03 | 2.04E-03 | 1.62E-03 | 1.41E-03 | 1.49E-03 | 1.49E-03 | 1.76E-03 | 1.78E-03 | 1.51E-03 | 1.38E-03 | 1.30E-03 | 1.73E-03 | 1.77E-03 | 1.73E-03 |
| trans-Melilotoside | 9.57E-04 | 1.57E-03 | 1.85E-03 | 1.98E-03 | 2.03E-03 | 2.59E-03 | 1.72E-03 | 1.43E-03 | 1.12E-03 | 1.79E-03 | 1.77E-03 | 1.67E-03 | 1.20E-03 | 1.01E-03 | 1.52E-03 | 1.84E-03 | 1.15E-03 | 1.57E-03 |
| Scopoletin | 1.08E-03 | 1.29E-03 | 1.48E-03 | 2.31E-03 | 1.47E-03 | 1.17E-03 | 1.73E-03 | 1.17E-03 | 1.65E-03 | 1.84E-03 | 2.51E-03 | 1.87E-03 | 1.74E-03 | 1.67E-03 | 1.38E-03 | 1.28E-03 | 1.77E-03 | 6.53E-04 |
| Kojibiose | 1.01E-03 | 1.31E-03 | 1.54E-03 | 1.98E-03 | 1.40E-03 | 1.46E-03 | 1.47E-03 | 9.66E-04 | 1.20E-03 | 1.58E-03 | 1.35E-03 | 2.01E-03 | 1.57E-03 | 1.04E-03 | 1.40E-03 | 1.25E-03 | 1.78E-03 | 1.22E-03 |
| 7-[(2S,3R,4S,5S,6R)-3,4,5-trihydroxy-6-(hydroxymethyl)oxan-2-yl]oxychromen-2-one | 9.80E-04 | 1.19E-03 | 1.34E-03 | 1.46E-03 | 1.64E-03 | 1.25E-03 | 1.96E-03 | 1.95E-03 | 2.56E-03 | 1.87E-03 | 1.26E-03 | 1.33E-14 | 1.43E-03 | 1.95E-03 | 1.34E-03 | 1.51E-03 | 2.59E-03 | 1.44E-03 |
| Dihydroroseoside | 4.87E-04 | 7.68E-04 | 1.91E-03 | 2.23E-03 | 2.06E-03 | 2.32E-03 | 1.68E-03 | 6.80E-04 | 1.40E-03 | 1.22E-03 | 1.76E-03 | 1.63E-03 | 7.65E-04 | 1.00E-03 | 7.68E-04 | 1.81E-03 | 7.56E-04 | 9.09E-04 |
| Tributylamine | 1.34E-03 | 1.51E-03 | 1.76E-03 | 2.24E-03 | 2.20E-03 | 2.01E-03 | 1.63E-03 | 1.58E-03 | 1.52E-03 | 1.53E-03 | 1.84E-03 | 2.09E-03 | 1.45E-03 | 1.47E-03 | 1.44E-03 | 1.87E-03 | 1.96E-03 | 1.85E-03 |
| NIPECOTIC ACID | 1.69E-03 | 1.71E-03 | 1.40E-03 | 2.18E-03 | 2.45E-03 | 1.39E-14 | 9.89E-04 | 2.01E-03 | 1.01E-03 | 1.46E-03 | 1.49E-03 | 1.92E-03 | 7.91E-04 | 1.25E-03 | 1.99E-03 | 2.85E-03 | 3.00E-03 | 1.94E-03 |
| D-Tagatose | 1.18E-03 | 1.49E-03 | 1.82E-03 | 2.78E-03 | 2.41E-03 | 2.18E-03 | 1.77E-03 | 1.40E-03 | 1.56E-03 | 1.69E-03 | 1.76E-03 | 1.93E-03 | 1.77E-03 | 1.60E-03 | 1.07E-03 | 1.92E-03 | 1.80E-03 | 2.13E-03 |
| 6-Methylcoumarin | 1.46E-03 | 1.86E-03 | 2.14E-03 | 1.13E-03 | 1.33E-03 | 1.19E-03 | 9.75E-04 | 9.76E-04 | 1.31E-03 | 1.69E-03 | 1.28E-03 | 1.90E-03 | 8.70E-04 | 1.16E-03 | 1.16E-03 | 1.65E-03 | 1.32E-03 | 2.55E-03 |
| Caffeic acid | 1.75E-03 | 2.05E-03 | 1.70E-03 | 1.98E-03 | 2.08E-03 | 1.65E-03 | 1.38E-03 | 2.27E-03 | 1.05E-03 | 1.20E-03 | 1.22E-03 | 1.22E-03 | 1.15E-03 | 1.19E-03 | 2.05E-03 | 1.30E-03 | 1.54E-03 | 1.52E-03 |
| 1H-Indene-3-carboxylic acid, 3a,4,5,6,7,7a-hexahydro-3a,7,7-trimethyl- | 7.56E-04 | 9.64E-04 | 2.34E-03 | 3.23E-03 | 2.91E-03 | 3.97E-03 | 2.13E-03 | 1.05E-03 | 2.29E-03 | 1.32E-03 | 2.00E-03 | 2.01E-03 | 1.02E-03 | 1.34E-03 | 1.09E-03 | 2.12E-03 | 1.02E-03 | 1.11E-03 |
| Phenylethylamine | 3.88E-03 | 1.98E-03 | 6.03E-03 | 1.31E-03 | 1.68E-04 | 8.68E-04 | 1.36E-04 | 1.04E-04 | 1.30E-03 | 3.75E-04 | 5.44E-03 | 7.02E-04 | 6.51E-04 | 2.73E-05 | 1.62E-05 | 2.18E-03 | 8.13E-04 | 3.14E-04 |
| 3-Methyl-1-(2,4,6-trihydroxyphenyl)-1-butanone | 1.63E-03 | 1.87E-03 | 2.02E-03 | 2.52E-03 | 2.44E-03 | 2.59E-03 | 2.03E-03 | 1.77E-03 | 1.96E-03 | 1.93E-03 | 2.25E-03 | 2.28E-03 | 1.87E-03 | 1.00E-14 | 1.70E-03 | 2.30E-03 | 2.40E-03 | 2.22E-03 |
| beta-Lactose | 7.96E-04 | 2.21E-03 | 2.23E-03 | 1.81E-03 | 1.82E-03 | 2.60E-03 | 1.92E-03 | 3.39E-03 | 1.70E-03 | 2.41E-03 | 1.01E-03 | 1.27E-03 | 1.85E-03 | 2.86E-03 | 2.31E-03 | 1.47E-03 | 2.52E-03 | 2.75E-03 |
| 6-Hydroxy-4-methylcoumarin, 6-Hydroxy-4-methyl-2-benzopyrone | 1.43E-03 | 1.60E-03 | 1.82E-03 | 2.03E-03 | 1.79E-03 | 1.41E-03 | 2.03E-03 | 1.43E-03 | 1.94E-03 | 1.76E-03 | 2.90E-03 | 2.26E-03 | 1.94E-03 | 1.94E-03 | 1.66E-03 | 1.82E-03 | 2.41E-03 | 2.22E-03 |
| 1-deoxy-1-(N6-lysino)-D-fructose | 1.27E-03 | 1.81E-03 | 1.84E-03 | 1.80E-03 | 7.17E-04 | 1.02E-03 | 1.97E-03 | 2.26E-03 | 2.05E-03 | 3.06E-03 | 3.52E-03 | 2.36E-03 | 1.64E-03 | 1.45E-03 | 1.57E-03 | 2.40E-03 | 2.32E-03 | 2.46E-03 |
| Palmitic amide | 7.21E-04 | 8.96E-04 | 1.09E-03 | 1.39E-03 | 1.24E-03 | 1.35E-03 | 9.84E-04 | 8.50E-04 | 1.03E-03 | 9.33E-04 | 1.12E-03 | 1.05E-03 | 9.27E-04 | 1.20E-03 | 8.16E-04 | 1.03E-03 | 1.19E-03 | 1.16E-03 |
| 1,2,3-Trihydroxybenzene | 1.52E-03 | 1.90E-03 | 2.21E-03 | 2.66E-03 | 2.72E-03 | 2.30E-03 | 2.30E-03 | 1.50E-03 | 1.98E-03 | 1.89E-03 | 2.49E-03 | 2.61E-03 | 1.85E-03 | 1.92E-03 | 1.61E-03 | 2.00E-03 | 2.27E-03 | 2.00E-03 |
| 2',4'-Dihydroxy-2-biphenylcarboxylic acid | 1.64E-03 | 2.19E-03 | 2.24E-03 | 2.28E-03 | 3.10E-03 | 2.77E-03 | 3.51E-03 | 2.93E-03 | 2.90E-03 | 2.90E-03 | 1.55E-03 | 2.85E-03 | 2.63E-03 | 2.64E-03 | 2.17E-03 | 2.33E-03 | 2.36E-03 | 1.52E-03 |
| Coumarin | 1.34E-03 | 1.40E-03 | 4.20E-03 | 2.68E-03 | 2.48E-03 | 4.08E-03 | 2.80E-03 | 1.96E-03 | 6.46E-03 | 3.47E-03 | 6.07E-03 | 7.02E-03 | 5.68E-03 | 2.97E-03 | 3.81E-03 | 2.39E-03 | 1.28E-03 | 1.59E-03 |
| 9,12,15-Octadecatrienoic acid, 3-(hexopyranosyloxy)-2-hydroxypropyl ester, (9Z,12Z,15Z)- | 1.57E-03 | 1.21E-03 | 7.11E-04 | 1.77E-03 | 1.43E-03 | 3.31E-03 | 2.93E-03 | 1.92E-04 | 1.70E-03 | 5.10E-03 | 2.13E-03 | 3.05E-03 | 2.60E-03 | 1.41E-03 | 1.97E-03 | 8.73E-04 | 1.30E-03 | 7.38E-04 |
| Domoic acid | 1.91E-03 | 2.33E-03 | 2.81E-03 | 3.40E-03 | 2.97E-03 | 3.07E-03 | 2.66E-03 | 2.23E-03 | 2.44E-03 | 2.62E-03 | 3.12E-03 | 3.18E-03 | 2.49E-03 | 2.16E-03 | 2.13E-03 | 3.04E-03 | 1.26E-14 | 2.98E-03 |
| Afzelin | 2.98E-03 | 2.78E-03 | 3.03E-03 | 2.98E-03 | 2.85E-03 | 2.00E-03 | 2.89E-03 | 2.91E-03 | 3.09E-03 | 3.72E-03 | 2.55E-03 | 2.18E-03 | 2.64E-03 | 2.79E-03 | 3.01E-03 | 2.66E-03 | 3.40E-03 | 2.98E-03 |
| Confertifoline | 2.01E-03 | 2.38E-03 | 2.80E-03 | 3.56E-03 | 3.00E-03 | 3.54E-03 | 2.74E-03 | 2.22E-03 | 2.37E-03 | 2.53E-03 | 3.05E-03 | 2.96E-03 | 2.18E-03 | 2.40E-03 | 2.02E-03 | 3.02E-03 | 3.09E-03 | 3.10E-03 |
| Catechin | 5.38E-04 | 8.74E-04 | 2.40E-03 | 1.78E-03 | 4.02E-03 | 4.67E-03 | 3.10E-03 | 7.74E-04 | 2.11E-03 | 6.34E-03 | 9.25E-04 | 1.19E-02 | 6.79E-03 | 4.62E-03 | 2.05E-03 | 3.09E-03 | 2.18E-03 | 2.53E-03 |
| (3E)-4-(2-Carboxyphenyl)-2-oxobut-3-enoate | 4.48E-03 | 4.18E-03 | 2.93E-03 | 2.66E-03 | 1.97E-03 | 2.58E-03 | 3.30E-03 | 5.90E-03 | 2.26E-03 | 5.50E-03 | 2.24E-03 | 4.21E-03 | 2.60E-03 | 1.42E-03 | 3.31E-03 | 1.60E-03 | 2.76E-03 | 2.84E-03 |
| 10-hydroxy-8E-Decene-4,6-diynoic acid | 1.31E-03 | 1.82E-03 | 2.40E-03 | 2.29E-03 | 2.54E-03 | 2.57E-03 | 3.15E-03 | 3.29E-03 | 3.05E-03 | 3.69E-03 | 4.13E-03 | 5.09E-03 | 3.09E-03 | 2.74E-03 | 2.63E-03 | 2.51E-03 | 3.44E-03 | 2.42E-03 |
| beta-D-Glucopyranose, 1-O-[(2E)-3-(2-hydroxyphenyl)-1-oxo-2-propen-1-yl]- | 1.51E-03 | 2.61E-03 | 2.31E-03 | 4.03E-03 | 3.50E-03 | 3.12E-03 | 3.24E-03 | 3.49E-03 | 3.46E-03 | 3.86E-03 | 2.33E-03 | 4.90E-03 | 3.70E-03 | 3.08E-03 | 2.46E-03 | 3.39E-03 | 3.00E-03 | 3.36E-03 |
| 2-Phenylethanol | 2.12E-03 | 2.71E-03 | 3.24E-03 | 3.59E-03 | 3.17E-03 | 2.90E-03 | 2.92E-03 | 2.81E-03 | 3.01E-03 | 2.92E-03 | 3.69E-03 | 3.58E-03 | 3.09E-03 | 2.97E-03 | 2.72E-03 | 2.95E-03 | 3.40E-03 | 2.93E-03 |
| 2-(3,4-dihydroxyphenyl)-3,5,7-trihydroxy-3,4-dihydro-2H-1-benzopyran-4-one | 2.18E-03 | 3.25E-03 | 2.99E-03 | 3.26E-03 | 3.89E-03 | 4.18E-03 | 5.26E-03 | 3.91E-03 | 3.83E-03 | 3.92E-03 | 2.12E-03 | 4.01E-03 | 4.16E-03 | 3.65E-03 | 3.05E-03 | 3.04E-03 | 3.51E-03 | 2.10E-03 |
| 4-Methylbenzaldehyde | 3.68E-03 | 2.79E-03 | 4.08E-03 | 2.88E-03 | 7.50E-04 | 2.41E-03 | 5.26E-04 | 1.45E-04 | 4.40E-03 | 1.50E-03 | 4.10E-03 | 2.53E-03 | 1.94E-03 | 1.71E-04 | 1.13E-04 | 5.71E-03 | 7.28E-04 | 1.26E-03 |
| 2-Oxo-5-methylthiopentanoic acid | 1.08E-03 | 2.97E-03 | 4.31E-03 | 3.37E-03 | 3.21E-03 | 4.15E-03 | 3.36E-03 | 5.02E-03 | 2.72E-03 | 4.36E-03 | 1.93E-03 | 2.21E-03 | 3.53E-03 | 4.61E-03 | 3.85E-03 | 2.76E-03 | 4.31E-03 | 5.02E-03 |
| N-Acetylornithine | 2.23E-02 | 7.09E-04 | 1.66E-03 | 3.80E-03 | 2.85E-04 | 4.05E-04 | 3.35E-04 | 1.59E-03 | 3.72E-04 | 1.06E-03 | 9.89E-03 | 7.90E-04 | 7.11E-04 | 2.46E-03 | 2.41E-03 | 9.87E-04 | 1.08E-02 | 2.52E-03 |
| [(2R,3S,4S,5R,6R)-6-[(5,8-dihydroxy-4-oxo-2,3-dihydro-1H-naphthalen-1-yl)oxy]-3,4,5-trihydroxyoxan-2-yl]methyl 3,4,5-trihydroxybenzoate | 8.02E-03 | 7.98E-03 | 5.43E-03 | 2.82E-03 | 3.14E-03 | 1.74E-03 | 2.41E-03 | 4.03E-03 | 3.90E-03 | 5.33E-03 | 5.08E-03 | 4.47E-03 | 3.36E-03 | 3.85E-03 | 3.86E-03 | 6.01E-03 | 4.69E-03 | 8.19E-03 |
| 3,5-Dihydroxy-1,4-naphthoquinone | 4.15E-03 | 3.99E-03 | 3.44E-03 | 8.67E-03 | 9.95E-03 | 5.12E-03 | 1.10E-02 | 4.89E-03 | 1.54E-02 | 1.20E-02 | 2.62E-03 | 1.24E-02 | 1.15E-02 | 5.24E-03 | 1.09E-02 | 8.20E-03 | 3.30E-03 | 1.10E-02 |
| Adenine | 7.19E-03 | 6.25E-03 | 6.27E-03 | 5.23E-03 | 3.24E-03 | 3.94E-03 | 2.65E-03 | 4.79E-03 | 2.04E-03 | 5.40E-03 | 7.10E-03 | 3.74E-03 | 2.63E-03 | 1.78E-03 | 2.14E-03 | 5.77E-03 | 4.36E-03 | 4.61E-03 |
| p-Coumaric acid | 1.91E-03 | 4.11E-03 | 5.34E-03 | 6.53E-03 | 7.35E-03 | 8.01E-03 | 6.62E-03 | 5.76E-03 | 5.66E-03 | 7.84E-03 | 3.20E-03 | 5.77E-03 | 6.92E-03 | 5.65E-03 | 8.66E-03 | 7.36E-03 | 3.02E-03 | 8.05E-03 |
| (卤)-Taxifolin | 3.53E-03 | 5.15E-03 | 4.35E-03 | 4.94E-03 | 6.21E-03 | 5.79E-03 | 9.58E-03 | 8.12E-03 | 7.49E-03 | 8.13E-03 | 5.31E-03 | 6.68E-03 | 6.64E-03 | 6.91E-03 | 6.20E-03 | 7.68E-03 | 5.68E-03 | 5.78E-03 |
| 4',5,7-trihydroxy-3,6-dimethoxyflavone | 5.77E-03 | 3.48E-03 | 3.73E-03 | 6.26E-03 | 4.09E-03 | 7.17E-03 | 7.13E-03 | 4.73E-03 | 5.40E-03 | 1.07E-02 | 7.30E-03 | 3.39E-03 | 1.55E-02 | 5.40E-03 | 6.52E-03 | 6.62E-03 | 7.29E-03 | 1.71E-02 |
| Citrulline | 1.83E-02 | 7.65E-03 | 2.27E-03 | 4.30E-03 | 1.20E-03 | 7.69E-04 | 9.15E-04 | 7.56E-03 | 9.34E-04 | 5.10E-03 | 1.15E-02 | 1.13E-03 | 1.93E-03 | 9.44E-04 | 5.40E-03 | 2.52E-03 | 7.35E-03 | 9.97E-03 |
| Fisetin | 2.92E-03 | 4.59E-03 | 1.71E-02 | 5.70E-03 | 6.56E-03 | 6.27E-03 | 6.87E-03 | 6.78E-03 | 7.05E-03 | 7.52E-03 | 6.51E-03 | 1.73E-02 | 5.13E-03 | 7.17E-03 | 1.81E-02 | 7.32E-03 | 8.96E-03 | 8.75E-03 |
| Tris(1-chloro-2-propyl)phosphate | 5.90E-03 | 6.59E-03 | 8.51E-03 | 9.48E-03 | 8.44E-03 | 9.54E-03 | 7.13E-03 | 6.23E-03 | 6.71E-03 | 6.70E-03 | 9.11E-03 | 8.34E-03 | 6.89E-03 | 6.48E-03 | 5.87E-03 | 8.40E-03 | 8.05E-03 | 8.57E-03 |
| Mahaleboside | 6.16E-03 | 9.73E-03 | 6.81E-03 | 8.39E-03 | 9.96E-03 | 1.08E-02 | 1.29E-02 | 1.70E-02 | 1.20E-02 | 1.64E-02 | 1.09E-02 | 1.20E-02 | 9.80E-03 | 7.84E-03 | 8.24E-03 | 7.68E-03 | 1.58E-02 | 1.10E-02 |
| (2Z)-4,6-dihydroxy-2-[(3,4,5-trihydroxyphenyl)methylidene]-1-benzofuran-3-one | 5.39E-03 | 7.15E-03 | 8.83E-03 | 9.79E-03 | 9.97E-03 | 8.10E-03 | 1.21E-02 | 6.60E-03 | 9.52E-03 | 1.08E-02 | 4.29E-03 | 9.91E-03 | 6.86E-03 | 6.67E-03 | 7.81E-03 | 8.78E-03 | 6.04E-03 | 1.06E-02 |
| (R)-(-)-Mellein | 8.29E-03 | 1.11E-02 | 1.36E-02 | 1.14E-02 | 1.28E-02 | 1.04E-02 | 1.06E-02 | 1.16E-02 | 1.18E-02 | 1.24E-02 | 1.47E-02 | 1.39E-02 | 1.08E-02 | 1.18E-02 | 9.61E-03 | 1.21E-02 | 1.14E-02 | 1.12E-02 |
| 1H-Benzotriazole-4-carboxylic acid (Tentative) | 1.18E-02 | 1.63E-02 | 1.45E-02 | 1.71E-02 | 1.63E-02 | 1.37E-02 | 9.57E-03 | 8.26E-03 | 8.17E-03 | 9.71E-03 | 1.00E-02 | 1.21E-02 | 8.84E-03 | 9.28E-03 | 9.65E-03 | 1.12E-02 | 1.15E-02 | 1.21E-02 |
| Myricetin-3-Xyloside | 1.76E-02 | 1.28E-02 | 1.29E-02 | 9.81E-03 | 7.44E-03 | 8.95E-03 | 8.32E-03 | 1.38E-02 | 9.22E-03 | 7.93E-03 | 1.01E-02 | 1.09E-02 | 1.10E-02 | 1.49E-02 | 1.40E-02 | 1.62E-02 | 1.49E-02 | 9.52E-03 |
| Tri(butoxyethyl)phosphate | 9.71E-03 | 1.12E-02 | 1.62E-02 | 1.54E-02 | 1.37E-02 | 1.53E-02 | 1.37E-02 | 1.13E-02 | 1.20E-02 | 1.42E-02 | 1.81E-02 | 1.46E-02 | 1.13E-02 | 1.07E-02 | 9.82E-03 | 1.56E-02 | 1.48E-02 | 1.55E-02 |
| Myricetin 3-galactoside | 1.39E-02 | 1.36E-02 | 1.32E-02 | 1.10E-02 | 1.27E-02 | 1.12E-02 | 1.42E-02 | 1.34E-02 | 1.64E-02 | 1.05E-02 | 9.74E-03 | 1.08E-02 | 1.45E-02 | 1.72E-02 | 1.52E-02 | 1.45E-02 | 1.20E-02 | 1.21E-02 |
| p-Octopamine | 1.86E-02 | 1.79E-02 | 3.85E-02 | 5.64E-03 | 7.83E-04 | 1.51E-03 | 2.86E-04 | 1.28E-03 | 1.88E-03 | 1.61E-03 | 5.11E-02 | 5.11E-03 | 8.99E-04 | 2.46E-04 | 1.99E-05 | 6.77E-03 | 7.29E-03 | 1.09E-03 |
| Threonic acid | 9.92E-03 | 1.05E-02 | 1.10E-02 | 2.13E-02 | 1.63E-02 | 1.63E-02 | 1.44E-02 | 1.21E-02 | 1.23E-02 | 1.27E-02 | 1.04E-02 | 1.13E-02 | 9.26E-03 | 1.07E-02 | 1.05E-02 | 1.32E-02 | 1.01E-02 | 1.51E-02 |
| Quercitrin | 1.28E-01 | 1.06E-01 | 3.12E-02 | 3.13E-03 | 1.36E-03 | 6.91E-04 | 1.90E-02 | 3.07E-02 | 4.92E-03 | 8.87E-04 | 9.38E-04 | 1.64E-03 | 7.16E-02 | 2.66E-02 | 3.49E-02 | 7.04E-04 | 6.71E-03 | 7.35E-03 |
| 3-O-p-Coumaroylquinic acid | 2.78E-02 | 2.96E-02 | 1.81E-02 | 2.59E-02 | 1.53E-02 | 1.17E-02 | 1.36E-02 | 4.02E-02 | 1.15E-02 | 1.71E-02 | 1.65E-02 | 1.52E-02 | 1.64E-02 | 1.39E-02 | 3.46E-02 | 1.71E-02 | 1.98E-02 | 1.86E-02 |
| Trigonelline | 2.18E-02 | 2.40E-02 | 2.31E-02 | 2.11E-02 | 2.03E-02 | 3.22E-02 | 1.62E-02 | 2.24E-02 | 1.45E-02 | 2.12E-02 | 2.39E-02 | 1.49E-02 | 1.76E-02 | 1.50E-02 | 1.53E-02 | 1.50E-02 | 1.62E-02 | 1.62E-02 |
| Myricetin | 2.12E-02 | 2.11E-02 | 2.01E-02 | 1.58E-02 | 2.12E-02 | 1.85E-02 | 2.17E-02 | 2.36E-02 | 2.41E-02 | 1.80E-02 | 1.60E-02 | 1.72E-02 | 2.44E-02 | 2.93E-02 | 2.53E-02 | 2.93E-02 | 1.78E-02 | 1.68E-02 |
| Atropaldehyde | 1.41E-02 | 2.01E-02 | 2.54E-02 | 2.21E-02 | 2.28E-02 | 1.78E-02 | 2.09E-02 | 1.87E-02 | 2.30E-02 | 2.16E-02 | 2.49E-02 | 2.67E-02 | 2.14E-02 | 1.96E-02 | 1.85E-02 | 2.14E-02 | 2.21E-02 | 2.04E-02 |
| Kaempferol-3-O-alpha-L-arabinoside | 1.24E-02 | 1.72E-02 | 2.40E-02 | 2.63E-02 | 3.21E-02 | 3.44E-02 | 2.42E-02 | 1.73E-02 | 2.46E-02 | 2.31E-02 | 2.89E-02 | 2.15E-02 | 1.99E-02 | 2.85E-02 | 2.50E-02 | 2.68E-02 | 2.83E-02 | 2.94E-02 |
| 5-hydroxy-2-(4-hydroxyphenyl)-7-methoxy-2,3-dihydrochromen-4-one | 1.48E-02 | 1.96E-02 | 2.07E-02 | 2.41E-02 | 2.73E-02 | 2.26E-02 | 2.54E-02 | 2.36E-02 | 2.18E-02 | 2.53E-02 | 2.08E-02 | 2.19E-02 | 1.89E-02 | 2.29E-02 | 2.80E-02 | 2.17E-02 | 2.96E-02 | 2.80E-02 |
| 8Z-Decene-4,6-diynoic acid | 1.66E-02 | 2.32E-02 | 2.48E-02 | 2.49E-02 | 2.24E-02 | 2.69E-02 | 2.30E-02 | 3.12E-02 | 2.52E-02 | 2.69E-02 | 1.97E-02 | 2.05E-02 | 2.08E-02 | 2.55E-02 | 2.12E-02 | 1.78E-02 | 3.79E-02 | 2.13E-02 |
| L-Glutamic acid | 1.54E-02 | 1.94E-02 | 2.05E-02 | 3.23E-02 | 2.68E-02 | 2.88E-02 | 2.11E-02 | 2.46E-02 | 2.54E-02 | 2.32E-02 | 3.04E-02 | 1.61E-02 | 1.50E-02 | 1.69E-02 | 1.98E-02 | 2.65E-02 | 2.22E-02 | 2.65E-02 |
| Isogentisin | 1.70E-02 | 2.36E-02 | 2.15E-02 | 2.90E-02 | 3.27E-02 | 2.81E-02 | 4.02E-02 | 2.95E-02 | 3.06E-02 | 3.14E-02 | 1.71E-02 | 2.93E-02 | 2.92E-02 | 2.91E-02 | 2.47E-02 | 2.71E-02 | 2.64E-02 | 1.66E-02 |
| Astragalin | 1.38E-02 | 2.29E-02 | 2.22E-02 | 2.75E-02 | 2.95E-02 | 2.71E-02 | 3.37E-02 | 2.20E-02 | 2.90E-02 | 2.99E-02 | 2.61E-02 | 3.11E-02 | 2.72E-02 | 2.85E-02 | 2.66E-02 | 2.54E-02 | 3.02E-02 | 3.07E-02 |
| Kaempferol-3-O-arabinopyranoside | 1.57E-02 | 2.61E-02 | 2.88E-02 | 4.66E-02 | 4.37E-02 | 4.44E-02 | 2.97E-02 | 2.57E-02 | 3.04E-02 | 3.38E-02 | 3.73E-02 | 2.97E-02 | 2.68E-02 | 3.18E-02 | 3.66E-02 | 4.23E-02 | 4.61E-02 | 4.59E-02 |
| myricetin-3-O-pentoside | 7.23E-02 | 4.30E-02 | 4.17E-02 | 3.23E-02 | 2.66E-02 | 2.95E-02 | 2.70E-02 | 4.57E-02 | 3.17E-02 | 2.43E-02 | 3.28E-02 | 3.19E-02 | 4.09E-02 | 4.59E-02 | 4.31E-02 | 5.00E-02 | 3.69E-02 | 3.55E-02 |
| Phthalic anhydride | 2.60E-02 | 3.50E-02 | 4.23E-02 | 4.35E-02 | 3.80E-02 | 4.39E-02 | 3.46E-02 | 3.00E-02 | 3.26E-02 | 3.24E-02 | 4.48E-02 | 3.91E-02 | 3.12E-02 | 2.99E-02 | 2.60E-02 | 4.31E-02 | 3.94E-02 | 4.40E-02 |
| 2-Naphthol | 2.24E-02 | 3.13E-02 | 4.32E-02 | 3.37E-02 | 4.96E-02 | 3.52E-02 | 4.76E-02 | 4.55E-02 | 4.97E-02 | 6.82E-02 | 5.91E-02 | 8.24E-02 | 4.99E-02 | 4.16E-02 | 4.21E-02 | 4.14E-02 | 5.36E-02 | 3.66E-02 |
| Kaempferol 3-alpha-D-glucoside | 2.93E-02 | 3.77E-02 | 3.74E-02 | 5.13E-02 | 5.29E-02 | 4.67E-02 | 5.11E-02 | 5.19E-02 | 5.24E-02 | 6.26E-02 | 6.05E-02 | 5.28E-02 | 4.09E-02 | 5.40E-02 | 5.63E-02 | 5.71E-02 | 6.40E-02 | 6.96E-02 |
| Isoquercitrin | 5.48E-02 | 4.77E-02 | 5.36E-02 | 7.43E-02 | 7.89E-02 | 8.23E-02 | 7.44E-02 | 5.33E-02 | 8.05E-02 | 6.78E-02 | 4.93E-02 | 7.37E-02 | 6.22E-02 | 6.12E-02 | 5.09E-02 | 5.25E-02 | 5.29E-02 | 6.04E-02 |
| Myricetin 3-glucoside | 1.75E-01 | 1.61E-01 | 1.61E-01 | 1.34E-01 | 1.54E-01 | 1.57E-01 | 1.70E-01 | 1.84E-01 | 1.94E-01 | 1.31E-01 | 1.24E-01 | 1.41E-01 | 1.73E-01 | 2.20E-01 | 1.86E-01 | 1.77E-01 | 1.44E-01 | 1.50E-01 |
| p-Xylene | 8.81E-15 | 1.02E-14 | 5.98E-06 | 1.44E-14 | 1.32E-14 | 1.52E-05 | 1.17E-14 | 9.69E-15 | 1.06E-14 | 1.57E-05 | 1.31E-14 | 2.03E-05 | 1.07E-14 | 1.00E-14 | 9.43E-15 | 1.30E-14 | 1.26E-14 | 1.30E-14 |
| m-Coumaric acid | 8.81E-15 | 1.02E-14 | 5.10E-05 | 1.44E-14 | 5.11E-05 | 5.63E-05 | 1.17E-14 | 9.69E-15 | 4.07E-05 | 8.71E-05 | 4.74E-05 | 1.33E-14 | 1.07E-14 | 1.00E-14 | 9.43E-15 | 8.67E-05 | 6.31E-05 | 1.34E-04 |
| 2-amino-4'-hydroxy-Propiophenone | 3.95E-04 | 3.07E-04 | 5.59E-04 | 3.49E-04 | 5.71E-05 | 7.36E-05 | 1.17E-14 | 5.76E-05 | 1.85E-04 | 6.75E-05 | 4.65E-04 | 3.75E-04 | 6.29E-05 | 2.04E-05 | 4.32E-06 | 1.87E-04 | 2.81E-04 | 3.06E-05 |
| Coumaric acid O-glucoside | 6.83E-06 | 1.02E-14 | 1.19E-14 | 2.57E-03 | 1.32E-14 | 1.39E-14 | 1.17E-14 | 9.69E-15 | 1.06E-14 | 8.86E-06 | 1.31E-14 | 1.33E-14 | 1.07E-14 | 1.00E-14 | 9.43E-15 | 1.30E-14 | 1.26E-14 | 1.30E-14 |
| beta-D-Glucopyranoside, 4,8-dihydroxy-1-naphthalenyl | 6.33E-04 | 8.22E-04 | 7.06E-04 | 1.44E-14 | 1.32E-14 | 1.39E-14 | 1.17E-14 | 9.69E-15 | 1.06E-14 | 1.12E-14 | 5.46E-04 | 6.20E-04 | 1.07E-14 | 1.00E-14 | 4.96E-04 | 5.17E-04 | 1.26E-14 | 6.19E-04 |
| 9,11-methane-epoxy PGF1伪 | 3.44E-03 | 1.46E-03 | 7.88E-04 | 2.43E-03 | 2.90E-03 | 6.66E-03 | 5.95E-03 | 9.69E-15 | 3.97E-03 | 1.06E-02 | 3.21E-03 | 1.33E-14 | 5.14E-03 | 2.79E-03 | 5.34E-03 | 1.30E-14 | 1.86E-03 | 1.20E-03 |
| Spirost-5-en-3-ol, (3beta,25R)- | 1.32E-05 | 3.60E-06 | 1.08E-05 | 1.34E-05 | 8.24E-06 | 7.85E-06 | 1.17E-14 | 9.65E-06 | 2.65E-05 | 1.12E-14 | 1.31E-14 | 1.87E-05 | 1.07E-14 | 8.53E-06 | 3.66E-06 | 8.51E-06 | 1.26E-14 | 6.34E-06 |
| gamma-CEHC Glc | 5.37E-06 | 4.28E-05 | 2.92E-05 | 6.07E-05 | 6.69E-05 | 5.32E-05 | 5.58E-05 | 2.34E-05 | 3.57E-05 | 2.91E-05 | 2.70E-05 | 2.55E-05 | 1.90E-05 | 6.59E-06 | 2.01E-05 | 4.48E-05 | 2.49E-05 | 1.65E-05 |
| Cyanidin 3-O-arabinoside | 4.09E-04 | 4.01E-04 | 3.86E-04 | 6.85E-05 | 1.48E-05 | 7.64E-05 | 3.76E-04 | 3.27E-04 | 3.76E-04 | 6.85E-05 | 1.48E-05 | 7.64E-05 | 3.17E-04 | 3.02E-04 | 3.17E-04 | 6.85E-05 | 1.48E-05 | 7.64E-05 |
| Peonidin 3-O-galactoside | 7.47E-05 | 8.22E-05 | 8.51E-05 | 2.94E-05 | 1.84E-06 | 1.08E-05 | 1.47E-04 | 1.33E-04 | 1.42E-04 | 2.94E-05 | 1.84E-06 | 1.08E-05 | 2.46E-05 | 2.29E-05 | 2.46E-05 | 2.94E-05 | 1.84E-06 | 1.08E-05 |
| Procyanidin B1 | 1.54E-04 | 1.77E-04 | 1.78E-04 | 6.11E-05 | 4.99E-04 | 1.65E-04 | 6.85E-05 | 6.62E-05 | 6.51E-05 | 6.11E-05 | 4.99E-04 | 1.65E-04 | 7.01E-04 | 9.51E-04 | 6.66E-04 | 6.11E-05 | 4.99E-04 | 1.65E-04 |
| Procyanidin B2 | 1.11E-04 | 1.20E-04 | 1.18E-04 | 7.92E-05 | 8.35E-05 | 9.02E-05 | 7.53E-05 | 7.58E-05 | 7.47E-05 | 7.92E-05 | 8.35E-05 | 9.02E-05 | 1.00E-04 | 1.27E-04 | 1.00E-04 | 7.92E-05 | 8.35E-05 | 9.02E-05 |
| Procyanidin C1 | 3.50E-05 | 3.78E-05 | 4.04E-05 | 1.43E-05 | 8.55E-06 | 1.77E-05 | 1.94E-05 | 1.88E-05 | 2.00E-05 | 1.43E-05 | 8.55E-06 | 1.77E-05 | 4.44E-05 | 6.82E-05 | 4.11E-05 | 1.43E-05 | 8.55E-06 | 1.77E-05 |
| Malvidin 3-O-(6-O-malonyl-beta-D-glucoside) | 3.36E-06 | 2.75E-06 | 1.91E-06 | 8.64E-07 | 1.37E-06 | 4.65E-07 | 2.24E-06 | 1.77E-06 | 1.45E-06 | 8.64E-07 | 1.37E-06 | 4.65E-07 | 1.46E-06 | 1.20E-06 | 1.46E-06 | 8.64E-07 | 1.37E-06 | 4.65E-07 |
| Pelargonidin 3-O-glucoside | 3.78E-06 | 3.78E-06 | 3.96E-06 | 0.00E+00 | 8.67E-08 | 0.00E+00 | 8.55E-06 | 8.11E-06 | 8.68E-06 | 0.00E+00 | 8.67E-08 | 0.00E+00 | 1.30E-06 | 1.14E-06 | 1.30E-06 | 0.00E+00 | 8.67E-08 | 0.00E+00 |
| Procyanidin B3 | 2.66E-04 | 2.87E-04 | 2.82E-04 | 1.29E-04 | 3.43E-04 | 2.24E-04 | 1.30E-04 | 1.23E-04 | 1.27E-04 | 1.29E-04 | 3.43E-04 | 2.24E-04 | 5.70E-04 | 7.87E-04 | 5.70E-04 | 1.29E-04 | 3.43E-04 | 2.24E-04 |
| Cyanidin 3-O-galactoside | 8.40E-03 | 8.44E-03 | 8.56E-03 | 7.22E-04 | 1.49E-04 | 9.70E-04 | 1.37E-02 | 1.27E-02 | 1.30E-02 | 7.22E-04 | 1.49E-04 | 9.70E-04 | 4.13E-03 | 3.86E-03 | 4.13E-03 | 7.22E-04 | 1.49E-04 | 9.70E-04 |
| Delphinidin 3-O-galactoside | 9.34E-03 | 9.21E-03 | 8.98E-03 | 2.11E-04 | 3.51E-05 | 6.05E-04 | 2.17E-02 | 2.03E-02 | 2.23E-02 | 2.11E-04 | 3.51E-05 | 6.05E-04 | 5.23E-03 | 4.65E-03 | 5.23E-03 | 2.11E-04 | 3.51E-05 | 6.05E-04 |
| Peonidin 3-O-glucoside | 4.43E-06 | 4.95E-06 | 4.98E-06 | 6.79E-07 | 2.16E-07 | 1.52E-07 | 5.88E-06 | 5.41E-06 | 6.24E-06 | 6.79E-07 | 2.16E-07 | 1.52E-07 | 1.29E-06 | 1.15E-06 | 1.29E-06 | 6.79E-07 | 2.16E-07 | 1.52E-07 |
| Delphinidin 3-O-arabinoside | 1.41E-04 | 1.57E-04 | 1.63E-04 | 2.28E-06 | 6.12E-07 | 4.47E-06 | 4.36E-04 | 4.40E-04 | 4.45E-04 | 2.28E-06 | 6.12E-07 | 4.47E-06 | 6.16E-05 | 5.54E-05 | 6.16E-05 | 2.28E-06 | 6.12E-07 | 4.47E-06 |
| Malvidin 3-O-glucoside | 1.80E-06 | 1.52E-06 | 1.97E-06 | 8.19E-08 | 1.20E-06 | 2.73E-07 | 9.24E-07 | 1.59E-06 | 1.48E-06 | 8.19E-08 | 1.20E-06 | 2.73E-07 | 4.61E-07 | 4.84E-07 | 4.61E-07 | 8.19E-08 | 1.20E-06 | 2.73E-07 |
| Cyanidin 3-O-glucoside | 7.39E-04 | 7.47E-04 | 8.13E-04 | 1.06E-05 | 2.76E-06 | 1.26E-05 | 1.22E-03 | 1.10E-03 | 1.31E-03 | 1.06E-05 | 2.76E-06 | 1.26E-05 | 2.68E-04 | 2.71E-04 | 2.68E-04 | 1.06E-05 | 2.76E-06 | 1.26E-05 |
| Delphinidin 3-O-glucoside | 3.56E-03 | 3.31E-03 | 3.44E-03 | 0.00E+00 | 0.00E+00 | 1.84E-04 | 5.55E-03 | 5.10E-03 | 5.80E-03 | 0.00E+00 | 0.00E+00 | 1.84E-04 | 1.03E-03 | 1.02E-03 | 1.03E-03 | 0.00E+00 | 0.00E+00 | 1.84E-04 |
| Peonidin 3-O-arabinoside | 5.98E-05 | 5.97E-05 | 6.33E-05 | 1.70E-05 | 1.78E-06 | 6.48E-06 | 8.64E-05 | 8.68E-05 | 8.45E-05 | 1.70E-05 | 1.78E-06 | 6.48E-06 | 2.22E-05 | 1.94E-05 | 2.22E-05 | 1.70E-05 | 1.78E-06 | 6.48E-06 |
| Cyanidin 3-O-(6-O-malonyl-beta-D-glucoside) | 9.60E-05 | 9.33E-05 | 9.91E-05 | 0.00E+00 | 0.00E+00 | 0.00E+00 | 6.84E-05 | 7.32E-05 | 8.31E-05 | 0.00E+00 | 0.00E+00 | 0.00E+00 | 3.03E-05 | 3.03E-05 | 3.03E-05 | 0.00E+00 | 0.00E+00 | 0.00E+00 |
| Delphinidin 3-O-rutinoside | 1.36E-06 | 1.35E-06 | 1.40E-06 | 4.18E-07 | 4.67E-07 | 7.83E-07 | 1.75E-06 | 1.50E-06 | 1.74E-06 | 4.18E-07 | 4.67E-07 | 7.83E-07 | 1.25E-06 | 1.06E-06 | 1.25E-06 | 4.18E-07 | 4.67E-07 | 7.83E-07 |
| Petunidin 3-O-galactoside | 2.80E-05 | 2.81E-05 | 3.22E-05 | 0.00E+00 | 0.00E+00 | 7.42E-07 | 7.61E-05 | 6.50E-05 | 7.76E-05 | 0.00E+00 | 0.00E+00 | 7.42E-07 | 1.14E-05 | 1.01E-05 | 1.14E-05 | 0.00E+00 | 0.00E+00 | 7.42E-07 |
| Petunidin 3-O-glucoside | 1.16E-05 | 1.11E-05 | 1.10E-05 | 3.59E-07 | 0.00E+00 | 8.76E-07 | 1.74E-05 | 1.50E-05 | 1.81E-05 | 3.59E-07 | 0.00E+00 | 8.76E-07 | 3.49E-06 | 3.35E-06 | 3.39E-06 | 3.59E-07 | 0.00E+00 | 8.76E-07 |
| Pelargonidin 3-O-(6-O-malonyl-beta-D-glucoside) | 3.05E-06 | 3.21E-06 | 3.49E-06 | 0.00E+00 | 0.00E+00 | 0.00E+00 | 2.77E-06 | 3.54E-06 | 3.62E-06 | 0.00E+00 | 0.00E+00 | 0.00E+00 | 8.78E-07 | 7.81E-07 | 8.78E-07 | 0.00E+00 | 0.00E+00 | 0.00E+00 |
| Pelargonidin 3-O-arabinoside | 4.50E-06 | 4.66E-06 | 4.91E-06 | 1.53E-07 | 9.14E-08 | 1.09E-07 | 1.08E-05 | 1.05E-05 | 1.04E-05 | 1.53E-07 | 9.14E-08 | 1.09E-07 | 1.55E-06 | 1.52E-06 | 1.55E-06 | 1.53E-07 | 9.14E-08 | 1.09E-07 |
| Delphinidin 3-O-(6''-O-malonyl)-beta-D-glucoside | 3.17E-05 | 2.77E-05 | 3.23E-05 | 3.29E-07 | 0.00E+00 | 0.00E+00 | 1.64E-05 | 1.63E-05 | 1.84E-05 | 3.29E-07 | 0.00E+00 | 0.00E+00 | 3.21E-06 | 3.80E-06 | 3.21E-06 | 3.29E-07 | 0.00E+00 | 0.00E+00 |
| Cyanidin 3-O-rutinoside | 2.48E-07 | 2.39E-07 | 3.08E-07 | 6.92E-08 | 0.00E+00 | 0.00E+00 | 4.97E-07 | 5.16E-07 | 5.81E-07 | 6.92E-08 | 0.00E+00 | 0.00E+00 | 0.00E+00 | 0.00E+00 | 0.00E+00 | 6.92E-08 | 0.00E+00 | 0.00E+00 |
| Malvidin 3-O-galactoside | 5.58E-06 | 5.83E-06 | 6.01E-06 | 0.00E+00 | 0.00E+00 | 0.00E+00 | 2.52E-05 | 2.68E-05 | 2.74E-05 | 0.00E+00 | 0.00E+00 | 0.00E+00 | 2.23E-06 | 1.93E-06 | 2.23E-06 | 0.00E+00 | 0.00E+00 | 0.00E+00 |
| Peonidin 3-O-(6-O-malonyl-beta-D-glucoside) | 1.05E-06 | 1.04E-06 | 1.10E-06 | 0.00E+00 | 0.00E+00 | 0.00E+00 | 5.27E-07 | 6.54E-07 | 6.90E-07 | 0.00E+00 | 0.00E+00 | 0.00E+00 | 4.20E-07 | 3.72E-07 | 4.20E-07 | 0.00E+00 | 0.00E+00 | 0.00E+00 |
| Delphinidin 3,5-O-diglucoside(Delphin) | 5.50E-05 | 4.88E-05 | 5.49E-05 | 0.00E+00 | 0.00E+00 | 0.00E+00 | 8.83E-05 | 9.34E-05 | 9.29E-05 | 0.00E+00 | 0.00E+00 | 0.00E+00 | 2.05E-05 | 1.90E-05 | 1.88E-05 | 0.00E+00 | 0.00E+00 | 0.00E+00 |
| Malvidin 3-O-arabinoside | 5.36E-07 | 5.43E-07 | 5.15E-07 | 0.00E+00 | 0.00E+00 | 0.00E+00 | 1.71E-06 | 1.60E-06 | 1.78E-06 | 0.00E+00 | 0.00E+00 | 0.00E+00 | 1.91E-07 | 1.59E-07 | 1.91E-07 | 0.00E+00 | 0.00E+00 | 0.00E+00 |
| Petunidin 3-O-arabinoside | 2.90E-06 | 3.13E-06 | 3.35E-06 | 0.00E+00 | 0.00E+00 | 0.00E+00 | 7.27E-06 | 6.99E-06 | 7.16E-06 | 0.00E+00 | 0.00E+00 | 0.00E+00 | 1.45E-06 | 1.14E-06 | 1.45E-06 | 0.00E+00 | 0.00E+00 | 0.00E+00 |
| Petunidin 3-O-(6-O-malonyl-beta-D-glucoside) | 1.73E-06 | 1.38E-06 | 1.33E-06 | 0.00E+00 | 0.00E+00 | 0.00E+00 | 1.07E-06 | 9.41E-07 | 9.25E-07 | 0.00E+00 | 0.00E+00 | 0.00E+00 | 0.00E+00 | 0.00E+00 | 0.00E+00 | 0.00E+00 | 0.00E+00 | 0.00E+00 |

**Table S5.** Transcriptome data quality and mapping information

| Samples | Total Reads | Mapped Reads | Uniq Mapped Reads | Multiple Map Reads | Clean reads | Clean bases | GC Content | %≥Q30 |
| --- | --- | --- | --- | --- | --- | --- | --- | --- |
| Jr-SG-1-1 | 48,438,004 | 45,586,586 (94.11%) | 42,168,413 (87.06%) | 3,418,173 (7.06%) | 24,219,002 | 7,243,831,034 | 47.46% | 95.80% |
| Jr-SG-1-2 | 47,508,468 | 44,218,004 (93.07%) | 40,896,627 (86.08%) | 3,321,377 (6.99%) | 23,754,234 | 7,099,430,692 | 47.48% | 95.46% |
| Jr-SG-1-3 | 51,388,058 | 47,653,629 (92.73%) | 44,352,097 (86.31%) | 3,301,532 (6.42%) | 25,694,029 | 7,669,811,290 | 47.45% | 95.67% |
| Jr-SG-2-1 | 57,833,170 | 54,116,096 (93.57%) | 49,928,398 (86.33%) | 4,187,698 (7.24%) | 28,916,585 | 8,644,827,142 | 47.37% | 95.48% |
| Jr-SG-2-2 | 46,162,588 | 42,575,122 (92.23%) | 39,179,591 (84.87%) | 3,395,531 (7.36%) | 23,081,294 | 6,901,057,878 | 47.62% | 95.40% |
| Jr-SG-2-3 | 50,026,542 | 46,675,752 (93.30%) | 43,275,727 (86.51%) | 3,400,025 (6.80%) | 25,013,271 | 7,480,256,338 | 47.45% | 95.43% |
| Jr-SG-3-1 | 44,386,476 | 41,006,185 (92.38%) | 37,989,524 (85.59%) | 3,016,661 (6.80%) | 22,193,238 | 6,641,257,314 | 47.50% | 95.23% |
| Jr-SG-3-2 | 44,956,344 | 41,759,961 (92.89%) | 38,541,950 (85.73%) | 3,218,011 (7.16%) | 22,478,172 | 6,719,010,808 | 47.78% | 95.27% |
| Jr-SG-3-3 | 47,011,944 | 44,191,041 (94.00%) | 40,793,354 (86.77%) | 3,397,687 (7.23%) | 23,505,972 | 7,018,904,012 | 47.45% | 95.44% |
| Jr-SR-1-1 | 62,191,784 | 59,088,221 (95.01%) | 54,400,772 (87.47%) | 4,687,449 (7.54%) | 31,095,892 | 9,302,393,122 | 47.45% | 95.53% |
| Jr-SR-1-2 | 53,125,670 | 50,611,456 (95.27%) | 46,644,101 (87.80%) | 3,967,355 (7.47%) | 26,562,835 | 7,945,724,930 | 47.49% | 95.84% |
| Jr-SR-1-3 | 48,730,032 | 45,627,480 (93.63%) | 42,124,199 (86.44%) | 3,503,281 (7.19%) | 24,365,016 | 7,283,661,578 | 47.50% | 95.90% |
| Jr-SR-2-1 | 50,195,350 | 46,841,533 (93.32%) | 43,525,263 (86.71%) | 3,316,270 (6.61%) | 25,097,675 | 7,495,433,166 | 47.60% | 95.58% |
| Jr-SR-2-2 | 43,291,604 | 40,971,380 (94.64%) | 37,853,664 (87.44%) | 3,117,716 (7.20%) | 21,645,802 | 6,472,690,110 | 47.60% | 95.50% |
| Jr-SR-2-3 | 48,733,196 | 45,652,190 (93.68%) | 42,485,896 (87.18%) | 3,166,294 (6.50%) | 24,366,598 | 7,286,235,310 | 47.53% | 95.22% |
| Jr-SR-3-1 | 49,103,064 | 46,350,248 (94.39%) | 42,878,895 (87.32%) | 3,471,353 (7.07%) | 24,551,532 | 7,336,508,836 | 47.16% | 95.34% |
| Jr-SR-3-2 | 55,305,866 | 51,465,770 (93.06%) | 47,344,846 (85.61%) | 4,120,924 (7.45%) | 27,652,933 | 8,261,977,748 | 47.62% | 95.58% |
| Jr-SR-3-3 | 48,342,068 | 45,053,887 (93.20%) | 41,284,948 (85.40%) | 3,768,939 (7.80%) | 24,171,034 | 7,211,731,660 | 47.69% | 95.75% |

**Table S6.** List of MYB protein family members in walnut and other species related to anthocyanin biosynthesis.

| **Protein name** | **Protein ID** |
| --- | --- |
| PhDPL | ADW94950.1 |
| PhPHZ | ADW94951.1 |
| PhMYBb  PhAN4  PhAN2  MdMYB1  MdMYB10  GtMYB3  SlANT1  CaMYBA  AtMYB113  AtPAP2  AtPAP1  AvMYB  AvROSEA2  AvROSEA1 | QUE39304.1  ADQ00392.1  AAF66727.1  XP_028963316.1  NP_001315777.1  BAF96933.1  NP_001234417.1  NP_001311547.1  NP_176811.1  NP_176813.1  NP_176057.1  ABB83828.1  ABB83827.1  ABB83826.1 |
| ZmMYBC1  VvMYB1a  OsMYBC1  IbMYB1  GhMYB10  CoMYB10  EjMYB10  PcMYB10  PaMYB10  PdMYB10  AcMYB10  FaMYB10  RhMYB10  CrRuby  PpMYB10-1  PpMYB10-2  PpMYB10-3  PpMYB10-4  PpMYB10-5  PpMYB10-6  PpMYB10-7  AtMYB16  MdMYBA  PpMYB10  MdMYB111  PaMYB308  MdMYB110a  PyMYB10  MrMYB1  VvMYBA1  VvMYBA2  FaMYB1(2)  PeMYB6  AnMYB  AtMYB4  AtMYB5  MrMYB39  PtMYB16  DkMYB4  AtTT2  MdMYB16  MdMYB6  VvMYB4-Like  VvMYBC2-L1  VvMYBC2-L3  PpMYB17  PpMYB18  PpMYB19  PpMYB20  FaMYB1  FcMYB1  JrDIVARICATA1  JrDIVARICATA2  JrDIVARICATA3  JrDIVARICATA4  JrDUO1a  JrDUO1b  JrGAMYB  JrHv33  JrLAF1  JrMYB0  JrMYB1  JrMYB101  JrMYB105  JrMYB106a  JrMYB106b  JrMYB106c  JrMYB108a  JrMYB108b  JrMYB111  JrMYB113  JrMYB114  JrMYB114b  JrMYB120  JrMYB123  JrMYB124a  JrMYB124b  JrMYB13a  JrMYB13b  JrMYB13c  JrMYB13d  JrMYB14  JrMYB16  JrMYB17a  JrMYB17b  JrMYB1a  JrMYB1aa  JrMYB1b  JrMYB1c  JrMYB1d  JrMYB2  JrMYB20  JrMYB21  JrMYB24a  JrMYB24b  JrMYB26a  JrMYB26b  JrMYB30  JrMYB306a  JrMYB308b  JrMYB308c  JrMYB308d  JrMYB308e  JrMYB308f  JrMYB315a  JrMYB315b  JrMYB33  JrMYB330a  JrMYB330b  JrMYB36  JrMYB39a  JrMYB39b  JrMYB3a  JrMYB3b  JrMYB3c  JrMYB3R-1a  JrMYB3R-1b  JrMYB3R-3  JrMYB41a  JrMYB41b  JrMYB44a  JrMYB44b  JrMYB44c  JrMYB44d  JrMYB44e  JrMYB44f  JrMYB46a  JrMYB46b  JrMYB48a  JrMYB48b  JrMYB48c  JrMYB4a  JrMYB4b  JrMYB4c  JrMYB4d  JrMYB4e  JrMYB4f  JrMYB5  JrMYB52  JrMYB54  JrMYB56  JrMYB60a  JrMYB60b  JrMYB61  JrMYB62a  JrMYB62b  JrMYB64  JrMYB6a  JrMYB6b  JrMYB6c  JrMYB6d  JrMYB6e  JrMYB6f  JrMYB8  JrMYB83a  JrMYB83b  JrMYB83c  JrMYB86  JrMYB93  JrMYB98a  JrMYB98b  JrMYB98c  JrMYBS1  JrODO1a  JrODO1b  JrODO1c  JrODO1d  JrRAX2a  JrRAX2b  JrRAX2c  JrRAX2d  JrRAX3a  JrRAX3b  JrRAX3c  JrRAX3d  JrSAM1  JrTT2  JrWER1  JrWER2  JrWER3  JrWER4  JrWER5  JrZm38  JrMYB306b  JrMYB308a | AAO85386.1  ABB87014.1  ADW40536.1  QMP81300.1  CAD87010.1  ABX71483.1  ABX71484.1  ABX71487.1  ABX71490.1  ABX71492.1  ABX79944.1  ABX79947.1  ABX79949.1  NP_001275818.1  XP_007216530.1  XP_007216350.1  XP_007216223.1  XP_020420992.1  XP_020420994.1  XP_020422250.1  XP_007205727.1  NP_197035.1  XP_028963316.1  ADN26574.1  NP_001281006.1  XP_021812021.1  AFC88038.1  ADN26574.1  ADG21957.1  XP_010664911.1  BAD18978.1  NP_176811.1  XP_007204546.1  Q8S9H7.1  BAA21619.1  NP_187963.1  KAB1223011.1  PNS95923.2  BAI49721.1  ABK28720.1  NP_001315806.1  NP_001315650.1  XP_002273328  ABW34393  KM046932  KT159233  KT159234  KT159235  KT159236  AF401220  GQ867222  XP_018821689.1  XP_018825920.1  XP_035541979.1  XP_018810775.2  XP_018819973.1  XP_018854309.1  XP_018833126.1  XP_018846121.1  XP_018830637.1  XP_018822492.1  XP_018843194.2  XP_018808993.1  XP_018845242.2  XP_018847369.1  XP_018813959.1  XP_018812743.1  XP_018825593.1  XP_018848935.1  XP_018857134.1  XP_018843189.2  XP_018833433.1  XP_018816729.1  XP_018811701.1  KAG6727586.1  XP_035540539.1  XP_018857008.1  XP_018838196.1  XP_018821204.1  XP_018807659.1  XP_018822668.2  XP_018809383.2  XP_018823177.2  XP_018830651.1  XP_018829462.1  XP_018816575.1  XP_018844016.1  XP_018825594.1  XP_018821900.1  XP_018821900.1  XP_018846808.1  XP_018816562.1  XP_018827465.2  XP_018852835.1  XP_018848606.2  XP_018827518.1  XP_018821528.1  XP_018818400.1  XP_018817045.1  XP_018841467.1  XP_018844625.1  XP_018818389.1  XP_018813001.1  XP_018857365.2  XP_018814315.1  XP_018847148.1  XP_018849363.1  XP_035545147.1  KAF5459709.1  XP_018820836.1  XP_018856985.2  XP_018806832.2  XP_018825037.1  XP_018819231.1  XP_018807150.2  XP_018845553.2  XP_018805614.1  XP_018831346.1  XP_018815706.2  KAF5460337.1  XP_018827388.1  XP_018819887.1  XP_018845037.1  XP_018805440.1  KAF5453693.1  XP_018805688.1  KAF5475354.1  XP_018830879.1  XP_035545073.1  XP_035545886.1  KAF5447011.1  XP_018827327.1  XP_018808219.1  XP_018820713.1  XP_018835066.2  XP_018821927.1  XP_018808176.1  XP_018809429.1  XP_018833403.1  XP_018849111.1  XP_018846633.2  XP_018822677.1  XP_018807003.1  XP_018832931.1  XP_018849697.1  XP_018811423.1  XP_018812796.1  XP_018816947.1  XP_035541943.1  XP_035548540.1  XP_018857096.2  XP_018859205.1  XP_018850674.2  XP_018821928.1  XP_018816716.1  XP_018834431.1  XP_018813213.1  XP_018805469.2  XP_018834458.1  XP_018822991.2  KAF5476789.1  XP_018807614.2  XP_018825411.2  XP_018847962.1  XP_018831444.1  XP_018815883.1  XP_018851960.1  XP_018832373.1  XP_018856949.1  XP_018858775.1  XP_018834527.1  XP_035551462.1  XP_018833816.1  XP_018860365.2  XP_018842498.1  XP_018807816.1  XP_018826719.1  XP_018831400.2  XP_018851468.1  XP_018821248.1  XP_018815053.1  XP_018817165.2  KAF5452952.1  XP_018812236.1  XP_035542199.1 |

Table S7. Correlation analysis between 31 core genes and 3 anthocyanins and 1 PA.

| Metabolites | Genes | PCC |
| --- | --- | --- |
| Delphinidin 3-O-galactoside | gene21099 | 0.907506047 |
|  | gene38150 | -0.844676245 |
|  | gene640 | -0.835182463 |
|  | gene24302 | 0.931169293 |
|  | gene36923 | 0.875392481 |
|  | gene39336 | -0.89419084 |
|  | gene1297 | -0.95146824 |
|  | gene35863 | 0.873037777 |
|  | gene4994 | 0.932111149 |
|  | gene32601 | 0.811200213 |
|  | gene35144 | 0.897976206 |
|  | gene42522 | -0.941720283 |
|  | gene35146 | 0.928004446 |
|  | gene40343 | -0.932777545 |
|  | gene1697 | 0.813418854 |
|  | gene35048 | -0.830388731 |
|  | gene40994 | -0.869388581 |
|  | gene1870 | -0.8013569 |
|  | gene38312 | -0.827964965 |
|  | gene1567 | 0.912063987 |
|  | gene9445 | 0.843844147 |
|  | gene5786 | -0.938069458 |
|  | gene32351 | 0.825796271 |
|  | gene32032 | 0.839797338 |
|  | gene42048 | 0.880197538 |
|  | gene24750 | 0.909368939 |
| Cyanidin 3-O-galactoside | gene24378 | -0.801161606 |
|  | gene640 | 0.876219783 |
|  | gene36923 | -0.935579496 |
|  | gene1297 | 0.831873066 |
|  | gene4994 | -0.895193591 |
|  | gene28510 | -0.833703482 |
|  | gene32601 | -0.939682037 |
|  | gene35144 | -0.863065374 |
|  | gene42522 | 0.909947184 |
|  | gene35146 | -0.820376478 |
|  | gene1697 | -0.803425911 |
|  | gene1870 | 0.901472924 |
|  | gene1567 | -0.915542479 |
|  | gene9445 | -0.940537516 |
|  | gene5786 | 0.924748961 |
|  | gene42048 | -0.808786843 |
|  | gene24750 | -0.826991327 |
| Delphinidin 3-O-arabinoside | gene21099 | -0.896056147 |
|  | gene24378 | -0.841955662 |
|  | gene38150 | 0.87844263 |
|  | gene640 | 0.846615359 |
|  | gene36923 | -0.95458527 |
|  | gene4994 | -0.919667699 |
|  | gene28510 | -0.800070478 |
|  | gene32601 | -0.935372941 |
|  | gene35144 | -0.891060148 |
|  | gene42522 | 0.884204707 |
|  | gene35146 | -0.852336727 |
|  | gene35048 | 0.836092937 |
|  | gene1870 | 0.874688353 |
|  | gene1567 | -0.93744692 |
|  | gene9445 | -0.906525535 |
|  | gene5786 | 0.900973209 |
|  | gene24750 | -0.859672958 |
| Cyanidin 3-O-glucoside | gene21099 | -0.810286501 |
|  | gene24302 | -0.883198555 |
|  | gene36923 | -0.867107967 |
|  | gene39336 | 0.851287622 |
|  | gene1297 | 0.909883909 |
|  | gene35863 | -0.874443312 |
|  | gene4994 | -0.81244619 |
|  | gene28510 | -0.911250656 |
|  | gene32601 | -0.836253175 |
|  | gene42522 | 0.924259648 |
|  | gene4387 | 0.881844545 |
|  | gene40343 | 0.885270482 |
|  | gene1697 | -0.888266295 |
|  | gene35048 | 0.810251797 |
|  | gene40994 | 0.890022201 |
|  | gene1870 | 0.850332135 |
|  | gene38312 | 0.867557272 |
|  | gene1567 | -0.839479542 |
|  | gene5786 | 0.932793764 |
|  | gene32351 | -0.875320817 |
|  | gene39085 | 0.829435295 |
|  | gene32032 | -0.860953566 |
|  | gene29715 | -0.858715846 |
|  | gene42048 | -0.892391004 |
|  | gene24750 | -0.896495673 |
| Procyanidin C1 | gene21099 | -0.945052741 |
|  | gene24378 | -0.914488096 |
|  | gene38150 | 0.905502361 |
|  | gene640 | 0.917971758 |
|  | gene24302 | -0.850578435 |
|  | gene36923 | -0.897876187 |
|  | gene1297 | 0.880752816 |
|  | gene4994 | -0.848894468 |
|  | gene42522 | 0.897265884 |
|  | gene40343 | 0.852902882 |
|  | gene1697 | -0.818542783 |
|  | gene35048 | 0.801235839 |
|  | gene40994 | 0.858244402 |
|  | gene38312 | 0.83311439 |
|  | gene1567 | -0.873272709 |
|  | gene9445 | -0.8314077 |
|  | gene5786 | 0.834773749 |
|  | gene32351 | -0.841764917 |
|  | gene39085 | 0.948513961 |
|  | gene32032 | -0.825781601 |
|  | gene29715 | -0.823301823 |
|  | gene42048 | -0.860913002 |
|  | gene24750 | -0.865546706 |

Table S8. Correlation analysis between 10 TFs and 21 structural genes.

| TFs | Structural genes | PCC |
| --- | --- | --- |
| JrMYB1b  (gene38312) | gene38150 | 0.908552517 |
|  | gene640 | 0.828418062 |
|  | gene24302 | -0.934880013 |
|  | gene39336 | 0.91113512 |
|  | gene1297 | 0.922272589 |
|  | gene35863 | -0.935118551 |
|  | gene4387 | 0.948538197 |
|  | gene40343 | 0.840645277 |
|  | gene1697 | -0.927032559 |
|  | gene40994 | 0.896393511 |
|  | gene1870 | 0.92348726 |
| JrMYB123  (gene9445) | gene21099 | 0.921861366 |
|  | gene24378 | 0.834537517 |
|  | gene36923 | 0.827351346 |
|  | gene4994 | 0.895281995 |
|  | gene28510 | 0.921617682 |
|  | gene32601 | 0.930950948 |
|  | gene35144 | 0.847013362 |
|  | gene42522 | -0.857593496 |
|  | gene35146 | 0.971973945 |
| JrTT2  (gene39085) | gene21099 | -0.880309851 |
|  | gene24378 | -0.923711374 |
|  | gene38150 | 0.931725102 |
|  | gene640 | 0.935924292 |
|  | gene28510 | -0.865380175 |
|  | gene32601 | -0.868251529 |
|  | gene35146 | -0.930103622 |
| JrMYB6a  (gene32351) | gene24378 | 0.835326959 |
|  | gene38150 | -0.934916596 |
|  | gene640 | -0.915073795 |
|  | gene24302 | 0.931976079 |
|  | gene36923 | 0.84235835 |
|  | gene39336 | -0.903690927 |
|  | gene1297 | -0.927252032 |
|  | gene35863 | 0.896604772 |
|  | gene35144 | 0.82243519 |
|  | gene42522 | -0.810759117 |
|  | gene4387 | -0.913491588 |
|  | gene40343 | -0.89392647 |
|  | gene1697 | 0.930095702 |
|  | gene35048 | -0.869346222 |
|  | gene40994 | -0.934742334 |
|  | gene1870 | -0.910193329 |
| JrMYB308e  (gene29715) | gene24302 | 0.888908536 |
|  | gene39336 | -0.853905093 |
|  | gene1297 | -0.882962898 |
|  | gene4387 | -0.865884439 |
|  | gene40343 | -0.842097778 |
|  | gene1697 | 0.815012259 |
|  | gene35048 | -0.812838478 |
|  | gene40994 | -0.892412588 |
|  | gene1870 | -0.861837431 |
| WD40  (gene5786) | gene21099 | -0.819334283 |
|  | gene24378 | -0.932003059 |
|  | gene36923 | -0.927243591 |
|  | gene4994 | -0.882921679 |
|  | gene28510 | -0.818973873 |
|  | gene32601 | -0.832940666 |
|  | gene35144 | -0.940108732 |
|  | gene42522 | 0.946812099 |
|  | gene35146 | -0.900482989 |
|  | gene35048 | 0.906428595 |
| WD40  (gene32032) | gene24302 | 0.940847632 |
|  | gene36923 | 0.855720472 |
|  | gene39336 | -0.914232507 |
|  | gene1297 | -0.936426557 |
|  | gene35144 | 0.836561028 |
|  | gene42522 | -0.825306584 |
|  | gene4387 | -0.923490558 |
|  | gene40343 | -0.904977779 |
|  | gene35048 | -0.881568685 |
|  | gene40994 | -0.943431047 |
|  | gene1870 | -0.920378663 |
| WD40  (gene42048) | gene24302 | 0.964750001 |
|  | gene36923 | 0.894051816 |
|  | gene39336 | -0.943480025 |
|  | gene1297 | -0.961296938 |
|  | gene4994 | 0.825801483 |
|  | gene35144 | 0.877365759 |
|  | gene42522 | -0.867479619 |
|  | gene4387 | -0.950998844 |
|  | gene40343 | -0.93586145 |
|  | gene35048 | -0.916220493 |
|  | gene40994 | -0.820616874 |
|  | gene1870 | -0.94848402 |
| WD40  (gene24750) | gene24302 | 0.888928067 |
|  | gene36923 | 0.921449955 |
|  | gene39336 | -0.928900376 |
|  | gene1297 | -0.935223331 |
|  | gene4994 | 0.86093672 |
|  | gene35144 | 0.906884475 |
|  | gene42522 | -0.898174568 |
|  | gene4387 | -0.936816254 |
|  | gene40343 | -0.945651107 |
|  | gene35048 | -0.940476539 |
|  | gene40994 | -0.935001341 |
|  | gene1870 | -0.903522553 |
| WD40  gene1567 | gene21099 | 0.856770506 |
|  | gene24378 | 0.90491295 |
|  | gene36923 | 0.899340091 |
|  | gene4994 | 0.949909472 |
|  | gene28510 | 0.933499456 |
|  | gene32601 | 0.901186741 |
|  | gene35144 | 0.91448112 |
|  | gene42522 | -0.922479444 |
|  | gene35146 | 0.928221824 |
|  | gene35048 | -0.875284848 |
