# Supplementary material for: Integrated metabolomic and transcriptomic analysis of the anthocyanin and proanthocyanidin regulatory networks in red walnut natural hybrid progeny leaves
Source: PeerJ. 2022 Oct 20;10:e14262. doi: 10.7717/peerj.14262 (PMC9588303; doi:10.7717/peerj.14262)
Supplement: Supplemental Information 2 [file peerj-10-14262-s002.docx]

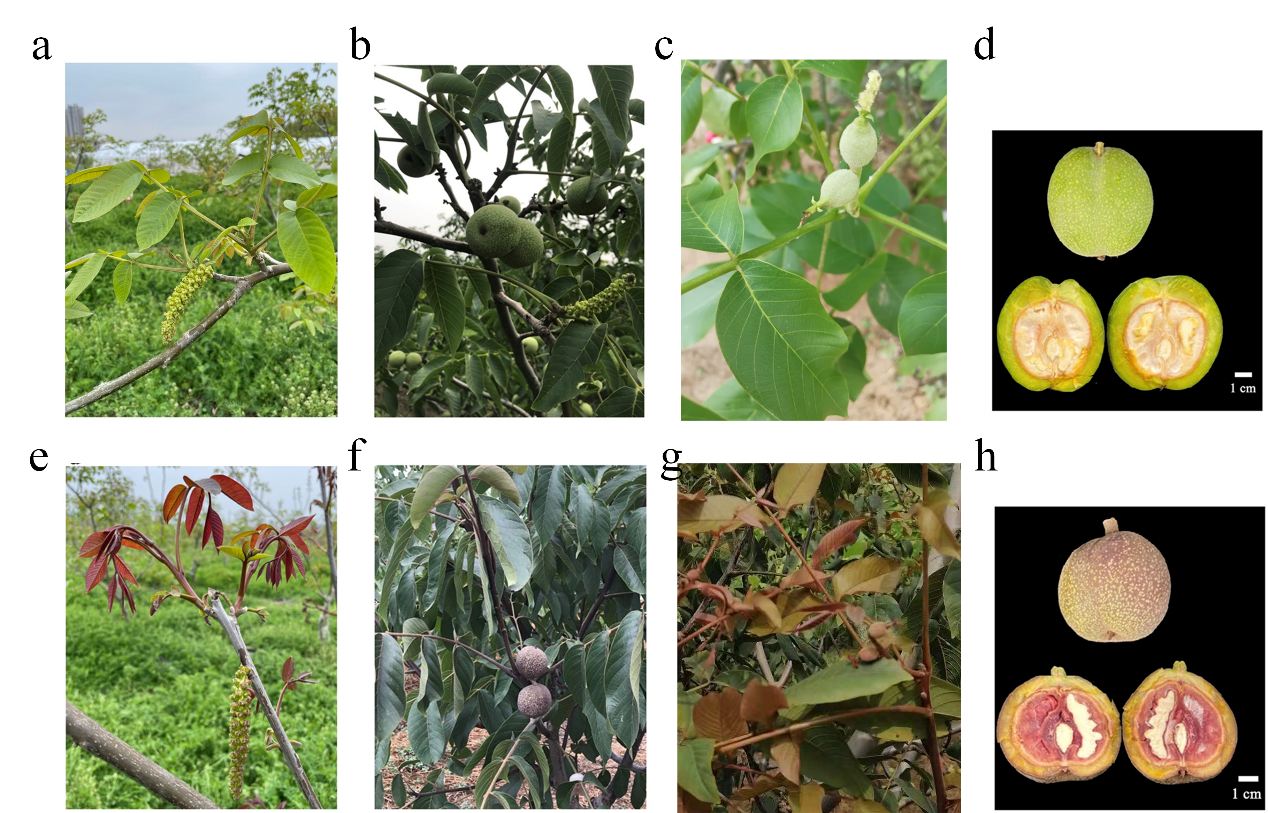


**Figure S1.** Phenotypic comparison between common green walnut 'Zhonglin 1' and red walnut ‘RW-1’. (a-d) Leaves, fruits, pistillate flowers and longitudinal section of fruit in 'Zhonglin 1'. (e-h) Leaves, fruits, pistillate flowers and longitudinal section of fruit of ‘RW-1’.


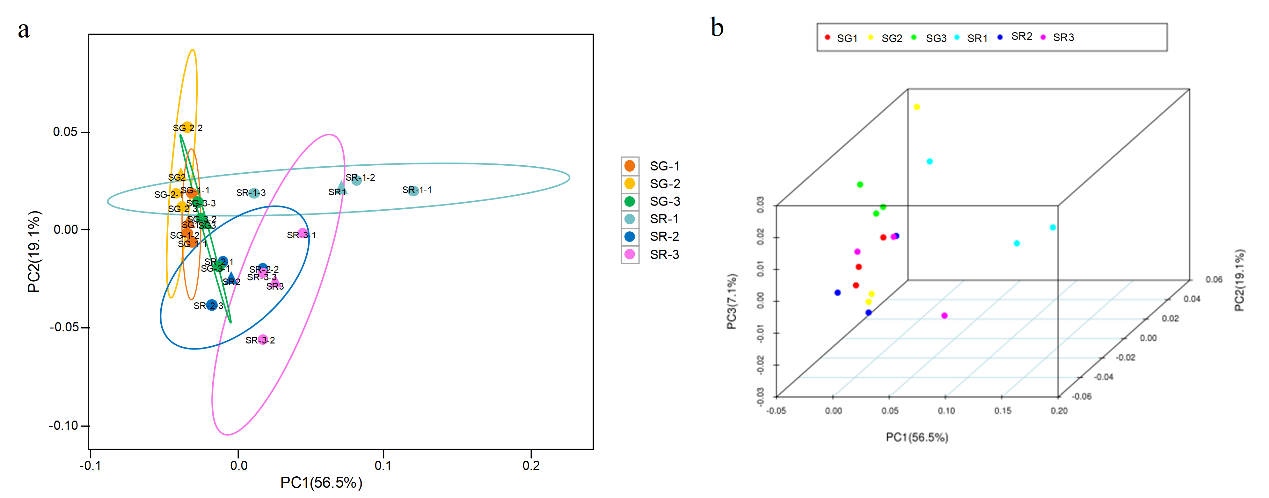


**Figure S2.** Two-dimensional and three-dimensional maps of principal component analysis results for metabolites in total samples.


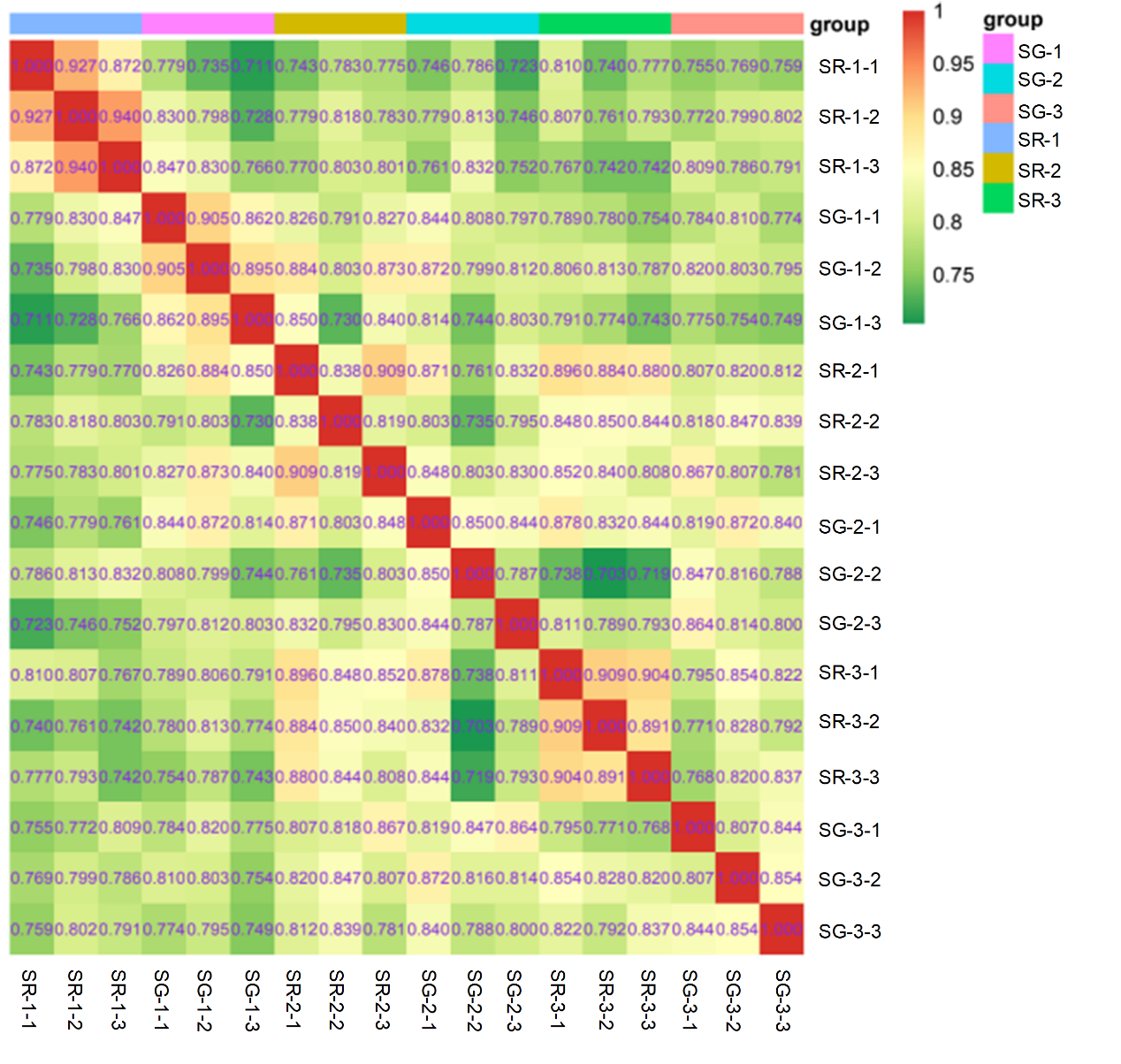


**Figure S3.** Correlation analysis of population samples. The heatmap represents the correlation coefficient between separate blade samples, and green to red in the heatmap indicates that the correlation coefficient ranges from low to high. SR: Seedling progenies-Red leaves; The leaves were collected according to the color change of SR walnut leaves, as full red period (SR-1), red–green period (SR-2), and full green period (SR-3), the leaves of SG walnut in the same period were collected as the control.

**
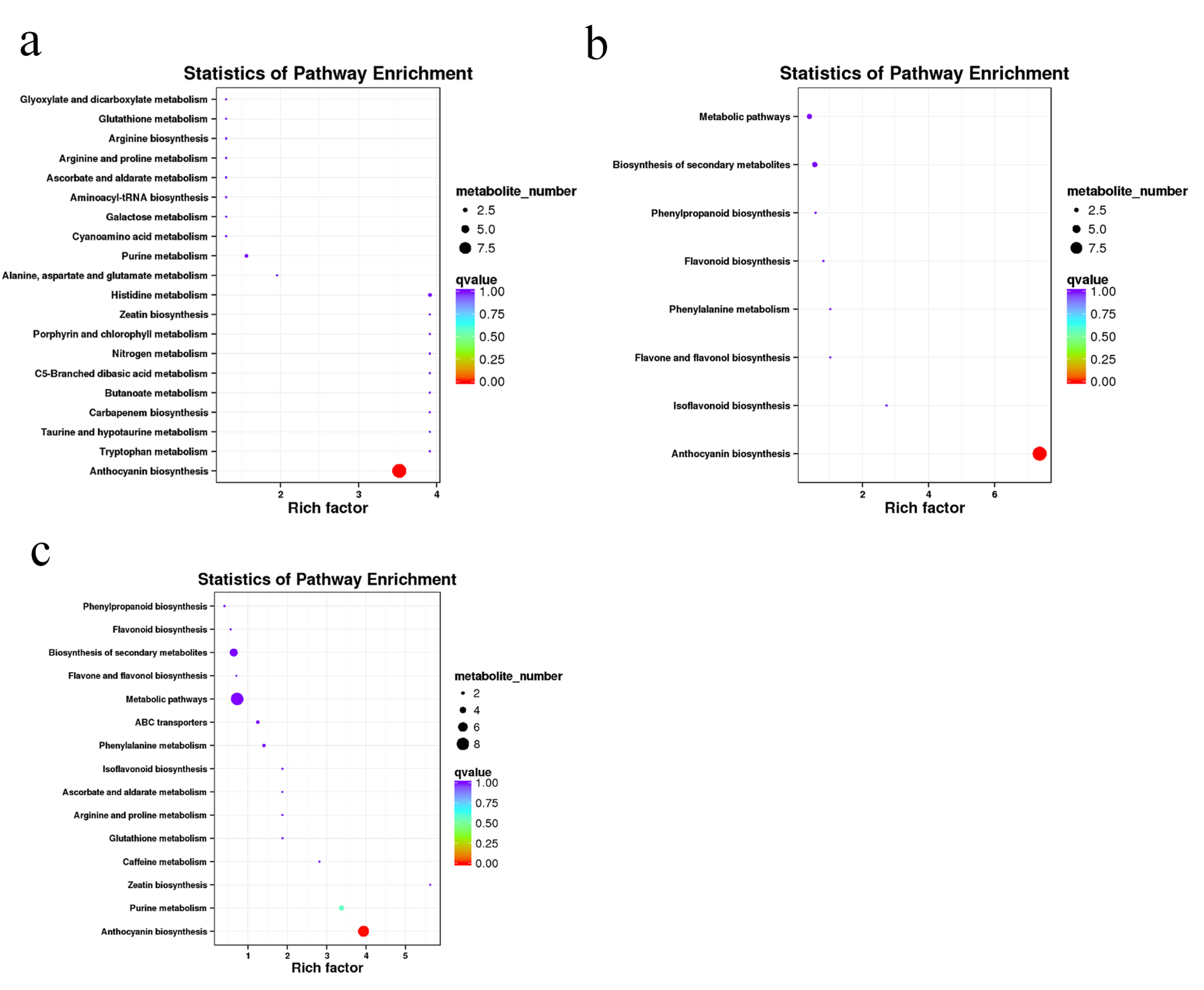
**

**Figure S4.** KEGG enrichment analysis of the DAMs between SG and SR. KEGG enrichment analysis of the DAMs between SG-1 vs. SR-1 (a), SG-2 vs. SR-2 (b) and SG-3 vs. SR-3 (c). The leaves were collected according to the color change of SR walnut leaves. During the full red period (SR-1), red–green period (SR-2), and full green period (SR-3), the leaves of SG walnut in the same period were collected as the control.


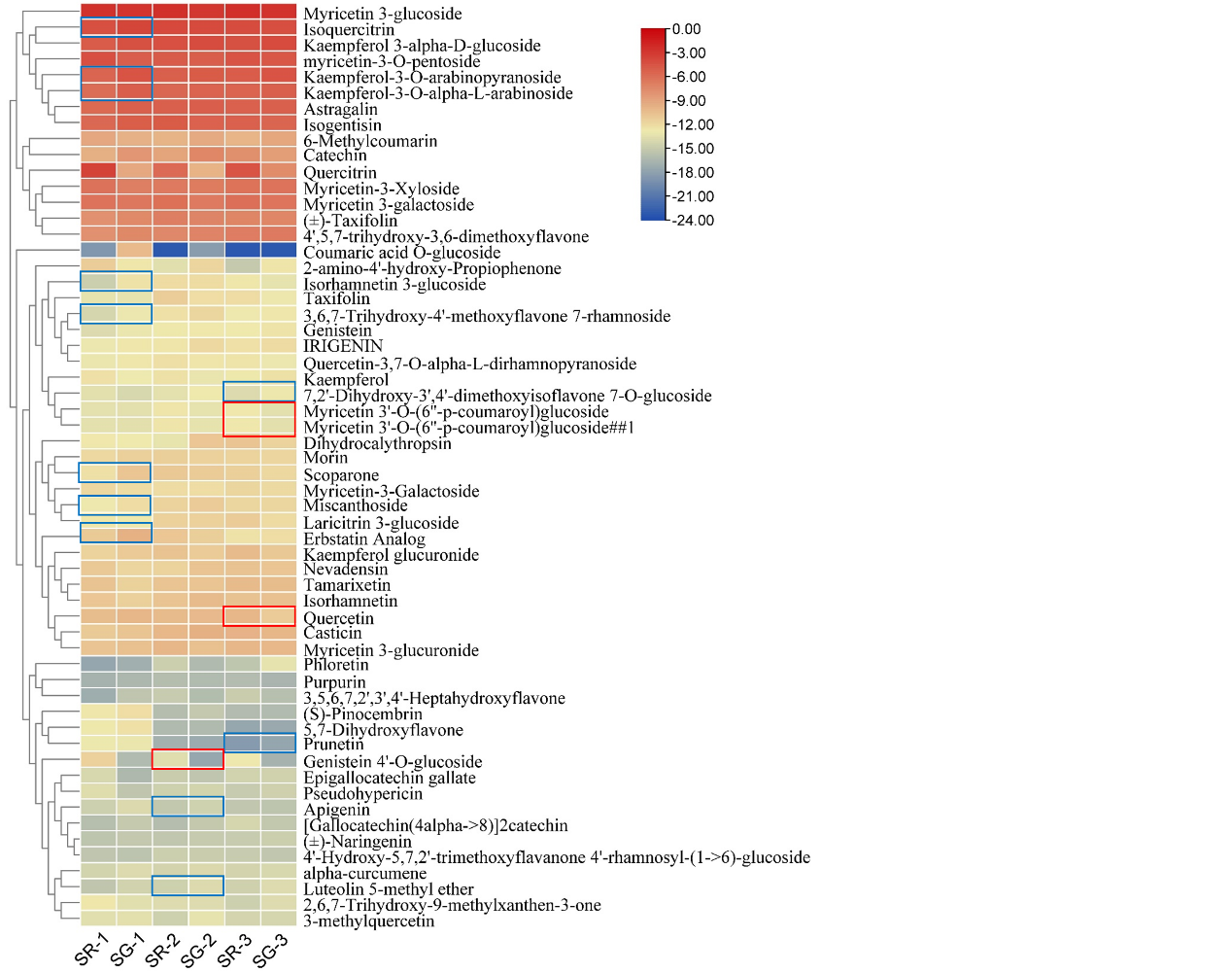


**Figure S5.** Concentrations of flavonoids and isoflavonoids in the leaves of SR and SG. SG: Seedling progenies-Green leaves; SR: Seedling progenies-Red leaves; The leaves were collected according to the color changing of SR walnut leaves, as full red period (SR-1), red–green period (SR-2), and full green period (SR-3), the leaves of SG walnut in the same period were collected as the control. The red frames indicate upregulated metabolites, and the blue frames indicate downregulated metabolites.

**
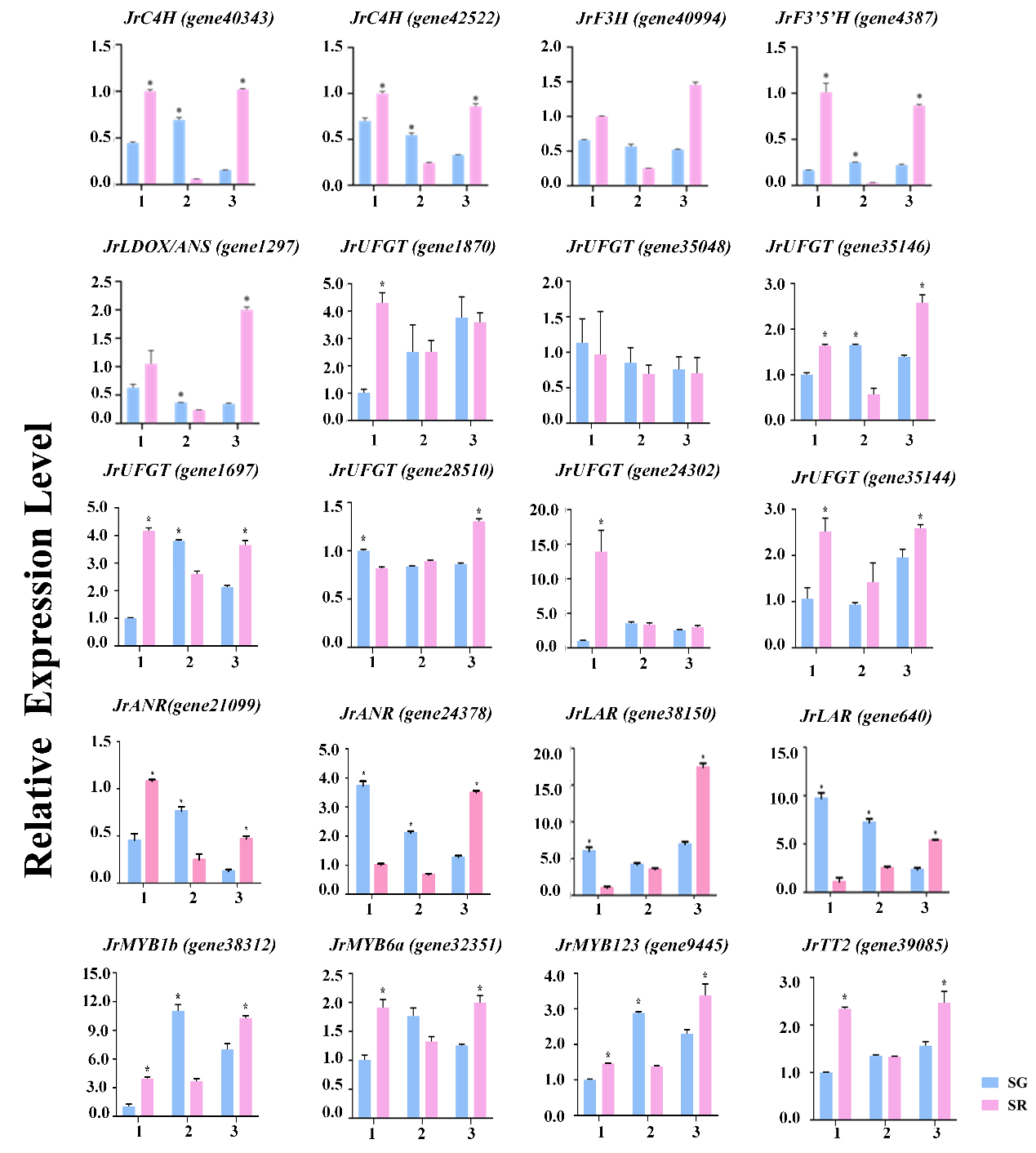
Figure S6.** Expression of structural genes and *MYB* genes in the natural hybrid progenies of red walnut with different phenotypes (red leaves and green leaves). The relative expressions were detected by qRT–PCR. Leaves of the various phenotypes (red leaves and green leaves) of red walnut natural hybrid progeny were collected at three stages. SG, Seedling progenies-Green leaves; SR, Seedling progenies-Red leaves. The leaves were collected according to the color change of SR walnut leaves. During the full red period (SR-1), red–green period (SR-2), and full green period (SR-3), the leaves of SG walnut in the same period were collected as the control. Significant differences were determined using a one-sided paired *t*-test (**p*< 0.05). Expression values (±SE) of three replicates were normalized using *Jr18s* as the internal control.


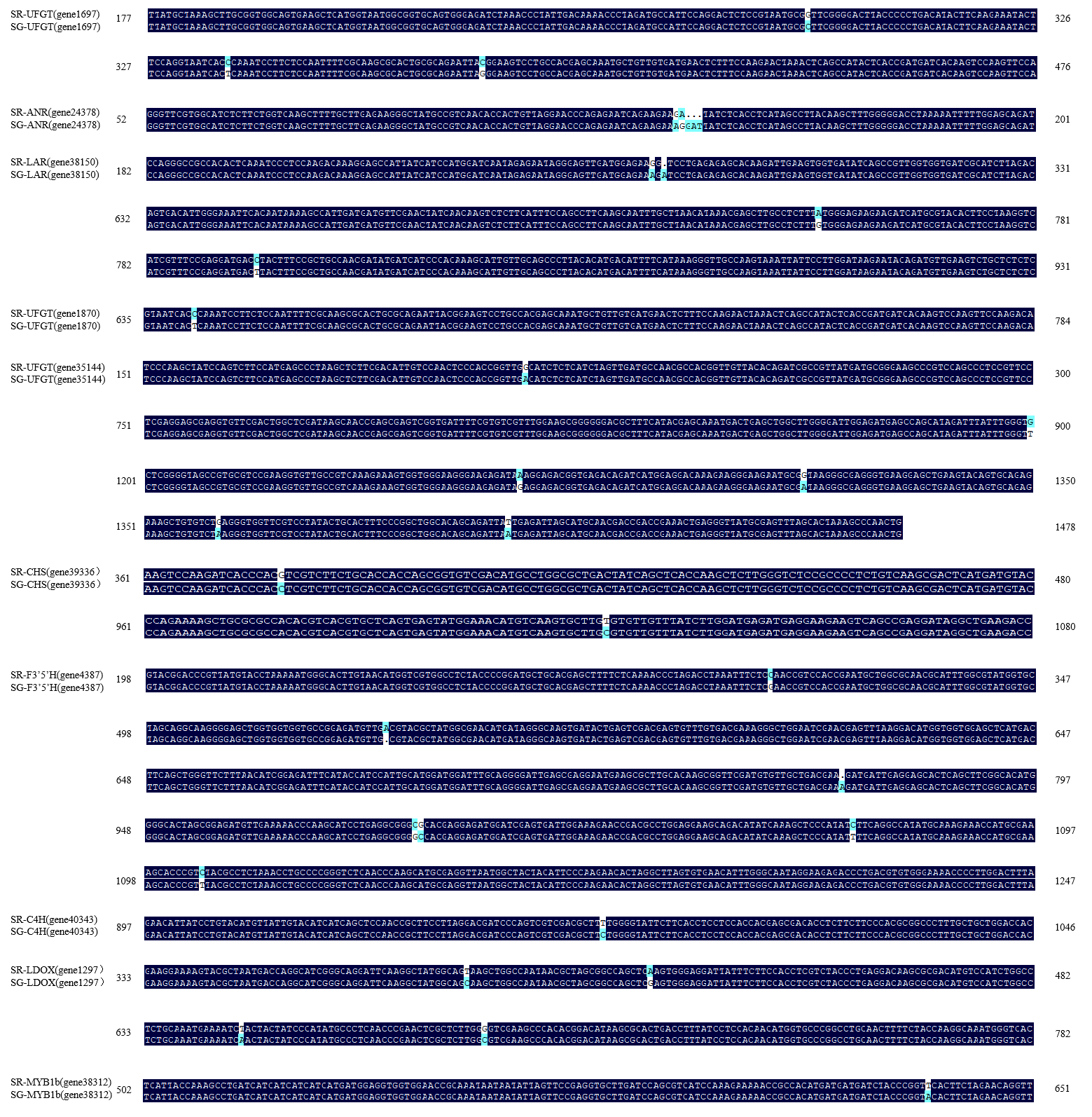


**Figure S7.** Amplification and sequence alignment of CDS region of structural genes and TF genes in the natural hybrid progenies of red walnut with different phenotypes (red leaves and green leaves). SG, Seedling progenies-Green leaves; SR, Seedling progenies-Red leaves.
